# Royal Jelly: Biological Action and Health Benefits

**DOI:** 10.3390/ijms25116023

**Published:** 2024-05-30

**Authors:** Nada Oršolić, Maja Jazvinšćak Jembrek

**Affiliations:** 1 Division of Animal Physiology, Faculty of Science, University of Zagreb, Rooseveltov trg 6, HR-10000 Zagreb, Croatia; 2 Division of Molecular Medicine, Laboratory for Protein Dynamics, Ruđer Bošković Institute, Bijenička cesta 54, HR-10000 Zagreb, Croatia; maja.jazvinscak.jembrek@irb.hr; 3 School of Medicine, Catholic University of Croatia, Ilica 242, HR-10000 Zagreb, Croatia

**Keywords:** royal jelly, bioactive components, aphytherapy, molecular and cellular activity

## Abstract

Royal jelly (RJ) is a highly nutritious natural product with great potential for use in medicine, cosmetics, and as a health-promoting food. This bee product is a mixture of important compounds, such as proteins, vitamins, lipids, minerals, hormones, neurotransmitters, flavonoids, and polyphenols, that underlie the remarkable biological and therapeutic activities of RJ. Various bioactive molecules like 10-hydroxy-2-decenoic acid (10-HDA), antibacterial protein, apisin, the major royal jelly proteins, and specific peptides such as apisimin, royalisin, royalactin, apidaecin, defensin-1, and jelleins are characteristic ingredients of RJ. RJ shows numerous physiological and pharmacological properties, including vasodilatory, hypotensive, antihypercholesterolaemic, antidiabetic, immunomodulatory, anti-inflammatory, antioxidant, anti-aging, neuroprotective, antimicrobial, estrogenic, anti-allergic, anti-osteoporotic, and anti-tumor effects. Moreover, RJ may reduce menopause symptoms and improve the health of the reproductive system, liver, and kidneys, and promote wound healing. This article provides an overview of the molecular mechanisms underlying the beneficial effects of RJ in various diseases, aging, and aging-related complications, with special emphasis on the bioactive components of RJ and their health-promoting properties. The data presented should be an incentive for future clinical studies that hopefully will advance our knowledge about the therapeutic potential of RJ and facilitate the development of novel RJ-based therapeutic opportunities for improving human health and well-being.

## 1. Introduction

### Royal Jelly as Functional Food

Royal jelly (RJ), a viscous product of the beehive, is appreciated as an attractive ingredient for healthy foods. It is secreted by the hypopharyngeal and mandibular glands of worker honeybees (*Apis mellifera*) and used as a food for the bee larvae and the queen. RJ is recognized for its exceptional nutritional value, earning the reputation of a “superfood” whose consumption has numerous health benefits for humans [1,2,3].

The method for producing RJ is based on artificial larvae grafting. RJ production occurs outside the honeycomb in artificial queen cells made of wax. Worker bee larvae, 12 to 18 h after hatching, are transferred into these cells using an inoculation pen to encourage the bee colony to produce RJ for larval feeding. After 68–72 h (3 days), the larvae are removed from the substrate with tweezers, and the RJ is collected and transferred to a storage bottle. This conventional method, which involves grafting young larvae, is time-consuming, labor-intensive, and limited by the availability of larvae and the technician’s vision. A new method for producing RJ eliminates the need for larvae grafting and utilizes a practical and efficient device. The device consists of a plastic worker foundation with regular holes, plastic cell bottoms mounted on a bar that can be inserted into the comb to fill the holes, and into the bottomless plastic queen cups on RJ production bars [3].

In recent years, consumers and the food industry have become increasingly aware of functional foods and how they can contribute to maintaining human health. The important role of a diet in the prevention and treatment of various illnesses is, nowadays, widely accepted. Functional foods can be natural or produced by extracting or replacing one or more ingredients. Furthermore, some ingredients, such as omega-3 fatty acids, vitamins, probiotics, fibers, bioactive peptides, and fitosterols, can be added to the foods to increase their “functionality” or “benefit” [1,2]. Besides RJ, foods with potential health benefits from the beehive include honey, propolis, beebread, and pollen [3,4]. It is recognized that various bee products, including RJ, can have a positive effect on human health. RJ contains a considerable number of essential components, such as proteins, free amino acids, lipids, vitamins, sugars, hormones, and bioactive substances like 10-HDA. Some of the most abundant proteins from RJ are proteins from the family of major royal jelly proteins (MRJPs), antibacterial protein, and 350-kDa protein called apisin, an unique component of RJ that consists of major royal jelly protein 1 (MRJP1) and apisimin [3].

RJ, as the most valued bee product, is not only one of the most attractive functional foods; it can also be used in medicine as a medicinal product. In many countries, it is recommended in pediatrics to geriatric medicine, especially in relation to nutrition and cosmetics. Due to the highly valuable nutritive composition, the consummation of RJ is constantly growing. It can be consumed in different forms, either native or as a functional component of different food products. According to some sources, the annual production of RJ in China, the world’s largest producer and exporter, is over 4000 tons, which represents more than 90% of the total production on a global level [5].

Regarding medicinal use, RJ and its biologically active components have gained significant interest due to their potential chemopreventive/protective functions in the maintenance of human health based on their anti-inflammatory and antioxidative properties, as well as food–gene interactions. Bioactive components of RJ, such as polyphenols, vitamins, hormones, and enzymes, are essential for redox reactions and controlling the level of oxidative stress (OS) and OS-induced cell damage and inflammation, factors that lead to the progression of symptoms associated with metabolic syndrome, cancer, aging, and neurodegenerative diseases. Various biological activities and health-promoting properties of RJ are largely assigned to diverse phenolic compounds and glycosides from the RJ, such as pinobanksin, hesperetin, kaempferol, isorhamnetin, isosakuranetin, naringenin, chrysin, acacetin, luteolin, apigenin, and formononetin. These findings directed pharmacological studies towards the beneficial health effects of various RJ components that are capable of interfering with the production of reactive radicals, reduce OS and inflammation, and potentially slow down the onset and progression of numerous chronic diseases.

Nowadays, RJ is being intensively tested, especially in Japan, and many of its effects have been scientifically confirmed. In addition to its use as a cosmetic and dietary supplement, it is considered that RJ has biostimulating and regenerative effects on human health based on its unique chemical composition. However, there is not yet enough data on the effectiveness of RJ and the health benefits for humans. The biological effects of RJ have mainly been studied in experimental animal models and cell cultures rather than in humans. Considering the complexity of RJ, the main approach is to purify and test the biological effectiveness of individual bioactive molecules.

The aim of this review is to collect the latest relevant studies on the use of RJ in the prevention and therapy of numerous diseases associated with oxidative stress and inflammation. These conditions result from the reduced antioxidant capacity of the organism, which contributes to the increase in chronic diseases, including neurological disorders, type 2 diabetes, cancer, aging, cardiovascular damage, acute kidney failure, hypertension, pre-eclampsia, osteoporosis, inflammatory diseases of the liver and intestines, and atherosclerosis, among others. Furthermore, our goal is to highlight the benefits of RJ and its effects on health-issue prevention in both the young and elderly population, especially related to its antimicrobial, antibacterial, immunoregulatory, antidiabetic, and reproductive health improvement properties, wound healing, life extension, slower aging, antilipidemic, antihypertensive, antiviral, and antiparasitic effects, the protection of vital organs such as the brain, heart, liver, and kidneys from toxins and drugs (organo-protective effects—neuroprotective, hepato-renal protective, etc.), neuromodulatory capabilities, stimulation of growth, regulation of healthy cells, antiobesity properties, and memory improvement. We believe that gathering knowledge about the latest molecular mechanisms and pharmacological targets of the active biological components of RJ will enable its wider application and production for the treatment of various diseases, ultimately improving the quality of life, especially for individuals suffering from specific illnesses. These novel findings will enhance the comprehensive understanding and use of RJ, making it more effective in maintaining health. Given the exceptional biological properties of RJ and its use in many areas, from the pharmaceutical and food industries to the manufacturing of cosmetic products, there is a need for its standardization, investigation of qualitative and quantitative properties, and analytic studies of bioactive substances and their transformations during storage and keeping.

## 2. Chemical Composition of Royal Jelly

RJ is made of 60–70% water; 9–18% proteins (*w*/*w)* (albumin, α, β, γ globulin, glycoproteins, lipoproteins, and 23 amino acids); 7–18% sugars (glucose, fructose, negligible amounts of ribose, maltose, isomaltose, trehalose, neotrehalos, gentiobiose, turanose, and inositol); 3–8% lipids (*w*/*w)* (sterols and glycerols, wax, neutral fats, fatty acids, phospholipids, phenolic lipids, and free organic acids), 0.7–1.5% minerals (K, Na, Ca, Mg, Cu, Fe, Mn, Zn, Si, Cr, Ni, Ag, Co, Al, As, Hg, Bi, Au, S, and P), and vitamins (B_1_, B_2_, B_3_, B_5_, B_6_, B_7_, B_9_, B_12_, E, D, A, K, and C, 336–351 mg/100 g) [2,3,5,6,7]. The chemical composition, especially the sugar content in RJ, is highly variable and depends on the geographical origin, plant species, bee species, season, and method of collection. RJ also contains various previously mentioned phenolic compounds, flavonoids, organic acids, enzymes (amylase, invertase, catalase, acid phosphatase, and others), neurotransmitter acetylcholine and its precursor choline, as well as sex hormones (estradiol, testosterone, and progesterone). In addition to proteins and peptides, RJ contains a large amount of free amino acids, such as lysine, proline, cystine, aspartic acid, valine, glutamic acid, serine, glycine, cysteine, threonine, alanine, tyrosine, phenylalanine, leucine, isoleucine, and glutamine. As mentioned, RJ is also rich in lipids that account for 15–30% of the lyophilized product. The lipid content predominantly encompasses fatty acids (more than 80%) that are followed by phenols (4–10%), waxes (5–6%), steroids (3–4%), and phospholipids (0.4–0.8%). The most abundant are medium-chain fatty acids (MCFAs), such as (10-HDA) sebacic acid and 9-hydroxy-2-decenoic acid. 10-HDA is the main constituent (app. 21 mg/g RJ) that is also regarded as a marker of quality and freshness [8,9,10,11].

In addition to phenolic compounds, pharmacological effects of RJ are also attributed to 10-HDA, royalisin, apisin, and some antimicrobial proteins [12,13]. According to Furusawa et al. [10], apisin is a hetero-oligomer containing MRJP1 (55 kDa protein) and apisimin (5 kDa protein). The apisin content in RJ is fairly constant (i.e., 3.93 to 4.67 *w*/*w*%) and can be used as a quality standard of RJ. The main compounds from RJ are shown in Table 1.

Proteins and peptides are the second most abundant components of RJ. The main proteins are MRJP1 (royalactin) to MRJP9 (more than 80% of the protein content, molecular weights between 49 to 87 kDa), whereas the most often-found peptides in RJ are apisimin, royalisin, apidaecin, defensin-1, and jelleins [13,14,15,16]. Proteins represent more than 50% of dry matter [13,14,15,16] and have a specific physiological role in queen bee development. MRJPs include numerous essential amino acids, like ovalbumin and casein [17,18,19,20,21,22]. Proteins from the MRJP family, from MRP1 to MRP9, are the main soluble proteins (31%) of RJ [17,18,19]. MRJP 1 is a weakly acidic glycoprotein (55 kDa) that forms oligomers of 350 or 420 kDa [19,20]. MRJP 2, MRJP3, MRJP4, and MRJP 5 are glycoproteins of 49 kDa, 60–70 kDa, 60 kDa, and 80 kDa, respectively) [22].

In one study that investigated the protein composition of RJ, 134 proteins were identified by gel-based and gel-free proteomics [20,22,23,24]. MRJPs were the main protein components of RJ, together with some proteins participating in the carbohydrate metabolism, such as glucose oxidase, predecessor-glucosidase, and glucose dehydrogenase. The study revealed 19 new proteins that were predominantly grouped in three functional categories: redox proteins, protein-binding proteins, and lipid-transporting proteins. The important physiological functions of RJ proteins have been demonstrated in numerous studies [16,17,18,19,20,21,22,23,24,25]. For example, some MRJPs stimulate cell proliferation [26,27,28,29,30,31,32,33,34], while others inhibited bisphenol A-induced proliferation of human breast cancer cell lines [34]. MRJP1 and MRJP2 stimulate mouse macrophages and the release of tumor necrosis factor α (TNF-α) [35], while MRJP 3 modulates the immune response by suppressing the production of interleukin (IL)-4, IL-2, and interferon γ (IFN-γ) in T-lymphocytes [36,37]. Yet another protein primarily identified in RJ is apolypophorin III-like protein, a lipid-associated protein that transfers lipids in aqueous environments in the form of a protein–lipid complex [38]. 

## 3. Royal Jelly as Nutrient for Queen and Larvae

RJ, a yellowish-white, creamy liquid, is a special food for queen bees and larvae in the hives. All larvae are fed with RJ, but only for the first three days. Afterwards, only those larvae that will become queens are fed with RJ, as well as adult queens [3,39,40,41,42]. The bee queens are approximately two times larger and live 10 times longer than worker bees. The difference in feeding, particularly during the larval stage, is considered as the main contributing factor to their exceptional fertility, morphology, lifespan, and behavior, including their memory abilities, compared to worker bees [39]. There are three types of royal jelly: worker jelly, royal (queen) jelly, and drone jelly. Royal jelly is white, acidic (pH 3.6–4.2), and of a specific flavor. Compared to worker and drone jelly, the Queens’ royal jelly contains 10 times more pantothenic acid, biopterine, and neobiopterine. During the development of the worker and drone larvae, the glucose-to-fructose ratio changes from 0.1 to 0.7, while in the queen’s royal jelly it remains constant, 1.2–2.5. Kamakura [43] has demonstrated that egg differentiation into queen or worker bees is determined by the intake of royal jelly; more precisely, royalactin (MRPJ1), a 57 kDa protein of RJ, promotes larvae differentiation into queens. Royalactin increases the size of the body and ovary size, and reduces the developmental time of queens. The effects of royalactin on body size are mediated by p70 S6 kinase, the increased activity of mitogen-activated protein kinase (MAPK), and a shortened developmental time, whereas an increased concentration of the juvenile hormone affects the development of ovaries. All these effects during the bee larvae differentiation into queens are triggered through the activation of the epidermal growth factor receptor (EGFR) signaling pathway [30,43,44].

During the spring, a queen that is well-mated and well-fed can lay app. 1500 eggs per day. At every moment, she is surrounded by worker bees who take care of her needs. They provide queen with the food and dispose of her waste. The queen bee has no direct control over the hive. She serves as the reproducer and is able to determine the sex of the eggs she lays, i.e., a fertilized (female) or unfertilized (male) egg, depending on the width of the cell. To fertilize the egg, the queen selectively releases the sperm stored in her spermatheca when the egg passes the oviduct [45]. In queenless honey bee colonies, bee workers that are fed with the RJ-rich diet may acquire queen-like properties and increased fertility (through ovarian development). In particular, the consumption of tyrosine in RJ increases brain levels of dopamine and tyramine and promotes the transition from normal to reproductive workers. The activation of EGFR also regulates this process by increasing the production of the juvenile growth-enhancing hormone (4–8 mM in RJ). Furthermore, RJ improves the memory and survival of bee workers due to its high concentration of acetylcholine. The permanent expression of the DNA methyltransferases 3 (*DNMT3*) gene which encodes DNA methyltransferase and has an important role in the formation of long-term memory further contributes to the extraordinary cognitive performances of queen bees [46,47].

During the queen honeybee and larvae development, MRJPs from the RJ are an important source of certain essential amino acids, whereas lipids are responsible for biological activities related to the development strategies of the colony [48]. Besides providing various nutrients, the ideal viscosity of RJ ensures that developing queen larvae are kept in place. This consistency of RJ is dependent on a protein–sterol complex made of two proteins and a sterol, MRJP1, apisimin, and 24-methylenecholesterol (24MC), respectively. The complex contains four MRJP1 molecules that surround four molecules of apisimin and eight molecules of 24MC. The viscosity of the complex is pH-dependent. A low pH increases the viscosity of RJ due to the formation of the fibrillar structures of the complex. Interestingly, proteins of the complex are produced by hypopharyngeal glands, whereas acidic conditions are achieved by mandibular glands that secrete fatty acids. The formation of fibrils also depends on the 10-HDA secreted from the mandibular glands. In queen-right colonies, workers predominantly produce 10-HDA and 10-HDAA by their mandibular glands, while the main components of the mandibular glands of the queen are (E)-9-oxodec2-enoic acid (9-ODA) and (E)-9-hydroxydec-2-enoic acid (9-HDA) that constitute a queen mandibular pheromone [49]. 10-HDA, a fatty acid, can account for up to 5% of RJ composition and may act as a histone deacetylase (HDAC) inhibitor. During the development and adult life of bees, there is a strong link between the queen–worker differentiation, OS, longevity, and dietary levels of metal cations, including zinc, iron, and potassium, based on the modulation of HDAC activity by these chemical species [50]. Among other possible mechanisms, a regulation of HDAC3 activity by metal cations and 10-HDA may represent possible epigenetic mechanisms of queen–worker bee differentiation. Therefore, various metals at different levels in RJ and worker bee jelly potentially may affect plasticity in caste differentiation and behavior through epigenetic regulation. Reducing sugars in RJ are also thought to contribute to the epigenetic effects by activating the insulin-like growth factor 1 (IGF-1) and mammalian target of rapamycin (mTOR) signaling cascades. Thus, they stimulate differentiation into queens through the increased intake of food and essential nutrients [51]. Moreover, as concentrations of various trace elements and minerals are found to be fairly constant in RJ samples of different botanical and geographical origin, it has been suggested that RJ, viewed as a form of insect lactation, shows homeostatic adjustments similar to mammalian milk.

## 4. RJ Values in Human Nutrition and as a Nutraceutical

The importance of RJ from the perspective of human nutrition is relatively small. Considering a daily intake of 2 g of RJ, the proteins, lipids, carbohydrates, and minerals present in RJ do not contribute significantly to the recommended daily intake (RDI). On the other hand, there is a small contribution of the vitamins B1, B2, B6, and biotin (B7). In addition, RJ is one of the best sources of pantothenic acid (vitamin B5) which is needed to release energy from food. B5 is the most abundant vitamin in RJ (52.8 mg/100 g), followed by niacin (B3) (42.42 mg/100 g). A B5 deficiency increases susceptibility to infections, bad mood, and gastrointestinal problems. All B-group vitamins help in the conversion of proteins, carbohydrates, and fats into energy. B vitamins are also important for healthy skin, hair, and eyes, the proper functioning of the nervous system and liver, a healthy digestive tract, the production and differentiation of all blood cells, especially erythrocytes, and the production of steroid hormones in the adrenal glands. Hence, the mentioned vitamins are important components that increase the nutritional value and the health-promoting potential of RJ.

As already mentioned, the concentrations of trace and mineral elements are quite constant in different RJ samples. In fresh RJ, the ash content is 0.8–3%, while in dry RJ it is 2–5%. The ash contains different minerals—K^+^, P^3−^, S^2−^, Na^+^, Ca^2+^, Al^3+^, Mg^2+^, Zn^2+^, Fe^2+^, Cu^+^, and Mn^2+^—as well as trace elements including Ni, Cr, Sn, W, Sb, Bi, and Ti. Trace elements and minerals play a key role in the biomedical activities associated with RJ, participating in various biological effects. The most abundant microelements in RJ are Zn, Fe, Cu, and Mn [3,11,52].

The presence of potassium as the main macroelement should be highlighted (321.1–357.4 mg/100 g,), followed by phosphorus (338.4–412.1 mg/100 g), sulfur (153.2–169.3 mg/100 g), calcium (22.8–24.0 mg/100 g), magnesium (44.0–50.4 mg/100 g), and sodium (0.3–13.8 mg/100 g) [52,53,54,55,56]. Potassium regulates the fluid balance, decreases the blood pressure, regulates the electrical activity of the muscle cells and heart, and improves the bone mineral density [53,54].

Ca is important for the bone mineral density and bone mineral content. The intake of calcium is usually insufficient at all ages which has adverse effects on bone health, weight gain, and fat accumulation. In contrast, diets rich in calcium can prevent fat accumulation, regulate blood pressure and premenstrual syndrome, and reduce the risk of colon cancer [54,55,56]. Mg also helps with high blood pressure, cardiovascular diseases, osteoporosis, and diabetes, whereas Zn supports growth, development, and immune functions. Iron participates in various metabolic processes, such as oxygen transport, DNA synthesis, and electron transport [54].

As previously emphasized, MRJP1 is the main protein from RJ which may exist in monomeric (55 kDa) and oligomeric forms. Many lines of evidence indicate that MRJP1 has a wide range of pharmaceutical effects on human health. It shows wound healing and antibacterial, antifungal, hypocholesterolemic, antitumor, and immune-enhancement activities [3,7,13,48,57]. In addition, MRJP 1–5 are important sources of 10 essential amino acids (Arg, His, Ile, Leu, Lys, Met, Phe, Thr, Trp, and Val), MRJP2, MRJP3, and MRJP5 provide the nitrogen supply, while MRJP 6–9 do not have a nutritional value. MRKP5 has the highest content of essential amino acids (51.4%), followed by MRJP1 (48%) and MRJP2 (47%) [3,57].

It has been mentioned that RJ contributes to the unique qualities of bee queens, including their excellent learning and memory abilities. In animal models of aging and Alzheimer’s disease (AD), RJ was able to enhance learning and memory retention, as well as prevent and treat cognitive deficits. In preclinical studies, in animal models and in cell lines, RJ, enzyme-treated RJ (eRJ), 10-HDA, RJ peptides, and MRJPs were effective against AD pathology by interfering with protein misfolding, amyloid synthesis, and amyloid clearance. Furthermore, RJ promoted neuronal survival and functioning by targeting inflammation, OS, mitochondrial dysfunction, disturbed proteostasis, amyloid β (Aβ) toxicity, Ca-mediated excitotoxicity, and bioenergetic failure. In clinical trials, RJ was effective against high blood pressure, diabetes, multiple sclerosis, infertility, menopausal symptoms, and even cancer [58,59].

Based on all these findings, [60] RJ has a great potential for the development of novel dietary supplements with valuable nutritional and bioactive properties. For example, the addition of RJ improved the probiotic viability and antioxidant, antimicrobial, and anticancer activities of fermented milk. When compared to the control fermented milk, milk supplemented with 1% of RJ preserved more *Lactobacillus helveticus* Lh-B02 during the 21 days of cold storage. *Lactobacillus helveticus* may improve the antioxidant status and immunological response of the host, produce bioactive peptides, and enhance the bioavailability of the nutrients’ production of biopeptides. Fermented milks with 0.5%, 1%, and 1.5% RJ have shown better radical scavenging activity (30.15%, 45.13%, and 58.36%, respectively) compared to fermented milk (27.62%). Fermented milk with 1.5% RJ demonstrated the best anticancer and antibacterial properties, but milk with 1% RJ had the best sensory acceptability (in terms of flavor, appearance, and color). Increasing the RJ content from 1.0% to 1.5% significantly improved the antimicrobial activities against *Staphylococcus aureus* ATCC 25923, *Candida albicans* ATCC 10231, *Aspergillus niger* NRRL 326, and *Aspergillus flavus* NRRL 1957. All fermented milks containing RJ (0.5–1.5%) have shown anticancer activity and the inhibited growth of various cancer cell lines, such as MCF-7, HepG2, HCT-116, and MCF7-12F, perhaps due to the presence of 10-HDA. The authors suggested that probiotic fermented milk with 1.5% RJ could be a promising option for developing novel functional symbiotic milk with health-promoting effects [60,61].

It is worth mentioning the numerous polyphenolic components of RJ, along with various glycosides, which have huge potential to contribute to human health [62,63,64,65,66,67]. However, many of these claims still need scientific evidence from clinical trials. The most important bioactive components of RJ and their functional roles are shown in Table 2.

## 5. Biological Function of Royal Jelly

The biological role of RJ as a functional food refers to its bioactive components and their antioxidant and anti-inflammatory properties, its effects on beauty and aging delay, immunity, memory, chronic inflammatory diseases, wound healing, diabetes, obesity, hypertension, osteoporosis, reproduction, antimicrobial properties, anti-allergic/allergic properties, as well as antitumor properties and protection against the accompanying toxic effects of chemotherapy and radiation. The biological and pharmacological effects of RJ have been shown in cell cultures, animal models, and human experiments.

### 5.1. Antioxidant and Anti-Inflammatory Activity of RJ

The most important antioxidants in RJ are flavonoids and phenolic compounds. As determined by the study of Nabas et al. [62], RJ contains 23.3 ± 0.92 gallic acid, equivalent (GAE) μg/mg of the total phenolic components, and 1.28 ± 0.09 rutin equivalent (RE), μg/mg of the total flavonoids. The flavonoids of RJ are an important group of phenolic compounds with a pronounced ability to scavenge free radicals. They can be differentiated into four groups: (i) flavanones, e.g., pinocembrin, hesperetin, isosakuranetin, and naringenin; (ii) flavones, e.g., acacetin, apigenin and its glucoside, chrysin, and luteolin glucoside; (iii) flavonols, e.g., quercetin, galangin, fisetin, isorhamnetin, and kaempferol glucosides; and (iv) isoflavonoids, e.g., coumestrol, formononetin, and genistein [62,63]. Fatty acids and their esters, including octanoic acid, benzoic acid, 2-hexenedioic acid and its esters, and dodecanoic acid and its ester, known as 1,2-benzenedicarboxylic acid, contribute to the antioxidant capacity of RJ as well [7,13,64,65]. In addition, the antioxidant activity of RJ may be related to the presence of free amino acids [63,65]. Furthermore, the antioxidant capacity and the total amount of polyphenols are dependent on the time of the collection of RJ; the best antioxidant properties were detected after 24 h [62,63].

The antioxidant abilities of RJ were investigated by various methodological approaches, including the scavenging of 1,1-diphenyl-2-picrylhydrazyl (DPPH), hydroxyl and superoxide radicals, reducing power, and the inhibitory effect on superoxide dismutase activity and linoleic acid oxidation. The antioxidant abilities of RJ were also dependent on the larval age and time of collection. Depending on the time of harvest (24–72 h after the larval transfer), the DPPH radical scavenging ability was between 43.0 and 62.8%, the inhibition of the superoxide radicals was in the range 23.9 to 37.4%, those of hydroxyl radicals varied from 48 to 68%, and linoleic acid oxidation was reduced by 8.6–27.9%. Furthermore, RJ harvested 24 h after the larval transfer has shown the highest reducing power, while the most significant effect on SOD activity was observed for RJ collected 72 h after transfer. These findings indicate that superoxide radicals’ scavenging and SOD inhibition are likely mediated by different compounds from RJ. The antioxidant properties of RJ have also been demonstrated in vivo. Mice that were fed RJ had reduced levels of 8-hydroxy-2-deoxyguanosine (8-OHdG), a marker of oxidative DNA damage, in the kidneys and serum [62,64,65].

Interestingly, di- and tri-peptides obtained by the hydrolysis of RJ proteins have also demonstrated the antioxidant properties, especially regarding hydrogen peroxide-scavenging. However, the metal-chelating activity and scavenging of superoxide radicals were not observed. From 29 hydrolyzed peptides, 12 peptides with 2–4 residues have demonstrated prominent antioxidative properties, of which the dipeptides Lys-Tyr, Arg-Tyr, and Tyr-Tyr have shown strong scavenging activity through the donation of a hydrogen atom of the phenolic hydroxyl group of Tyr residues [64,65]. Similarly, MRJPs have shown a DPPH radical-scavenging ability and have protected DNA against oxidative injury [29,66]. In another study, recombinant MRJPs from *Apis cerana* (AcMRJPs) protected NIH 3T3 cells from OS-induced apoptosis by reducing caspase-3 activity. AcMRJPs also demonstrated an antioxidative effect and protected DNA from oxidative damage in an in vitro assay [66].

**Figure 1:**
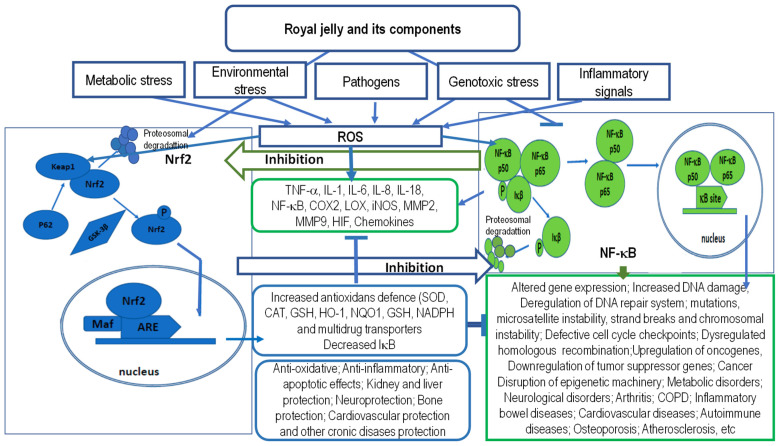
Modulation of Nrf2 and NF-κB signaling pathways by royal jelly (RJ). RJ reduces reactive oxygen species (ROS)-induced tissue damage and toxicity by modulating Nrf2 and NF-κB signaling pathways. Nrf2 and NF-κB are important regulators of the body’s response to oxidative stress and inflammation. The figure shows potential molecular mechanisms of RJ action via Nrf2 and NF-κB pathways and important effects on harmful exogenous and endogenous factors, as well as interplay between Nrf2 and NF-κB signaling. NF-κB functions as a homo- or heterodimer derived from one or more of the five members of the NF-κB family (RelA/p65, RelB, cRel, NF-κB1 or p50, and NF-κB2 or p52), and is activated by stimulus-dependent inhibitor degradation, post-translational modifications, nuclear translocation, and chromatin-binding. The activated NF-κB drives the pro-inflammatory response that plays an important role in the pathogenesis of chronic inflammatory diseases. In the nucleus, p65 coordinates gene transcription by recruiting coactivators (e.g., CREB-binding proteins (CBP)) or corepressors (e.g., histone deacetylases (HDACs)). Nrf2 and NF-κB compete for the CBP-binding in the nucleus; which transcription factor will bind to CBP depends on the relative amounts of translocated Nrf2 and NF-κB. In addition, NF-κB recruits HDAC3 which deacetylates Nrf2, reduces Nrf2 levels, and inhibits expression of ARE-dependent genes.

Furthermore, the antioxidative effects of RJ have been demonstrated in liver and kidney tissues exposed to various harmful stimuli with pro-oxidant activity. RJ reduced the extent of lipid peroxidation and improved the activities of the key antioxidative enzymes glutathione reductase (GR), glutathione peroxidase (GPx), superoxide dismutase (SOD), and catalase (CAT). The activation of the transcription factor nuclear factor erythroid 2-related factor 2 (Nrf2) has been recognized as one of the most important mechanisms underlying the antioxidative activity of RJ. Nrf2 regulates cellular redox balance and offers protection against toxic and oxidative insults by controlling the expression of genes involved in the oxidative stress response and drug detoxification [67]. In particular, its transcriptional activation improves its resistance to oxidative stress, chemical carcinogens, and inflammatory challenges. Chi et al. [67] demonstrated that RJ induces the transcriptional expression of Nrf2 and its target genes heme oxygenase (HO-1) and GPx1, suggesting that RJ activates the Nrf2–HO-1 pathway to improve antioxidant defense. Moreover, changes in the gene expression profiles were paralleled with the improved activity of enzymatic antioxidants in the liver. It has been suggested that the phenolic components of RJ are likely responsible for the observed induction of Nrf2 expression at the gene level (Figure 1).

The inflammatory response is also characterized by the orchestrated activation of intracellular signaling pathways that regulate the expression of various pro- and anti-inflammatory mediators. The activation of the transcription factor NF-κB is widely implicated in various inflammatory diseases. NF-κB regulates the expression of proinflammatory molecules, including cytokines, chemokines, and adhesion molecules. Consequently, the activation of the canonical NF-κB pathway largely contributes to the pathogenesis of chronic inflammatory diseases such as atherosclerosis, asthma, chronic obstructive pulmonary disease (COPD), inflammatory bowel disease (IBD), multiple sclerosis (MS), rheumatoid arthritis (RA), ulcerative colitis, and others. On the other hand, RJ is able to modulate levels of the main inflammatory mediators, including the aforementioned cytokines, chemokines, and adhesion molecules, thereby attenuating the inflammatory response. The anti-inflammatory abilities of RJ are mediated by various mechanisms. Besides inhibiting the production of pro-inflammatory cytokines, RJ also suppresses the NF-κB signaling pathway and modulates immune cells’ functions (see Figure 1).

Regarding individual compounds, the anti-inflammatory effects of RJ are attributed to 10-HDA, 10-HDAA, and sebacic acid (SA). The anti-inflammatory effect of 10-HDA, the main lipid component of RA, has been demonstrated by many studies. Thus, Jang and colleagues [68] have investigated the effects of 10-HDA on the production of TNF-α, IL-1β, IL-8, and IL-1ra in WiDr cells. 10-HDA inhibited the production of TNF-α, IL-1β, IL-8, and NF-κB but increased the amount of IL-1ra. The inhibitory effects (81.79%) on the production of TNF-α were observed at a 3 mM concentration of 10-HDA. Thus, the results indicate that 10-HDA has the ability to suppress the secretion of pro-inflammatory cytokines and increase the production of anti-inflammatory cytokines. Enzyme-treated RJ (ERJ) has also shown the anti-inflammatory and immune-promoting properties. ERJ decreased the levels of TNF-α, IL-1, IL-6, IL-10, IFN-γ, and IL-12, and increased the proliferation of B- and T-lymphocytes and natural killer cells (NK-cells). In another study, 10-HDA greatly reduced the expression of pro-inflammatory cytokines (IL-1, IL-6, cyclooxygenase 2 (COX-2), and monocyte chemoattractant protein-1 (MCP-1)) and the activation of the NF-κB and c-Jun N-terminal kinase (JNK) pathways. Furthermore, 10-HDA can act as an HDAC inhibitor and has the ability to inhibit the proliferation of fibroblast-like synoviocyte (FLS) cells induced by RA, a systemic chronic inflammatory disease [13,69]. The anti-inflammatory effects of MRJP3 or its C-terminal tandem pentapeptide repeats (TPRs) were also reported. They inhibited the expression of TNF-α and IL-6 mRNAs in lipopolysaccharide (LPS)-stimulated THP-1 cells. The anti-inflammatory activity of MRJP3 and its ability to suppress the production of the pro-inflammatory cytokines has also been observed in mice [36,69,70,71].

By activating the Nrf2 pathway, RJ increases the antioxidant capacity and inhibits the inflammatory response mediated by NF-κB, reducing the organ toxicity induced by endogenous and exogenous factors. RJ directly or indirectly activates Nrf2 signaling, prevents IκBα degradation, increases expressions of HO-1, and inhibits the activation of NF-κB. In the nucleus, Nrf2 heterodimerizes with small Maf proteins (MafF, MafG, and MafK) and binds to the antioxidant responsive element (ARE). Through the activation of the Nrf2 pathway, RJ increases the cellular antioxidant defense, promoting the resistance to the toxic effects of various stressors and drugs. In addition, RJ may increase the activation of the Nrf2 pathway and ARE gene transcription by increasing the free CBP levels, and by reducing the recruitment of HDAC3 to the ARE region. Furthermore, RJ may inhibit the nuclear translocation of NF-κB. 

Note: ARE, antioxidant responsive element; CAT, catalase; CBP, CREB-binding proteins; HDACs, histone deacetylases; COPD, chronic obstructive pulmonary disease; COX-2, Cyclooxygenase-2; GSH, glutathione; GSK-3β; glycogen synthase kinase-3β; HIF, hypoxia-inducible factor; HO-1, heme oxygenase 1; IκB, Nuclear factor of kappa light polypeptide gene enhancer in B-cells inhibitor; iNOS, inducible nitric oxide synthase; Keap1, kelch-like ECH-associated protein 1; LOX, Lipooxygenase; Maf, Maf proteins (MafF, MafG, and MafK); MMP-9, matrix metalloproteinase 9; NADPH, nicotinamide adenine dinucleotide phosphate; NF-κB, nuclear factor kappa-light-chain-enhancer of activated B cells; NF-κB1 (also named p50), NF-κB2 (also named p52), *NQO1*, NAD(P)H quinone oxidoreductase 1; Nrf2, nuclear factor-erythroid 2-related factor 2; RelA (p65), ROS, reactive oxygen species; SOD, superoxide dismutase; and TNF-α, tumor necrosis factor alpha.

Ulcerative colitis (UC) and Crohn’s disease (CD) are inflammatory bowel diseases (IBD) with a complex etiology resulting from interactions of genetic, environmental, and microbial factors.

The effects of RJ have been investigated in several animal models of colitis. Colitis is induced by either 2, 4, 6-trinitrobenzene sulfonic acid (TNBS), dextran sulfate sodium (DSS), or acetic acid (AA). RJ exhibited anti-inflammatory, antioxidant, and regenerative effects and improved the colonic mucosal barrier and intestinal gut microbiota. In rats with AA-induced colitis, treatment with RJ alleviated the damage of the colon tissue and the number of colonic CD3+CD45+ T cells and mast cells, without affecting the number of CD68+ macrophages and CD5+T cells. RJ also stimulated the production of the anti-inflammatory cytokine IL-10 and GPx activity. In rats with TNBS-induced colitis, supplementation with RJ also reduced CD3+, CD5+, CD8+, and CD45+ T cells, the production of pro-inflammatory cytokines IL-1β and TNF-α, and the levels of COX-2 and NF-κB. In the DSS-induced mouse model of colitis, treatment with RJ (2.0 g/kg) improved symptoms, reduced the apoptosis of colonic cells, and decreased intestinal permeability by upregulating the expression of the tight-junction protein, goblet cells, and their product, glycoprotein mucin 2 (MUC2). RJ also reduced the expression of proinflammatory cytokine IL-6 and increased the levels of anti-inflammatory cytokine IL-10 and antibody sIgA, which has an important role in mucosal immunity. RJ also improved DSS-induced changes in the relative abundance of various bacterial strains in the colon, including *Parabacteroides*, *Proteobacteria* (*Gammaproteobacteria*, *Enterobacteriales*, and *Enterobacteriaceae*), *Escherichia Shigella*, and *Muribaculum*. These results indicate that RJ might reduce colon damage by reducing intestinal inflammation [72]. In another study, in mice fed with RJ, RJ increased the relative abundance of *Lachnospiraceae_NK4A136_group*, *Prevotellaceae*, and *Bacteroides* and decreased the relative abundance of Alistipes [73].

All-together, increasing evidence suggests that RJ not only stimulates immunity and antioxidative activities, but also regulates the composition and structure of the gut microbiota. The gut barrier has an important role in transporting nutrients and metabolites across the epithelial cells and prevents damage by intraluminal substances [68,72,74].

It has been shown that RJ proteins may regulate immune function by stimulating macrophage activity and by reducing the levels of inflammatory mediators [75]. Thus, in immunodeficient cyclophosphamide-treated mice, the monomeric MRJP1, MRJP2, and MRJP3 proteins increased the number of murine macrophages, stimulated the immune response, and improved the composition of intestinal flora [76]. In particular, MRJPs affected the development of the spleen and liver, the number of leukocytes in the peripheral blood, the immunoglobulin content, cytokine levels, and the proliferation ability of spleen T- and B-lymphocytes.

In another study performed in *db*/*db* mice, RJ improved gut microbiota dysbiosis and intestinal and liver inflammation. It also regulated the expression of genes involved in nutrient absorption in the small intestine, increasing the excretion of saturated fatty acids in feces and the number of short-chain fatty acid (SCFA)-producing intestinal bacteria, altogether improving the atrophy of the intestinal mucosa. In general, SCFAs are end-products of the fermentation of non-digestible carbohydrates by gut microbiota. They are the main nutritional source for epithelial cells and are accordingly important for colon health. In addition to increasing mucus production and strengthening the tight junctions between the intestinal epithelial cells, SCFAs also cause an increase in mucin expression in goblet cells. Furthermore, SCFAs bind to the free fatty acid receptor and inhibit the activation of NF-κB [77], suppressing the secretion of pro-inflammatory cytokines like IL-1, IL-12, and TNF-α [78,79,80]. These cytokines further impact innate lymphoid cells (ILC1 and ILC3) and the production of their cytokines, including NF-α, IFN-γ, IL-17, and IL-22, leading to inflammation and cytotoxicity.

According to Kobayashy et al. [80], RJ can help in non-alcoholic fatty liver disease (NAFLD). Besides improving gut dysbiosis and inflammation, RJ increases the excretion of saturated palmitic acid in the feces, thus decreasing its content in the blood and liver, and increases the content MCFAs in the liver. In RJ, four main MCFAs have been identified: 10- HDA, 10 HDAA, 2-decenedioic acid (2-DA), and sebacic acid (SA). This finding is important as it is recognized that a high content of saturated fatty acids in a diet may increase the secretion of IL-12 from macrophages, induce mitochondrial damage, and sustain chronic low-grade inflammation, contributing to the pathogenesis of metabolic disorders, muscular atrophy, progressive liver inflammation, and cardiovascular diseases [77,78,79,80]. NAFLD pathogenesis is related to the “two-hits” hypothesis, which suggests that steatosis develops into nonalcoholic steatohepatitis (NASH) through excessive free fatty acids (FFAs), hyperinsulinemia, and lipid peroxidation [81]. The disturbance of the circadian rhythm and its Period genes (*Pers*) is associated with disruption of metabolic activity, increased fat storage, and a decrease in its consumption, ultimately causing hepatic steatosis which exacerbates the development of NAFLD [82]. According to You et al. [82], RJ could decrease the body and liver weights, improve cholesterol levels, downregulate ALT and AST levels, and combat NAFLD in OVX rats. The preventive effect of RJ may align with its strong anti-oxidative activity and its capacity to ameliorate the disturbances of the circadian genes, *Per1* and *Per2*. In another study, the protective effect of RJ was demonstrated against NAFLD due to its antioxidant and anti-inflammatory effect, as well as regulating the metabolism of FAs such as α-linolenic acid, linoleic acid, and arachidonic acid, and the biosynthesis of unsaturated fatty acids [83]. Felemban and coauthors [84] revealed that the chronic feeding of RJ to HFD-fed rats not only reduced the gain in weight and improved insulin resistance (IR), but also attenuated hyperglycemia and alleviated hepatic damage and steatosis. In addition, this study showed that the antidiabetic and anti-steatosis mechanisms by which RJ acts involve at least antioxidant potential, as well as the activation of the hepatic AMPK signaling-mediated upregulation of PPARα (fatty acid oxidation) and the suppression of SREBP1/2 (de novo lipogenesis) The treatment with RJ also reduced the serum levels of liver function enzymes, interleukin 6 (IL-6), tumor necrosis factor-α (TNF-α), and leptin, but significantly increased the serum levels of adiponectin. In addition, 10-HDA inhibits matrix metalloproteinases (MMPs) which degrade matrix and non-matrix proteins, resulting in tissue aging (e.g., skin) and chronic inflammatory diseases such as rheumatoid arthritis (RA) [85].

RA is an autoimmune inflammatory disease with an unclear etiology. The hallmarks of RA are hyperplasia and persistent inflammation of the synovial membranes, which penetrate deep into the bone and articular cartilage. The damage of bone and cartilage in RA joints is attributed to MMPs produced by the rheumatoid arthritis synovial fibroblasts (RASFs). MMP-1 (collagenase 1) and MMP-3 (stromelysin 1) are particularly important proteases in RA-associated tissue degradation. TNF-α is one of the well-known cytokines that stimulates the production of MMPs by activating the MAPK signaling pathways. The regulation of MMPs’ production is achieved through the activation of JNK, p38, and NF-κB in synovial tissue and cultured RASFs. It has been shown that 10-HDA from the RJ may reduce joint destruction in RA. 10-HDA exerted its effects by reducing the TNF-α-induced gene expression of MMP-1 and MMP-3, and by inhibiting the activation of the p38 and JNK/AP-1 signaling pathways. On the contrary, it had no effect on the TNF-α-induced activation of the extracellular signal-regulated kinase (ERK) cascade, NF-κB activity, or IκBα degradation [85]. These findings also indicate that the inhibition of the p38 and JNK pathways could be a possible therapeutic target against inflammation-related joints’ destruction in RA [85].

Overall, the anti-inflammatory effects of SA, 10-had, and 10-HDAA are not related only to the regulation of proteins involved in MAPK and NF-κB signaling [86,87]. They also may affect estrogen signaling by stimulating the activity of estrogen receptors (ER), ERα and ERβ [35], potentially exerting beneficial effects on the bones, muscles, and adipose tissue in a sex-dependent manner. Another study demonstrated that the inhibitory effect of 10-HDA on the proliferation of fibroblast-like synoviocytes was mediated by its ability to act as an HDAC inhibitor and downregulate the PI3K–AKT pathway. It also increased the expression of genes involved in cytokine–cytokine receptor interactions [88].

Multiple sclerosis (MS), also a chronic inflammatory disease, affects about 250,000 to 350,000 individuals in the USA and markedly reduces their quality of life. Demyelinating lesions in the brain, spinal cord, and optic nerve are typical hallmarks of MS. Oshvandi et al. [89] showed that supplementation with RJ can be beneficial in improving quality of life; following a 90-day period of supplementation, the patients’ activity was increased. In another study, Seyyedi et al. [90] have reported that the consumption of RJ, through its estrogenic effect and reduced vaginal atrophy, improves the quality of life of postmenopausal women [90,91]. The anti-inflammatory effect of RJ was also confirmed in experimental autoimmune encephalomyelitis (EAE), an animal model of MS. RJ and 10-HDA reduced demyelination, regulated the polarization of Th17 and Th1 cells, and attenuated the infiltration of the peripheral immune cells into the CNS. In addition to RA and MS, RJ can be useful in other human autoimmune diseases such as Graves’ disease and lupus erythematosus by strengthening the immune system.

RJ, MRJP3, or its C-terminal TPRs sequence may exert anti-inflammatory effects by inhibiting the production of pro-inflammatory cytokines TNF-α, IL-1β, IL-2, IL-6, and IL-33. This has been shown in various conditions, including herpes stromal keratitis (HSK) caused by *Herpes simplex virus* type 1 (HSV-1), LPS-stimulated THP-1 cells, formalin-induced rat paw edema, and nanoparticle-induced inflammation of the skin, gastrointestinal and respiratory tract, and cardiovascular system [92,93].

Environmental and occupational exposure to heavy metals, such as Cd, has been tightly linked to the ROS-induced oxidative damage of proteins, lipids, carbohydrates, and DNA, which underlies the development and progression of pathological changes in many diseases. Exposure to Cd also induces apoptotic events and modulates the ratio of pro- and anti-apoptotic proteins. In addition, Cd results in massive inflammation through the excessive release of pro-inflammatory mediators, like IL-1β, TNF-α, and nitric oxide (NO) [94]. It has been shown that RJ markedly reduces Cd-induced oxidative damage in mouse testes via the Nrf2 pathway [95]. By increasing Nrf2 and homoxygenase-1, RJ also protected the lymphocytes, liver, kidney, and heart tissue against drug- or toxin-induced oxidative injury [96]. In 6-hydroxydopamine-induced cell death, Nrf2 translocation to the nucleus and the enhanced expression of antioxidant and detoxifying enzymes were induced by 4-hydroperoxy-2-decenoic acid ethyl ester (HPO-DAEE), a lipid component of RJ [97].

### 5.2. Royal Jelly, Beauty, and Postponement of Ageing

**Figure 2:**
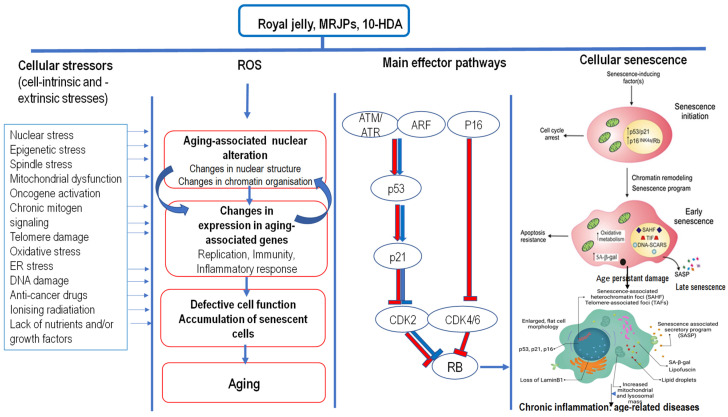
Anti-senescence effect and molecular mechanisms of royal jelly (RJ) and its components on cellular senescence, healthy aging, and longevity induced by endogenous and exogenous factors. Cellular senescence is triggered by various intrinsic and extrinsic stimuli such as ROS, inflammation, mitochondrial dysfunction, genotoxic stress, irradiation and chemotherapeutic agents, telomere shortening, irreversible DNA damage, or signals such as oncogene activation or the overexpression of pluripotency factors. These stressors initiate various cellular signaling cascades, ultimately resulting in the activation of p53, p16Ink4a, or both. Their activation induces cell cycle arrest by inhibiting cyclin D–Cdk4/6 and cyclin E–Cdk2 and prevents Rb inactivation, resulting in continuous repression of E2F target genes required for S-phase. ARF can inhibit MDM2 and stabilize p53, which leads to the arrest of the cell cycle and cellular aging and the possibility of repairing minor damages. Accordingly, senescence is associated with several molecular and phenotypic alterations, such as senescence-associated secretory phenotype (SASP), cell cycle arrest, DNA damage response (DDR), senescence-associated β-galactosidase, morphogenesis, and chromatin remodeling. The anti-senesce effects of RJ and its component (MRJPs, 10-had, and RJPs) is the result of the interplay between several genes involved in downregulation of insulin/insulin-like growth factor-1 signaling (IIS) and targeting of rapamycin (mTOR), upregulation of the epidermal growth factor (EGF) signaling, dietary restriction, and enhancement of antioxidative capacity via Nrf2 activation. These signaling pathways affect cellular processes associated with longevity: DNA repair, autophagy, antioxidant activity, anti-inflammatory activity, stress resistance, and cell proliferation. In addition, RJ suppresses cellular senescence by upregulation of SOD1 and downregulation of Mtor and catenin beta like-1, and by regulating the expression levels of p53, p16, and p21. The life-expanding effect of RJ possibly originates from its antioxidant and anti-inflammatory properties, which can promote healthy aging by improving glycemic status, lipid profiles, and oxidative stress and hence can prevent the occurrence of various debilitating metabolic diseases. Furthermore, it should be noted that RJ contains epigenetically active compounds that inhibit DNMT3 or HDAC3, thus changing the epigenetic information generated in response to exogenous and endogenous factors during the aging process. Note: ATM, Ataxia telangiectasia mutated; ATR, Ataxia telangiectasia mutated and Rad3 related; BDNF, brain-derived neurotrophic factor; CDK2, Cyclin-dependent kinase 2; CDK4/6, Cyclin-dependent kinase 4 and 6; ER stress, Endoplasmic reticulum stress; NGF, nerve growth factor; Nrf2, nuclear factor-erythroid 2-related factor 2; p21 and p16, cyclin dependent kinase (CDK) inhibitors; Rb, retinoblastoma protein; ROS, Reactive oxygen species; SA-β-gal, senescence-associated β-galactosidase; SAHF, Senescence-associated heterochromatin foci; SASP, Senescence-associated secretory phenotype; TAFs, telomere-associated foci.

The majority of the aging process is conditioned by the genetically programmed weakening and failure of the homeostatic mechanisms. Various stressors may induce the senescence process (see Figure 2), especially OS, mitochondrial dysfunction, ER stress, and epigenetic stress, leading to irreversible DNA damage and the activation of molecular mechanisms of aging associated with p53 regulation. The cell’s DNA damage response (DDR), particularly to double-strand breaks (DSB), results in p53 phosphorylation, p21 activation, and cell cycle arrest [98,99,100,101,102].

Continuous OS and chronic inflammation are associated with many cellular and molecular changes: damage of the main biological molecules, loss of enzymatic activity, mitochondrial dysfunction, reduced proteostasis, genome instability, weakened repair mechanisms, telomere shortening, epigenetic modifications, and the disturbance of gene expression and signaling pathways. In addition, these changes include the loss of intercellular communication, immunosenescence and dysfunction of effector functions of T and B cells, decrease in the antigen-presenting functions, exhaustion of stem cells, low-grade inflammation and increased cytokine release, dysbiosis of intestinal microbiota and alternations in intestinal permeability, and ultimately, cellular aging (see Figure 2).

Cellular senescence is induced by critically short telomeres, triggering the permanent activation of DDR and cell cycle arrest. The DDR is a well-known activator of the senescence-associated secretory phenotype (SASP) which is characterized by the increased expression of an array of pro-inflammatory factors, mostly in an NF-Κb-dependent manner, such as IL-6, IL-8, IL-1β, monocyte chemoattractant protein (MCP)-1, MCP-2 and MCP-4, growth factors like the human growth factor (HGF) and fibroblast growth factor (FGF), various proteases like MMPs, and secreted insoluble proteins/extracellular matrix proteins (ECM). Senescent cells disrupt the homeostasis and function of tissues and organs, resulting in the loss of tissue regeneration and the reduced function of stem cells. SASP components can induce tissue fibrosis in certain epithelial tissues, whereas the infiltration of macrophages and lymphocytes, fibrosis, and cell death are recognized as the cause of numerous diseases associated with aging [98,99,100,101,102].

Therefore, the antioxidant, anti-inflammatory, and antisenescence properties of RJ as well as the modulation of cellular senescence by targeting Nrf2 activation can be a promising preventive strategy against cancer and other degenerative diseases (NAFLD, neurodegenerative diseases, aging, heart diseases, and cardiovascular diseases) [58,100,101,102,103].

A daily intake of RJ can extend the lifespan of bees and other species, including nematodes, crickets, or mice. This effect is attributed to proteins and lipids from royal jelly and MRJPs. In *Drosophila melanogaster*, RJ extended longevity by promoting the epidermal growth factor (EGF) receptor-mediated pathway [104]. Royalactin (MRJP1) also stimulated EGF and epidermal growth factor receptor (EGFR) pathways in *Caenorhabditis elegans* [105]. Wang et al. [106] found that royal jelly and an enzyme-treated RJ (RJ/eRJ)-mediated lifespan extension requires insulin/IGF-1 signaling and the activities of the DAF-16, SIR-2.1, HCF-1, and FTT-2, a 14-3-3 proteins. These findings reveal that RJ/eRJ supplementation triggers a sophisticated regulatory mechanism, mediated by the interplays of DAF-16, SIR-2.1, HCF-1, and 14-3-3, to fine-tune DAF-16 activities and thereby promote prolongevity and stress responses in *C. elegans*. DAF-16 is a key regulator of stress responses; the DAF-16 target genes (sod-3, mtl-1, and F21F3.3) which promote *C. elegans’* response to oxidative stress, heavy metals, and heat shock. Thus, RJ and eRJ consumption increased the lifespan, health span, and the tolerance of *C. elegans* to oxidative stress, ultraviolet irradiation, and heat shock stress. Earlier studies reported that DAF-16/FOXO, SIR-2.1/SIRT1, FTT-2, HCF-1, and 14-3-3 proteins are evolutionarily conserved from *C. elegans* to mammals, and that these findings may have immediate implications in utilizing RJ/eRJ as aging-intervention means to prolong one’s health span and combat age-related diseases in higher-order organisms, including humans. In addition, 10-HDA can increase the lifespan of *Caenorhabditis elegans* through dietary restriction and the targeting of rapamycin (TOR) signaling [107]. Moreover, the long-term use of RJ in D-galactose-induced mice prevented age-related weight loss, improved memory abilities, and delayed thymus atrophy. It also reduced muscle cell atrophy and, consequently, increased the mobility and physical condition of mice [52,108,109,110]. In humans, the intake of RJ can also delay aging and the development of some age-related disorders, improve the quality of life during aging, and significantly delay age-related motor dysfunctions [52,110]. In human cell cultures, lipid components of RJ inhibited the aging process through the upregulation of EGF signaling and downregulation of insulin-like growth factors (IGF) [5,109,110,111]. In mouse embryonic stem cells, it was shown that royalactin can maintain pluripotency by activating a ground-state pluripotency-like gene network [44]. Furthermore, the same authors discovered Regina, a mammalian structural analog of royalactin that can promote ground-state pluripotency in mouse embryonic stem cells, indicating the possibility of using royalactin as a regenerative therapy.

Salazar-Olivio and Paz-Gonzales [112] also have shown that RJ proteins can have an effect in the postponement and prevention of ageing. In their study, RJ have promoted proliferation in insect cell cultures. Likewise, Watanabe et al. [33] have demonstrated the mitogenic effect of RJ on a human monocyte cell line (U-937 cells) and its ability to increase the synthesis of DNA and albumins in liver cells [113]. It is believed that RJ contains growth factors or hormones that stimulate cell growth. Taken together with the finding that certain protein fractions of RJ increase the synthesis of new adhesion molecules, this confirms the cosmetic efficacy of royal jelly [26,114]. Kawano et al. [114] demonstrated that RJ can reduce the expression of miR-129-5p and can prevent the photoaging of skin. It is possible that the effect of miR-129-5p a is not only involved in increasing the number of cells, but can prevent skin aging through the regulation of its target genes. Many genes are predicted as putative targets of miR-129-5p including calcium signaling-related molecules, such as the calcium channel, voltage-dependent, γ subunit 2 (CACNG), calcium/calmodulin-dependent protein kinase II inhibitor 1 (CAMK2N1), FK506-binding protein 2, 13 kDa (FKBP2), and calmodulin 1 (phosphorylase kinase, delta) (CALM1).

The skin is the largest organ that covers the entire surface of the body. The main function of the skin is to protect organism from environmental insults [115]. In order to fulfil its function, the skin can initiate a defense process targeted at tissue repair and pathogen removal [115]. However, an estrogen deficiency and decrease in collagen during menopause and natural aging reduce skin elasticity and strength. RJ constituents, especially 10-HDA, can reduce cell aging and stimulate the production of procollagen type I and transforming growth factor-1 (TGF-β1) [109,110,116,117,118,119,120,121]. Similarly, MRJP promotes cell proliferation, affects the telomere length, and reduces aging [109,111]. Furthermore, Koya-Miyata et al. [119] have shown a beneficial effect of RJ on collagen production in skin fibroblasts and an isolated active substance named the collagen production-promoting factor. The pharmaceutically active ingredients of RJ in collagen production were 10H-2DA and 10-HDA. These acids in the presence of ascorbic acid promote the production of TGF that further increases collagen production. It still remains unknown if these acids influence enzymes involved in collagen synthesis as well (prolyl hydroxylase and lysyl hydroxylase).

RJ is appreciated today as a protective agent against skin aging. Experiments performed in ovariectomized rats have indicated its ability to increase collagen production [117,120]. Following the administration of 1% RJ to female Sprague–Dawley rats without estrogen, the skin level of the type I procollagen protein was returned almost to normal values. Hence, according to the data provided, it seems that RJ could be an effective dietary supplement against the natural aging process of postmenopausal skin, but further clinical research is needed to confirm this [120].

Of note, collagen is a predominant fibrous protein of extracellular matrix and the main protein in connective human tissue, making about 3–6% of total body proteins. The functional properties of skin are largely dependent on the integrity of dermal collagen. Alterations in the level of collagen settling are observed during wound healing, bone development, and ageing. Hence, collagen metabolism control is important not only from a cosmetic perspective, but also for therapeutic applications.

In a recent study, Khani-Eshratabadi et al. [121] have shown the potential anti-apoptotic effects on RJ in different rat tissues (brain, liver, kidney, and lymphocytes) by regulating the levels of Bax, Bcl-2, and telomerase enzymes. In rats treated with RJ, there was a trend towards a telomerase increase, the significant effect on the reduction of the Bax/Bcl-2 ratio, and the improved survival rate of the liver, kidney, and especially brain cells at a dose of 300 mg/kg. A similar effect was obtained in a study conducted by Jiang et al. [111]. The treatment of a human embryonic lung fibroblast cell line (HFL-I) with different concentrations of MRJPS (0.1–0.3 mg/mL) had beneficial impacts on proliferation, senescence, and the telomere length. The antisenescence effects of MRJPs were associated with the upregulation of SOD1 and downregulation of the mTOR, catenin beta like-1 (CTNNB1), and transcription factor 53 pathways, which altogether had a stimulatory effect on DNA and protein synthesis. Likewise, in human lymphocytes exposed to the genotoxic chemotherapeutic compound doxorubicin, the beneficial effects of RJ were associated with the increased hTERT/BAX ratio, indicative of greater longevity [122].

Immunity is also affected by aging; aging reduces cell proliferation, the production of new naive lymphocytes, and the production of cytokines in the defense process, especially interleukin 2 (IL-2) [101,102]. Immunosenescence is a process associated with aging that leads to the dysregulation of cells of innate and adaptive immunity and the reduced ability of the immune system to respond to foreign antigens and maintain tolerance to self-antigens. Immunosenescence is characterized by a reduced proliferation capacity, telomere shortening, and increased resistance to the apoptosis of CD28+ T-lymphocytes in general [101,102,123]. In the elderly, the repertoire of naive CD4+ and CD8+T cells along with cytokine production is severely affected with alterations in memory B cells and antibody production [101,102,123], which results in high susceptibility to infection, cancer, and autoimmune diseases, and a reduced response to vaccinations. Bouamama et al. (124) demonstrated some mechanisms that delay PBMCs’ cell senescence in vitro by RJ through enhancing their cell proliferating ability, MDA, GSH, IL-2, IL-6, IL-4 cytokines, and NO release. The immuno-enhancing potential and anti-immunosenescence effects of royal jelly may play a primordial role in the health of the elderly to corroborate and combat pathological aging by improving the immune functions, enhancing PBMCs’ proliferation along with the cytokine release [123,124,125].

Inoue and co-authors [126] have shown that RJ can increase the lifespan of C3H/HeJ mice by reducing oxidative damage. The theory of «free radicals and ageing» is widely known; ROS production results in the overall damage of the cell [4,127,128] and its consequences include the modifications of cell proteins, lipids, and DNA. The results of many studies indicate the important contribution of the increased oxidative DNA damage to the ageing of the cell and organism. In fact, it is considered that DNA damage is the cause of ageing and degenerative diseases related to ageing, including tumors, heart diseases, and cardiovascular diseases (Figure 2). Mutations, a consequences of oxidative damage, shorten one’s lifespan. In the aforementioned study on C3H/HeJ mice, dietary supplementation with RJ increased mice survival by 50%. The authors found that the mechanism of protection was not based on the free radical scavenging ability of RJ, but on the suppression of oxidative DNA damage, perhaps through reduced chronic inflammation [103,104,105,126,129,130,131].

In addition, the accumulation of senescent cells in the tumor microenvironment can drive tumorigenesis in a paracrine manner through the SASP. Hatson et al. [132] suggested that senescent macrophages play an important role in the initiation and progression of lung cancer, and may be a potential target in cancer prevention strategies. It seems that the clearance of senescent macrophages can ameliorate tumorigenesis in KRAS-driven lung cancer. It has been suggested that SASP can reinforce senescence-induced cell cycle arrest by autocrine or paracrine mechanisms and can modulate the tissue microenvironment by paracrine pathways. For example, 16INK4a-expressing macrophages express many pro-tumorigenic SASP factors unique to a tumorigenic lung (i.e., Bmp2, CCL2, CCL7, CCL8, CCL24, CXCL13, and IL-10) and may represent important mediators of paracrine pro-tumorigenic effects. Chemokines CCL7, CCL24, and CXCL13, as well as IL-10 and Bmp2, exert pro-tumorigenic effects in a variety of cancers, promoting the invasiveness, metastasis, and stemness of cancer cells. Furthermore, the senescent cells may contribute to the formation of an immunosuppressive tumor microenvironment (TME) (rich in Tregs and low in CD4+ and CD8 + T cells), which can be switched toward an immunostimulatory TME (low in Tregs and high CD4+ and CD8 + T cells) through the clearance of senescent cells. On the other hand, macrophage depletion or a colony stimulating factor 1 receptor (CSF1R) signaling blockade disturb the intratumoral vascular network and reduce food and oxygen delivery to the tumor cells [98,132,133,134]. RJ compounds display their anticancer effects through a number of mechanisms such as controlling reactive oxygen species (ROS)-scavenging enzyme activities, suppressing the cell cycle, and promoting the apoptosis and inhibition of the proliferation of cancer cells, as well as their antimetastasis, antiangiogenic, etc., effects. The antioxidant, anti-inflammatory, anti-stress, immuno-enhancing potential, and anti-immunosenesence effects of RJ as well as the modulation of cellular senescence by Nrf2 activation can be a good preventive strategy in preventing the occurrence of cancer and other degenerative diseases.

### 5.3. Royal Jelly and Its Effect on Brain Cells

The ongoing rise in the elderly, particularly in Western countries, is one of the major worldwide problems today. The decline of cognitive abilities and increase in neuropsychiatric disorders, such as Alzheimer disease (AD) and depression, are closely related to aging. AD is a neurodegenerative disease characterized by intracellular aggregates of neurofibrillary tangles (NFTs) made of hyperphosphorylated and truncated tau proteins, and the extracellular deposition of amyloid beta (Aβ) peptides generated through the proteolytic cleavage of the amyloid precursor protein (APP) [135,136]. The accumulation of Aβ plaques activates microglial cells and the immune response, leading to increased OS, elevated intracellular Ca^2+^ levels, and a local inflammatory reaction, contributing to neurotoxicity. On the other hand, due to the selective loss of cholinergic neurons, acetylcholine (Ach), a neurotransmitter with a critical role in learning and memory, is depleted in AD patients. Levels of brain-derived neurotrophic factor (BDNF) are also reduced in patients with severe AD. BDNF is involved in learning and memory due to its important role in neuronal plasticity. In addition, BDNF inhibits cytokine production through its direct anti-inflammatory effect on microglial cells [137].

**Figure 3:**
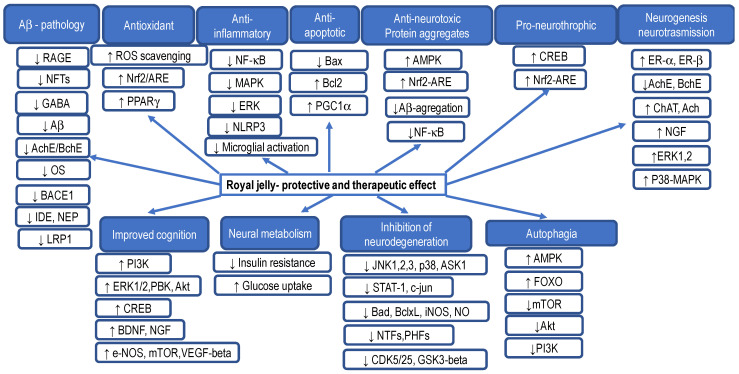
The possible protective and therapeutic mechanisms of royal jelly and its components in neuroprotection, cognitive performance, and suppression of neurodegeneration. Note: Aβ, amyloid beta; ACh, acetylcholine; AChE, acetylcholinesterase; AMPK, AMP-activated protein kinase; ARE, antioxidant responsive element; BACE1, beta-site APP cleaving enzyme 1; Bax, Bcl-2-associated X protein; BChE, butyrylcholinesterase; Bcl-2, B-cell lymphoma-2; BDNF, brain-derived neurotrophic factor; ChAT, choline acetyltransferase; CREB, cAMP-response element (CRE)-binding protein; ER β and α, estrogen receptors β and α; ERK, extracellular signal-regulated kinase; ERK1/2, extracellular signal-regulated kinase 1 or 2; eNOS, endothelial nitric oxide synthase; FOXO, forkhead box O transcription factor; GABA, gamma-aminobutyric acid; IDE, insulin-degrading enzyme; JNK, c-jun N-terminal kinase; LRP-1, low-density lipoprotein receptor-related protein; MAPK, mitogen-activated protein kinase; mTOR, mammalian target of rapamycin; NEP, neprilysin; NF-κB, nuclear factor-kappa B; NFT, neurofibrillary tangles; NGF, nerve growth factor; NLRP3, nucleotide-binding domain and leucine-rich repeat-containing protein 3; Nrf2, nuclear factor-erythroid 2-related factor 2; OS, oxidative stress; p38, p38 protein kinases; PI3K, phosphatidylinositol 3-kinase; PGC-1α, peroxisome proliferator-activated receptor-γ coactivator 1-a; PPAR-γ, peroxisome proliferator-activated receptor; RAGE, receptor for advanced glycation end-products; VEGF, vascular endothelial growth factor; ↑ upregulated; ↓ downregulated.

According to papers [58,138,139,140,141,142,143,144,145,146,147,148,149,150,151,152], RJ has many beneficial effects on cognition and AD pathology, including the improvement of memory, neuroprotection and regulation of neurotrophins, regulation of neurotransmission, regulation of brain energy metabolism, protection against oxidative stress and neuroinflammation, reduction of apoptosis, attenuation of Aβ-induced neurotoxicity, and improvement of hormonal and metabolic abnormalities associated with cognitive impairment (see Figure 3).

Many studies have highlighted the important role of neurotropic factors in the etiology and/or development of AD and other age-related diseases, suggesting that the increased synthesis of neuropeptide factors can be a promising avenue in the prevention of these diseases. Hashimoto et al. [153] have found that RJ selectively stimulates the mRNA expression of the glial cell line-derived neurotropic factor (GDNF) and neurofilament H, an axonal marker, in the hippocampus of adult mice. The neuroprotective effects of GDNF have been demonstrated in various conditions. Thus, the exogenous addition of GDNF protects the nigrostriatal dopaminergic terminals from neurodegeneration and ameliorated damage induced by cerebral ischemia and traumatic brain injury. These observations suggest that GDNF could be a promising approach in the protection and treatment of neurological diseases. However, further studies are needed of other RJ-induced active components with the effects on brain functions such as learning and memory. A better understanding of the biological effects of RJ at the molecular level likely will contribute towards more effective protection, as well as therapy for some neurological diseases, and to the more efficient use of RJ in healthcare.

The beneficial effects of RJ on cognition and AD-related pathologies have been demonstrated in various models, including copper- and cholesterol-fed rabbits [150], hippocampal neurons exposed to streptozotocin [151] and trimethyltin [154], and double transgenic APP/PS1 mice harboring mutations associated with early-onset AD (chimeric mouse/human APP (Mo/HuAPP695swe) and human presenilin 1 lacking exon 9 (PS1-dE9)) [145]. You et al. [145] argued that RJ may serve as a functional food for AD pathology. They found that the administration of RJ at a dose of 300 mg/kg/d for 3 months improved behavioral impairments and reduced the accumulation of Aβ in APP/PS1 mice. RJ also alleviated JNK-mediated apoptosis by suppressing oxidative stress. The authors suggested that RJ exerts protective effects on learning and memory by inhibiting or repairing AD-related hippocampal and cortical lesions. Importantly, the hippocampal levels of cAMP, p-PKA, p-CREB, and BDNF levels were significantly increased in RJ-treated mice, indicating the important effects of RJ on the cAMP/PKA/CREB/BDNF pathway in ameliorating cognitive decline. RJ also improved spatial learning and memory abilities in normally aged rats, as evidenced by the better performance in the Y-maze [139], Morris Water Maze test, passive avoidance test [103,151,154], and open field test [108].

Oxidative stress increases the amount of amyloid precursor protein (APP) and then further aggravates the AD pathology [145,155]. It is confirmed that oxidative stress is a therapeutic target of RJ in AD [138]. Some component derived from RJ as 10-HDA may be used to treat age-related neurodegenerative disorders; it stimulates neuronal differentiation from progenitor cells (PC12) through mimicking the effect of brain-derived neurotrophic factor (BDNF)] [156]. 10-had, in addition to stimulating neurogenesis via neural stem/progenitor cells, easily crossed the blood–brain barrier (BBB) and exerted unique neuromodulatory activity due to its smaller size [156]. In addition, it possesses neuroprotective effects against glutamate- and hypoxia-induced neurotoxicity [157]. Furthermore, AMP N1-oxide, the unique active nucleotide component present in RJ, has been shown to exhibit neurogenic and neurotrophic activities. AMP N1-oxide stimulates neurite outgrowth and induces the differentiation of PC12 cells into neurons through the activation of two signaling pathways: MAPK/extracellular signal-regulated kinase 1 or 2 (ERK1/2) and the phosphatidylinositol 3-kinase/Akt pathway. AMP N1-oxide promotes neurite outgrowth activity via adenylate cyclases coupled to adenosine A2A receptors crucial for neural development and the regulation of synaptic plasticity. It seems that AMP N1-oxide and 10-HDA are active components in RJ that reduce the deposition of Aβ by regulating the production, degradation, and clearance process, and in this way, they mitigate cognitive deficits and Aβ accumulation in the APP/PS1 mouse model [157].

A recent study by Aslan et al. [158] has shown that RJ (applied at a dose of 100 mg/kg bw during 56 days) prevented fluoride-induced brain damage via anti-oxidative effects. In addition, RJ reduced fluoride-induced increases in the expression of Bcl-2, NF-κB, and COX-2, and normalized the levels of caspase-3, caspase-6, Bax, and Erk.

Chen et al. [159] showed that supplementation with MRJPs improves the cognitive abilities of aged rats. MRJPs enhanced the metabolic activity of the brain by increasing the levels of glucose and phosphoenolpyruvic acid. The metabolomic analysis of the urine of MRJPs-fed rats did not show a significant difference compared to young rats. The main changes induced by MRJP supplementation were related to an increased level of nicotinic acid mononucleotide (NaMN), the precursor of NAD+, and xanthosine, which is important for the nucleic acid metabolism and DNA repair in old rats. MRJP proteins also increased the production of cysteic acid and other neuroprotective molecules in aged rats.

As previously mentioned, Aβ is produced from the APP through the amyloidogenic pathway. The first cleavage of APP is made by β-secretase (BACE1) which generates soluble APP peptide-β (sAPPβ) and C-terminal fragment-β (CTFβ). CTFβ is further cleaved by γ-secretase to yield hydrophobic Aβ polypeptides (predominantly Aβ40 and Aβ42). In N2a/APP695 neuroblastoma cells, crude royal jelly peptides (RJPs), obtained by the digestion of RJ proteins, reduced the production of Aβ40 and Aβ42 peptides by downregulating BACE1. Thus, RJPs could have the potential for ameliorating AD-related Aβ pathology [139].

Taken together, numerous studies have shown that RJ has an effect on various pathophysiological mechanisms related to cognitive decline and AD. RJ acts through neurotrophic, antioxidant, anti-inflammatory, antiapoptotic, and antiamyloidogenic mechanisms, delaying the onset of AD, slowing down its progression, and stimulating the recovery process.

Ali and Kunugi [59] summarized the main mechanisms of the RJ action contributing to the improvement of cognitive abilities and alleviation of Aβ pathology: (i) RJ reduces the influx of Aβ through the BBB by inhibiting the receptor for advanced glycation end-products (RAGE) which also acts as the receptor for Aβ, (ii) RJ prevents APP cleavage by inhibiting BACE1, and (iii) RJ facilitates the clearance of Aβ. In addition, predominantly by activating the AMPK signaling cascade, RJ stimulates autophagy and antioxidative defense, and suppresses inflammation. RJ and its lipid components also bind to estrogen receptors and increase the production of neurotrophic factors like NGF and BDNF and stimulate ACh production, neurogenesis, and synaptic plasticity.

Hattori et al. [154,156] have shown the effect of RJ and 10H2DA on the neurogenesis of neural progenitor/stem cells (NSCs) in vitro. NSCs have a self-regenerative ability and potential to differentiate into neurons, astrocytes, and oligodendrocytes. RJ stimulated the differentiation of all types of brain cells (neurons, astrocytes, and oligodendrocytes), whereas 10H2DA stimulated the production of neurons but reduced the differentiation of astrocytes. Hence, the RJ-induced activation of NSCs could be a relevant target for the treatment of AD and other neurodegenerative conditions.

### 5.4. Royal Jelly and Diabetes

RJ contains compounds functionally and structurally similar to insulin [160]. Moreover, as one of the most powerful therapeutic formulations with regenerative features, RJ may regenerate damaged pancreatic cells and prevent the development of diabetes [60,161,162,163,164,165]. The regeneration of pancreatic cells and preservation of insulin levels are key factors in lowering blood glucose [160,161,162,163,164]. In the Otsuka Long–Evans Tokushima Fatty (OLETF) rat model of type 2 diabetes, the long-term usage of RJ was effective in the prevention of insulin resistance [166]. Research by Zamami and colleagues also confirmed that RJ can reduce the index of insulin resistance (HOMA-IR), although not the blood glucose in rats [167]. The authors suggested that antioxidant peptides present in RJ are responsible for insulin resistance [162,167,168,169]. Overall, the beneficial effects of RJ observed in an animal model of diabetes include: (i) the improvement of the antioxidative properties of plasma; (ii) the reduction of biochemical parameters (alanine transaminase (ALT), aspartate aminotransferase (AST), and alkaline phosphatase (ALP)) and fasting blood glucose (FBG), and (iii) the reversal of histopathological changes in rat reproductive tissue (the tubular differentiation index, number of mononuclear immune cells, thickness of tunica albuginea, diameter of seminiferous tubules, Johnsen score, spermiogenesis index, Sertoli cell index, and meiotic index).

In healthy volunteers, RJ significantly reduced serum glucose levels 2 h after ingestion [170]. The same results are observed in diabetic patients [170,171]. In particular, RJ intake (3 g per day) reduced serum glucose, increased serum apolipoprotein (Apo)A-I, and modified the ApoB/ApoA-I ratio following 8 weeks of supplementation. Of note, ApoB is significantly increased in patients with type 2 diabetes. However, increases in ApoA-I are more important compared to decrease in ApoB regarding the risks for coronary heart disease in diabetic patients. Moreover, the ApoB/ApoA-I ratio is a better predictor of cardiovascular disease compared to low- and high-density lipoprotein cholesterol (LDL-C and HDL-C, respectively) [172]. Hence, it is likely that RJ contains biologically active substances with insulin-like activity. Similarly, Pourmoradian et al. [173] demonstrated that supplementation with RJ (1000 mg once a day during 8 weeks) decreases FBG and serum glycosylated hemoglobin (HbA1c) levels and increases the insulin concentration in patients with type 2 diabetes. They also observed the antioxidant effect of RJ evidenced through the significant increase in SOD and GPx activities in erythrocytes and a decrease in the MDA concentration. An increase in the total antioxidant capacity and a decrease in insulin resistance after RJ supplementation in patients with type 2 diabetes was also shown by Shidfar et al. [174]. They suggested that RJ can ameliorate insulin resistance via an antioxidant effect and may be beneficial for diabetic patients. Diabetes, as well as other pathophysiological conditions such as hypertension and atherosclerosis, is accompanied with the impairment of peripheral circulation [168,175] and organ injury (e.g., heart, kidney, and brain) due to decreased blood flow. On the other hand, it has been shown that 10H2DA from RJ exerts vasodilatory activity, and consequently, antihypertensive effects in an in vitro assay. Moreover, RJ exerted transient vasodilating effects on canine femoral arteries, which may be relevant for the treatment of diabetic foot ulcers. However, clinical trials did not show thensuperiority of 5% topical RJ compared to the placebo for the treatment of diabetic foot ulcers [175]. Contrary to Siavash et al. [175], Yakoot et al. [176] showed that the new patented topical ointment PEDYPHAR^®^, composed of natural RJ and panthenol, in an innovative, enriched alkaline base, is an effective and safe cream for the treatment of diabetic foot infections. Some of the possible mechanisms of positive actions of RJ and its components are: (i) the powerful antibacterial activity of royalisin against Gram + bacteria including methicillin-resistant *Staphylococcus aureus* at very low concentrations; (ii) the strong antimicrobial, neurogenic, and angiogenic activities of 10-HDA; (iii) the effect of RJ on the differentiation of all types of nerve cells (neurons, astrocytes, and dendrocytes) and an increase in mRNA expression of the potent glial cell line-derived neurotrophic factor; (iv) the stimulation of neurogenesis with the active compound of RJ, AMP N1-oxide, by stem cells through the activation of the signal transducer and activator of transcription 3; (v) the production of type I collagen and bone formation through action on osteoblasts; and (vi) the expression of the vascular endothelial growth factor gene. Panthenol, on other hand, enhanced the oxidative phosphorylation of pyruvate and fatty acid carnitine esters and increased the activity of carnitine palmitoyltransferase. Nevertheless, RJ promoted wound healing in an animal model of diabetes. In addition, it has been shown that RJ induces vasorelaxation by increasing NO production [177]. In particular, it was found that some RJ components act as muscarinic receptor agonist(s), most likely ACh, and induce vasorelaxation by releasing NO from the vascular endothelium of healthy rats. The authors concluded that the vasodilatory ability of RJ may increase the blood mass and blood flow, without affecting systemic circulation. This further implies that RJ may be used to improve the peripheral circulation in healthy individuals, but further studies are needed to clarify the effects (if any) of RJ on the peripheral circulation in patients with severe endothelial dysfunction, such as hypertension and atherosclerosis.

### 5.5. Positive Effect of RJ on Overweight and Obesity

Obesity is one of the major health problem worldwide. Moreover, it is associated with increased cardiovascular risk factors such as dyslipidemia, glucose intolerance, and hypertension [178]. A social context that encourages a sedentary lifestyle, easy, 24 h availability to high-energy food, and large portion sizes, are all components of the obesogenic environment, which is a contributing factor to obesity [179].

According to Vajdi et al. [180] supplementation with RJ reduces body weight (BW) and the body mass index (BMI) at the dosages <3000 mg/day. On the other hand, as mentioned previously, numerous data show that dietary supplementation with RJ improves oxidative stress, inflammation, lipid metabolism, and insulin sensitivity [167,168,169], suggesting its potential use in the prevention of obesity-related metabolic disorders.

In the study of Yoneshiro et al. [181], C57BL/6J mice were fed with four different regimes for 17 weeks: a normal diet (ND), high-fat diet (HFD), HFD with 5% RJ, and HFD with 5% honey bee larva powder (BL). In contrast to BL, RJ ameliorated HFD-induced obesity, hyperglycemia, and hyperinsulinemia, as well as hepatic steatosis by stimulating thermogenesis in brown adipose tissue (BAT). In particular, RJ reduced fat accumulation in white adipose tissue (WAT), increased the thermogenic capacity of BAT, and normalized glucose levels regardless of energy intake and physical activity. The increase in the BAT thermogenic capacity was accompanied with the increased gene and protein expression of mitochondrial uncoupling protein 1 (UCP1) and COX-IV, and increased mitochondrial biogenesis. Hence, RJ may be a novel promising food ingredient to combat obesity and metabolic disorders because it exerts its effects on fat accumulation and hepatic triglycerides without modifying food intake. The authors hypothesized that the activation of the transient receptor potential (TRP) channels’ sympathetic nervous system (SNS)-BAT axis by hydroxydecenoic acid (HDEA) and hydroxydecanoic acid (HDAA) could be the mechanism by which RJ reduces the accumulation of body fat and restores glucose homeostasis. In vitro, HDEA and HDAA stimulate cold-sensitive TRP channels [182]. Based on the mechanism of action of capsinoids that activate TRP channels on sensory neurons in the gastrointestinal tract, enhancing the firing of sympathetic nerves connecting to BAT and thereby increasing thermogenesis [182], the authors speculated that the effects of 10-hydroxy-Trans- 2-decenoic acid (10-HDAE) and 10-HDAA are mediated via TRP channels. BL, which does not contain HDEA or HDAA, had no apparent effect on HFD-induced changes in body weight, blood glucose, and insulin, suggesting that the effects of RJ on BAT thermogenesis were indeed mediated by 10-HDAE and 10-HDAA. Similar results were obtained in obese rats. RJ reduced adiposity, promoted the browning of WAT, and activated BAT thermogenesis by upregulating UCP-1. The expression of the PR domain containing 16 (PRDM16), a modulator of BAT development, was also increased, as well as the expression of p38, bone morphogenetic protein 8B (BMP8B), and CREB1, which are also important for WAT remodeling and BAT activation. The benefits of RJ might be attributed to an increased oxygen metabolism, cellular respiration, and oxidative phosphorylation. In addition, 10-HDA from RJ stimulated thermogenesis and energy expenditure by activating TRPA1 (transient receptor potential Ankyrin 1) and TRPV1 (vanilloid1) channels [180].

In obese/diabetic KK-Ay mice, the effect of RJ supplementation on the enhanced lipolysis and reduced body weight was associated with the increased expression of peroxisome proliferator-activated-alpha (PPAR-α). RJ supplementation in mice on a high-fat diet also reduced inflammation, increased levels of irisin, a myokine whose levels are increased in obese individuals, and promoted the metabolic thermogenesis of BAT [180,181,183,184,185,186].

The hypocholesterolemic efficacy of RJ is yet another important factor when considering its potential use for overweight and obesity. The cholesterol-lowering effects of RJ are mainly attributed to its proteins, particularly MRJP1 and MRJP2. MRJP1 binds bile acids and increases cholesterol excretion by lowering the solubility of cholesterol in the jejunum [187]. Furthermore, MRJP may interfere with the reabsorption of bile acid in the ileum, which could ultimately increase the excretion of fecal steroids. Moreover, RJ increased leptin levels in obese adults. Leptin is a hormone that inhibits hunger and regulates energy balance.

Petelin and co-authors [188] have summarized the most significant positive effects of RJ in overweight adults: RJ decreases the levels of cholesterol and C-reactive protein, the inflammatory marker, and increases the levels of adiponectin, an anti-inflammatory marker. Furthermore, RH strengthens the total antioxidant capacity of the serum and increases the levels of bilirubin, an endogenous antioxidant bilirubin, and the levels of leptin, which acts as a satiety signal [188]. In addition, 10-HDA inhibits adipogenesis through the downregulation of key adipogenic transcription factors such as PPARγ, FABP4, CEBPα, SREBP-1c, and Leptin. According to Pandeya and co-authors [189], RJ may be a potential drug in the treatment of obesity, given that the anti-adipogenic effect of 10-HDA on 3 T3-L1 adipocytes occurs via two mechanisms: the inhibition of the cAMP/PKA pathway and the inhibition of p-Akt- and MAPK-dependent insulin signaling pathways.

### 5.6. Effectiveness of RJ in Reducing Blood Pressure and Protection of Vascular System and Heart

Hypertension is one of the strongest risk factors for cardiovascular diseases in the elderly population and a major risk factor for global disease burden. It may cause the infarction of the myocardium, heart failure, and stroke. There is also a link between hypertension and obesity, dyslipidemia, and insulin resistance, i.e., the metabolic syndrome.

The antihypertensive properties of RJ have been investigated by Tahir et al. [190]. They reported the effects of *trans*-2-octenoic acid and 10-HDA in the control of blood pressure. However, the hypotensive effect of unsaturated fatty acids could be questionable in vivo due to the instability of these acids in the digestive system. Matsui et al. [191] have studied the physiological functions of RJ in spontaneously hypertensive rats. Some peptides from RJ, obtained by enzymatic hydrolysis in the digestive system, act as angiotensin I-converting enzyme (ACE) inhibitors. In general, blood pressure is regulated by the renin-angiotensin system which has an important physiological role in the circulatory system and/or local organs. ACE inhibitor peptides, e.g., IY, VY, and IVY, obtained from protease-treated RJ, are resistant to stomach and bowel proteases. These peptides can control the excretion of other active compounds regulating blood pressure, such as NO, endothelin, or prostaglandins. The antihypertensive effect of RJ peptides has been demonstrated by Tokunaga et al. [192] in spontaneously hypertensive rats, indicating that RJ can be appreciated as a functional food for regulating blood pressure in people with hypertension. Moreover, the intake of RJ hydrolysates reduces a high cholesterol level and increases hemoglobin values in humans, thus having a positive effect on organism homeostasis [187,193,194,195,196,197]. Kashima et al. have identified MRJP1, MRJP2, and MRJP3 as bile acid-binding proteins from RJ [187]. Moreover, the positive effect of MRP1 on hypercholesterolemia was accompanied with the inhibition of corticosterone synthesis. The cholesterol-lowering effect of MRJP1 was due to its ability to interact with bile acids and increase their excretion. MRJP1 also showed a tendency of increasing fecal cholesterol excretion. In addition, it stimulated the hepatic cholesterol catabolism. Interestingly, MRJP1 exhibited better hypocholesterolemic activity than β-sitosterol in rats. Moreover, MRP1 reduced the absorption of cholesterol in Caco-2 cells which are used as a model of intestinal absorption in humans. Hence, it has been suggested that MRJP1 decreases the cholesterol micellar solubility. By suppressing the cholesterol micellar solubility, and due its high bile acid-binding capacity, it is likely that MRJP1 inhibits cholesterol absorption in the jejunum and the reabsorption of bile acids in the ileum, altogether lowering the serum cholesterol level. Pan et al. [197] have shown that RJ may cause hypotension and vasodilation by increasing NO production. They investigated the mechanisms underlying the hypotension and vasorelaxation effects of RJ in spontaneously hypertensive rats (SHR) and the isolated rabbit thoracic aorta rings model. In their study, RJ reduced systolic and diastolic blood pressure with little effect on the heart rate, and increased NO levels. Furthermore, RJ contains muscarinic receptor agonists, likely Ach or Ach-like substances, and induces vasorelaxation by activating the NO/cGMP pathway and calcium channels. Hence, the authors concluded that the oral administration of RJ reduces blood pressure due to the RJ-induced effect of NO on arterial vasodilation. Accordingly, L-NAME (nonselective eNOS inhibitor) and indomethacin (nonspecific COX inhibitor) attenuated this vasodilatory effect, further indicating that some components of RJ act on the muscarinic receptor of vascular endothelial cells and stimulate the release of NO and prostacyclin (PGI2) which act as vasodilators by increasing the cGMP levels. Of note, it has been estimated that RJ contains around 1000 μg/g of ACh [177]. The lack of effect of RJ on the heart rate was explained by two opposite mechanisms that act. While ACh slows down the heart rate, acting on the cardiac muscarinic receptor too, adrenaline speeds up the heart rate via the cardiac beta1 receptor. In addition to ACh-like components, other components from RJ may have effects on blood pressure, including 10-HDA, 10-HDAA, sebacic acid, MRPJPs such as MJP1, and ACE-inhibiting peptides [197].

The cholesterol-lowering effect of RJ has already been mentioned. High blood cholesterol is a risk factor for heart and vascular diseases. These include atherosclerosis, high blood pressure, and other vascular diseases, as well as heart attacks, angina pectoris, and other heart diseases. It also increases the risk of stroke and diabetes. Therefore, it is of great importance that RJ reduces the cholesterol levels in animals and atherosclerosis in humans [193,194,195]. Furthermore, it regulated the lipid metabolism in rats and prevented atherosclerosis in rabbits fed with cholesterol-rich food [197]. More importantly, the oral use or injection of RJ reduced serum lipids and cholesterol in arteriosclerotic patients with moderately high cholesterol levels. Vittek [195] reported the effect of RJ on the reduction of total lipids (around 10%) in serum and the liver, but also on the lowering of the cholesterol level (for 14%). RJ was used at a dose of 5–100 mg daily during at least 2 to 3 months.

The main lipids in plasma are cholesterol, triglycerides, and phospholipids. Lipoproteins, the macromolecular particles made of lipids and proteins, transport cholesterol and tryglicerides through the body. Low-Density Lipoproteins (LDLs) are the main carriers of cholesterol. Therefore, a high level of cholesterol in serum is usually related to abnormally high levels of LDL cholesterol. High LDLs can result from their overproduction or due to the inadequate removal of Very-Low-Density Lipoproteins (VLDLs), an LDL precursor in the hepatic route. Whereas LDLs contain more cholesterol, VLDLs have a higher percentage of triglycerides. There are several categories of VLDLs depending on their size [198]. In plasma, large VLDL particles are eliminated or catabolized to small VLDLs through the lipolysis of triglycerides by lipoprotein lipases attached to the endothelia. Therefore, the VLDL metabolism is one of the regulatory factors of the LDL cholesterol concentration in the blood. Furthermore, VLDLs increase LDLs which have atherogenic properties. Guo et al. [194] have shown that the intake of RJ decreases the total levels of cholesterol and LDLs by decreasing the levels of small VLDLs. Recently, it was suggested that small VLDLs are a powerful independent predictor of atherosclerosis progression and more directly involved in progression than LDLs, which are usually considered the main atherogenic lipoproteins. In addition to the previously explained mechanism of the hypocholesterolemic effect of MRJP1 through the interactions with bile acids and increased excretion of fecal bile acids and fecal cholesterol [197], RJ and MRJPs exert a cholesterol-lowering effect by affecting the cholesterol catabolism in the liver. Moreover, MRJPs may induce the gene expression of the low-density lipoprotein receptor (LDLR) and decrease the expression of the genes involved in cholesterol biosynthesis, such as squalene epoxidase and the sterol regulatory element-binding protein [199,200,201]. RJ proteins including royalisin and degradation products of MRJP1/MRJP3 may have therapeutic beneficial effects on atherosclerosis owing to the reduction of plaque inflammation. These proteins can bind to LDLs and Ox-LDLs and inhibit macrophage proliferation regardless of the presence or absence of LDLs and Ox-LDLs in vitro. According to Sato et al. [199], royalisin and the degradation products of MRJP1 and MRJP3 were bound with an approximately six-times higher affinity to LDLs than to Ox-LDLs, and the authors suggest that further research on RJ proteins may lead to the discovery of new anti-atherosclerotic drugs. It has been shown that RJ can induce the upregulation of cholesterol 7-α-hydroxylase (CYP7A1), a key enzyme for hepatic cholesterol degradation. Therefore, the regulation of CYP7A1 gene expression could be an important strategy against hypercholesterolemia and atherosclerosis.

The daily intake of RJ may help to normalize HDL-C, LDL-C, and total cholesterol (TC). In healthy volunteers, an intake of RJ (6 g/day during 4 weeks) decreased LDL-C and TC without affecting HDL-C and TG levels. In another study, Lambrinoudaki et al. [202] analyzed cardiovascular risk markers in postmenopausal women after three months of RJ administration (150 mg daily). They observed prominent changes in lipid parameters. HDL-C values were increased, whereas levels of LDL-C and total cholesterol (TC) were reduced. According to recently performed meta-analyses related to studies with a long-term follow-up, RJ reduces TC and LDL-C levels, but does not improve TG and LDL-C values. The cholesterol-lowering effect of RJ could be mediated by its estrogenic activity. The estrogenic action of RJ and 10-HDA have been shown in cultured cells and in rats [28,203]. On the other hand, it is well known that estrogen and estrogen modulators may reduce serum cholesterol levels in rats [28,127,203,204] However, purified MRJP1 also showed a hypocholerostemic effect which was, therefore, independent of the estrogenic effect of 10-HDA [187]. Altogether, these findings indicate the possibility of using RJ as an anti-atherogenic functional food.

### 5.7. Estrogen Effect of Royal Jelly

Mishima et al. [28] have shown the weak estrogenic effect of RJ. In MCF-7 breast cancer cells with estrogen receptors, RJ increased the proliferation rate. The effect was counteracted with tamoxifen, a chemotherapy drug that acts as an antagonist of the estrogen receptor. Furthermore, the subcutaneous injection of RJ restored the expression of the gene for the vascular endothelial growth factor (VEGF) in ovariectomized mice. Estrogen has an essential role in the bone metabolism, particularly in women. Accordingly, a lack of estrogen is the main factor of bone loss and the development of osteoporosis in postmenopausal women, whereas the dietary intake of phytoestrogens has been suggested as an option in the prevention of osteoporosis [127,204,205]. For example, in osteoblastic MC3T3-E1 cells, the soy isoflavones, genistein and daidzein, increased mineralization, likely through their estrogenic properties [205]. RJ stimulated the proliferation of the same cells, as well as the production of type I collagen. The long-term oral intake of RJ also increased the ash content of the tibiae in female mice, indicating that it may stimulate bone formation by acting on osteoblasts. Besides increasing the tibial weight, RJ also induced the expression of genes participating in the formation of the extracellular matrix. The same effects have been observed in a rat model, suggesting that RJ may be used as a dietary supplement in osteoporosis prevention [87,206].

The daily use of RJ improved ovarian hormone secretion, follicle development, and oocyte maturation, ameliorated the redox status in the oocytes, and activated the glucose pathway in cumulus cells, leading to the improvement of the fertility parameters, at least in rats. In ovariectomized rats, it has been shown that RJ competes with E2 for binding to ERα and ERβ, but with a weaker affinity compared to phytoestrogens. However, despite their weak affinity, RJ may restore VEGF expression.

According to Suzuki et al. [207], some lipid components from RJ (10-HDA, 10-HDAA, 10-HDAE, and 24-methylenecholesterol) inhibited the binding of 17β estradiol to ERβ, but were without the effect of binding to ERα. Husein and Kridli [208] as well as Huseini and Haddad [209] have confirmed the effect of RJ on the reproductive potential in sheep. RJ combined with progesterone can increase the reproductive potential in sheep. Ovulation was advanced, and follicular growth and estradiol secretion were increased. Similar data were obtained in laying hens where RJ at doses of 100 or 200 mg/kg body weight attenuated the negative effects of senescence, improved the morphology of the reproductive tract, stimulated follicular growth and the rate of egg production, and improved the internal egg quality parameters in elderly laying hens [210].

In addition, RJ has an important role in the regulation of the hormonal balance. It increases the production of testosterone and estrogen. 10-HDA in particular may improve the hormonal balance by enhancing the production of the ovulation hormones and by preserving the follicular pool [211].

Luteinizing hormone (LH) and follicle-stimulating hormone (FSH) are gonadal hormones important for reproduction and aging. They are regulated by estrogen and inhibin. In young women, high estrogen levels maintain an increased level of FSH and a decreased level of LH [121,211]. In menopause, the reduction in the follicle size results in abnormal FSH secretion. RJ in the diet may improve these changes as the presence of 10-HDA increases estrogen production and maintains low levels of FSH and LH in the serum. As mentioned previously, 10-HDA also prevents follicular depletion and improves hormonal regulation [211]. RJ (1 g/kg) can reduce premenstrual symptoms and improve quality of life in the menopause and postmenopause phase by relieving genito-urinary syndromes. Furthermore, RJ can alleviate postmenopausal symptoms such as hot flushes, night sweats, and other symptoms. In addition, menopause is characterized by estrogen depletion which affects the autonomic nervous system, leading to the development of neurodegenerative diseases like Alzheimer’s disease, mood disorders (e.g., anxiety, depression), headaches, and low-back pain. Through its estrogenic activity, RJ significantly reduces these symptoms without side effects.

It should be emphasized that the content of antioxidants in RJ may overcome free-radicals-induced oxidative stress in women before getting pregnant. Good nutrition before conception can reduce the risk of complications at the time of conception that could threaten the health of the offspring. Stress that occurs in women during preconception may greatly affect the health of the mother and cause menstrual disorders, polycystic ovary syndrome (PCOS), and infertility. Oxidative stress in preconceived women can be treated with non-pharmacological therapy, one of which is RJ. Up to 20% of women of reproductive age are diagnosed with PCOS. PCOS is an endocrine disorder characterized by high androgens and ovulatory dysfunction, which consequently leads to infertility and mental illness such as anxiety and depression. Hamid et al. [212] investigated the effect of RJ, applied at doses of 100 mg/kg, 200 mg/kg, and 400 mg/kg over a period of 4 weeks, on the hormonal profile of PCOS animal models. The most effective dose of RJ was 200 mg/kg which improved the regularity of the estrus cycle, histology and ovarian function, the level of reproductive hormones (LH, testosteron, FSH, and estradiol), as well as the antioxidant status of the ovaries (MDA, total antioxidant capacity (TAC), GPx). The authors suggested that royalactin is responsible for the histological changes of the ovaries. Furthermore, data showing the protective effect of RJ on the liver and kidneys through the reduction of lipid peroxidation and increased the GSH content, as well as the presence of 10-HDA which has an anti-inflammatory effect, indicating the potential of RJ in reducing oxidative stress. In addition, the positive effect of RJ could be due to its effect on the accelerated growth and development of follicles that secrete estradiol to stimulate the uterus, increasing the level of LH and triggering ovulation [213]. MRJP1 from RJ, through its hypocholesterolemic activity, may reduce the synthesis of corticosterone, which further may affect the energy regulation, immune reactions, and stress response. Moreover, MRJPs increase the estrogenic activity and antioxidant potential of the reproductive system, upregulate the gene expression of the estrogen receptor beta (ER beta), and stimulate hormone secretion and ovary development in female mice [214].

### 5.8. Effect of Royal Jelly on Spematogenesis

RJ improves fertility in both men and women. In men, it increases the quality of the sperm, whereas in women, it increases the quality of the ovules. Previous studies have shown that RJ increases the levels of male hormones, improves the sperm count, motility and morphology, and reduces the damage of the reproductive tract induced by various toxic stimuli. Shahzad et al. [215] showed that RJ (0.1–0.3%) protects spermatozoa during freezing and increases their mobility. The increased motility of sperm after freezing may be due to the high antioxidant capacity of RJ, as well as the presence of active biological amino acids such as aspartic acid, cysteine, glycine, cysteine, tyrosine, leucine, lysine, isoleucine, and valine. Thus, it is hypothesized that proline may protect cell membranes from stressful conditions, while cysteine, a powerful antioxidant, may neutralize free radicals and participate in glutathione synthesis during the freezing period [215]. RJ showed potent antioxidant activity against Cd-induced oxidative damage in the testicular tissue. Cd-induced testicular dysfunction was accompanied with increased LPO and nitrate/nitrite levels and the reduced activity of cellular antioxidant and detoxifying molecules, including GSH, GPx, glutathione reductase, SOD, CAT, and Nrf2. Moreover, RJ supplementation downregulated the gene expression of the cytochrome P450 4A14 (CYP4A14) which stimulates the production of intracellular free radicals and catalyzes lipid peroxidation. The antioxidative effect of RJ observed in Cd-induced oxidative stress was likely mediated through the upregulation of Nrf2. Nrf2 provides protection against ROS via different mechanisms, including the increase in GSH synthesis, the upregulation of antioxidant and detoxifying enzymes, and the degradation of superoxide and peroxide radicals by GPx and SOD. In another study performed in male rabbits, RJ applied at a dose of 150 mg/kg body weight showed positive effects on libido, glucose, blood testosterone, plasma total proteins, fertility, and sperm production and quality [216]. The protective effect of RJ on hormonal parameters has also been observed in aluminum chloride (AlCl3)-induced toxicity. RJ attenuated the AlCl3-induced changes of gonadotropin levels (FSH, LH) and testosterone, as well as levels of the thyroid-stimulating hormone (TSH), thyroxine (T4), triiodothyronine (T3), and the T3/T4 ratio [217]. AlCl3 also resulted in oligospermia, hypoplasia, constriction of the blood vessels, and exfoliation of tubules. These changes were similarly attenuated by RJ. Finally, RJ was beneficial in adult rats that received hydrogen peroxide (oxidative stress inducer). RJ improved the number of spermatozoa, percentage of live spermatozoa, testosterone level, and levels of glutathione and malondialdehyde [218,219]. RJ treatment at a dose of 100 mg/kg/day for 4 weeks protected the testicular structure from the damaging effect of diabetic oxidative stress through its antioxidant effect, thus preserving male fertility [218].

### 5.9. Royal Jelly and Osteoporosis

**Figure 4:**
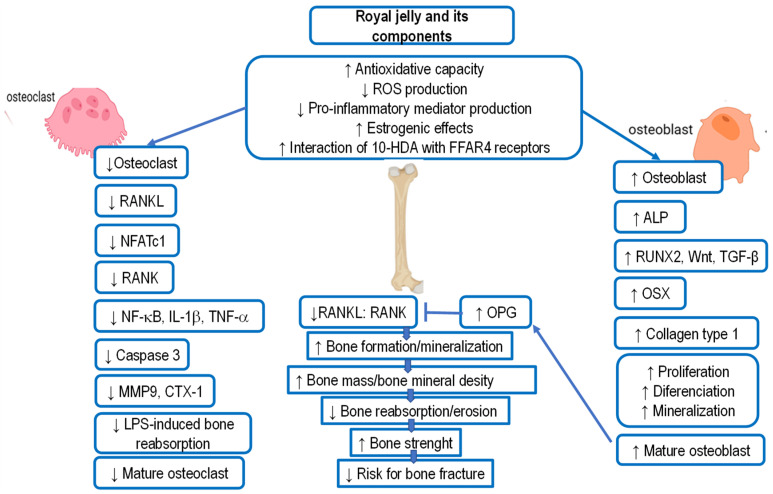
The effects of royal jelly (RJ) and its components on bone mineral density and strength. The key mechanisms of RJ action are based on increasing the antioxidant capacity, reducing oxidative stress, and regulating the production of inflammatory mediators, estrogenic activity, and interaction of 10-HDA with FFAR4 receptor. RJ and its components, such as 10-HDA, have an inhibitory effect on osteoclast differentiation and function by suppressing the NF-κB signaling pathway and its downstream molecules including NFATc1, CtsK, TRAP, V-ATPase D2, and MMP9, via FFAR4. Moreover, the inhibitory effect of RJ on ROS reduces RANKL, TRAP, NF-B, and caspase-3 activities in osteoclasts. RJ upregulates Runx2, Wnt, TGF-β, Osterix, Osteocalcin, and ALP by inhibiting accumulation of ROS. This contributes to the increased bone formation/mineralization and decreased bone resorption, resulting in increased bone mass, bone mineral density, and bone strength, and decreased risk of bone fractures. Note: 10-HDA, 10-hydroxy-2-decenoic acid; BMD, bone mineral density; CTX, C-terminal telopeptide; FFAR4, free fatty acid receptor 4; IL-1β, interleukin 1 beta; LPS, lpopolysaccharide; RANK, receptor activator of nuclear factor-κB; RANKL, receptor activator of NF-kB ligand; RUNX2, runt-related transcription factor 2; NFAT c1, nuclear factor of activated T cell; MMP-9, matrix metalloproteinase 9; NF-κB, nuclear factor-kappa B; OPG, osteoprotegerin; Osx, Osterix, transcription factor; TGF-β, transforming growth factor;TNF-α, tumor necrosis factor alpha. ↑ upregulated; ↓ downregulated.

A few studies have investigated the effects of RJ on the bone metabolism and related cellular functions. Bone loss has become a major problem in postmenopausal women with significant morbidity and mortality. In both postmenopausal women and ovariectomized rats, bone loss is a result of an ovarian hormone deficiency. The study of Kafadar et al. [220] has shown that RJ and bee pollen reduce osteoporotic bone loss in an oophorectomized rat model. The total body bone mineral density (BMD) was similar in all groups, whereas BMD of the lumbar spine and proximal femur were higher in animals that received RJ or bee pollen. The calcium and phosphate levels in bone tissue were also higher in RJ and bee pollen groups, suggesting that both RJ and bee pollen could be beneficial in clinical practice. Hatori et al. obtained similar results [221]. They studied the effects of royal jelly protein (RJP) on the bone mineral density and strength in ovariectomized Sprague–Dawley rats that received RJ for 8 weeks. RJP reduced the BMD decrease in the lumber spine and proximal tibia and improved bone strength. These findings indicate that RJP is an option for suppressing trabecular bone loss in ovariectomized rats and that RJP may prevent bone abnormalities induced by a sex hormone deficiency. Tsuchiya et al. [206] demonstrated that 10-HDA, a key RJ constituent, protects against bone loss by suppressing osteoclastogenesis. In particular, the binding of 10-HDA to free fatty acid receptor 4 (FFAR4) inhibited the activation of the NF-κB pathway in osteoclasts (see Figure 4). This further inhibited the induction of the nuclear factor of activated T cells c1 (NFATc1), a key transcription factor for osteoclast differentiation [206]. The authors suggested that 10-HDA inhibits osteoclast differentiation and function by suppressing the NF-κB signaling pathway and its downstream molecules including NFATc1, CtsK, TRAP, V-ATPase D2, and MMP-9 through the FFAR4. Considering that FFAR4 is implicated in various pathological and physiological processes, including inflammation, the secretion of glucagon-like peptide-1, adipocyte differentiation, insulin sensitization, the regulation of appetite, and tumor progression, FFAR4 represents a promising target not only for osteoporosis, but also for the treatment of obesity-related metabolic disorders, such as cancer. It was also shown that RJ reduces ovariectomy-induced bone loss by suppressing osteoclastogenesis through the inhibition of RANKL (receptor activator of nuclear factor-kB (NF-κB) ligand)-induced osteoclastogenesis [206]. On the contrary, Shimizu, et al. [222] suggested that the main effect of RJ is not the prevention of bone loss, but the improvement of bone strength.

Moreover, the weak estrogen-like activity of RJ has been shown in cultured osteoblasts [38,86]. As post-menopausal osteoporosis is mainly caused by the reduced production of estrogen, it is likely that the estrogen-like activity of RJ may help in alleviating post-menopausal osteoporosis [121,223,224,225]. Since RJ also contains the testosterone, it may also be effective in men’s osteoporosis induced by a lack of androgen. Besides estrogen-like activity, RJ stimulates the migration of dermal fibroblasts, increases the production of collagen [29,66], and improves muscle mass, strength, and function. The underlying mechanisms behind the effects on skeletal muscles likely include anti-inflammatory and anti-oxidative properties, improved metabolic regulation, the activation of muscle stem cells, increased blood supply, the suppressed expression of catabolic genes, and the stimulation of peripheral neuronal regeneration [52,58,103,108,147]. Therefore, natural products that do not show significant side effects, such as RJ, can be used as an alternative or adjuvant therapy for the treatment of osteoporosis. Due to its estrogen-like effect, RJ may also decrease the risk of osteoporotic fractures.

### 5.10. Anticancer Effectiveness of Royal Jelly and Hematopoiesis Stimulation

The anticancer effect of RJ has been investigated in various models, such as 6C_3_HED lymphosarcoma, TA_3_ mammary carcinoma, Ehrlich ascites carcinoma, leukemia in AKR mice, sarcoma 180, Solid Ehrlich carcinoma, lymphatic L1210, and P388 leukemia [226,227,228,229]. The effect of RJ was observed in slow-growing tumors but not in fast-growing ones. The anticancer activity was attributed to 10-HDA and saturated dicarboxylic acids (succinic, glutaric, adipic, pimelic, suberic, azelaic, and sebacic acid). Oršolić et al. [228,229] have shown that RJ reduces the number of tumor nodules only if administered together with tumor cells. Furthermore, they showed that RJ has significant immunostimulatory properties [230]. Similarly, Kimura [231] demonstrated the inhibitory effect of RJ (at doses of 300 or 600 mg/kg) on tumor growth and/or metastasis in the liver or lungs. Although the antitumor activity of RJ has been confirmed in many studies, its antimetastatic effect may depend on the method of administration, and consequently, on the close contact of RJ, especially 10-HDA, with tumor cells. Thus, in contrast to intravenous administration, the intraperitoneal or subcutaneous application of RJ had no effect on the formation of lung metastases [228]. In particular, RJ inhibited tumor-induced angiogenesis and/or stimulated the immune response by enhancing the production of T lymphocytes involved in the elimination of viruses and tumor cells [232]. In B16F1 melanoma cells, the antiproliferative effect of RJ was achieved through the inhibition of microphthalmia-associated transcription factor and tyrosinase-related protein 1 (TRP-1) and TRP-2, which ultimately suppresses melanin production [233]. Accordingly, RJ reduced skin pigmentation in mice which further indicates its potential for the production of skin care products [233]. In another study, a derivative of 10-HDA, 4-hydroperoxy-2-decenoic acid ethyl ester (HPO-DAEE), induced the apoptosis of A549 human lung cancer cells by activating the ROS-ERK-p38 and C/EBP homologous protein (CHOP) pathways [234,235]. Hence, the pro-apoptotic effect of HPO-DAEE was, at least partially, dependent on signaling pathways related to endoplasmic reticulum stress [235]. Moreover, 10-HDA has been recognized as an inhibitor of MMPs. Under inflammatory conditions, these endopeptidases are activated by proteolytic cleavage. They degrade matrix and non-matrix proteins and promote angiogenesis, the infiltration of cancer cells, and metastasis. The anticancer effects of RJ have also been demonstrated on HeLa cells (cervical cancer in women), particularly for the protein fraction RJP30 that induced 50% cytotoxicity [112], as well as in Lewis lung carcinoma and colorectal adenocarcinoma cells [231,236] where the effect was attributed to 10-HDA. The efficacy of 10-HDA was also reported in transplantable leukemia in AKR mice and various ascitic tumor cell lines in mice. As mentioned in previous sections, 10-HDA acts as a potent HDAC inhibitor and suppresses proliferation by inhibiting PI3K/Akt signaling [47,88]. The lipophilic extract of RJ demonstrated the antiproliferative effects in human neuroblastoma cells (SH-SY5Y) and human glioblastoma (U373) [237,238]. Treatment with a high dose of RJ significantly increased the apoptotic cell population of both SH-SY5Y-human neuroblastoma and U373-human glioblastoma and arrested the cell cycle at the G0–G1 phase in SH-SY5Y cells and at G2–M in U373 cells. Furthermore, 10-HDA and 4HPO-DAEE inhibited the proliferation of leukemia cells THP-1 by inhibiting HDAC activity. In general, HDAC inhibitors are strong anti-inflammatory drugs [68,239]. Accordingly, in human colon cancer cells (WiDr cells), the production of pro-inflammatory cytokines IL-8, IL-1β, and TNF-α was modulated by 10-HDA [68]. Levels of IL-8 were largely attenuated following treatment with 3 mM 10-HDA, whereas levels of IL-1β and TNF-α were only slightly, although significantly, decreased. On the other hand, RJ increased the amount of IL-1ra. Hence, it seems that RJ may be effective in controlling chronic inflammation and carcinogenesis due to its ability to inhibit NF-κB and the production of TNF-α, IL-1β, and IL-8. In addition, in J5 cells (human hepatocellular carcinoma), RJ interfered with the activation of arylamine carcinogens. RJ inhibited the N-acetylation of 2-aminofluorene (2-AF), i.e., it inhibited the activity of N-acetyltransferases and the generation of 2-AF metabolites [240]. Of note, it has been shown that N-acetyltransferases are involved in chemical carcinogenesis as higher N-acetyltransferase activity increases sensitivity to the mutagenic effects of arylamines. Taken together, the pharmacological effects of 10-HDA are mainly mediated through the anti-tumor activity, inhibition of angiogenesis, and immunomodulatory properties.

A study by Abu-Serie and Habashy [241] revealed the potent anticancer effect of MRJP2 and its isoform X1. These proteins induced caspase-dependent apoptosis, the reduced expression of Ki-67, a marker for proliferation, and the regulated expression of Bcl-2 and p53 in HepG2 cells. In fact, MRJP2 increased the number of early and late apoptotic cells more than doxorubicin. Therefore, the authors suggested that MRJP2 and X1 can be a promising strategy against hepatic cancer [241].

The effects of RJ have also been studied in Ehrlich solid and ascites tumors (EST and EAT) [242,243]. Albalawi et al. showed that the treatment of EST-suffering mice with RJ at the doses of 200 and 400 mg/kg causes a significant reduction in the tumor volume and inhibition rate, body weight, tumor markers such as the serum level of alpha-fetoprotein (AFP), and carcinoembryonic antigen tumors (CAE). In addition to tumor markers (AFP and CAE), a decrease in the serum level of the liver and kidneys, LPO and NO, TNF-α level, as well as the expression level of Bcl-2 was observed in the RJ-treated group compared to the control EST group. The level of antioxidant enzymes of GPx, CAT, and SOD and the expression level of caspase-3 and Bax genes was significantly increased in RJ-treated groups.

Cells of EAT grow fast in most mice strains and disturb various hematopoietic and immune parameters, causing the host’s death even in the presence of a small number of cancer cells. EAT reduces the number of granulocyte–macrophage colonies in the spleen and the induction of natural suppressor cells. Phagocytes, especially macrophages and neutrophils, play an important role in host defense against tumor growth. One of the main characteristics of these cells is their ability to migrate inside the inflamed area. Animal model studies have shown that tumor cells can produce factors that impair the inflammatory response, promoting tumor growth. More precisely, tumor cells can stimulate the suppressor activity of the host macrophages. Macrophages have a dual role in cancer, which is a result of their plasticity in response to environmental conditions. Macrophages can kill tumor cells, mediate cellular cytotoxicity and antibody-dependent phagocytosis, induce vascular damage and tumor necrosis, and activate innate or adaptive tumor resistance mechanisms mediated by lymphoid cells. In contrast, tumor cells can evade macrophage cytotoxicity and redirect macrophage activities to contribute to cancer progression and metastasis. Besides the inappropriate accumulation of phagocytes in the inflamed areas, other cellular functions are also disturbed in tumor carriers, including the reaction of granulocyte–macrophage colonies on the colony activation factor. In that regard, treatment with RJ (at doses 0.5, 1, and 1.5 g/kg) did not increase the number of bone marrow cells and granulocyte–macrophage colonies in the spleen of the healthy mice, whereas in EAT-bearing mice, the number of bone marrow cells and number of cells in peripheral circulation were increased. The increased number of long-living stem cells, as well as the number of granulocyte–macrophage colonies, have been observed in cell cultures treated with RJ. Thus, RJ may enhance the host resistance against tumors through stimulating macrophage function, antibody production, and immunocompetent cell proliferation. RJ pre-treatment to EAT-bearing mice also increased the phagocytic function of macrophages, the activity of T cells, and the activity of B cells. Furthermore, RJ pretreatment effectively decreased the proliferation of EAT cells in the peritoneal fluid and decreased the viability of EAT cells in vitro. The size of the solid Ehrlich tumor in the thigh muscle of the mice was also significantly reduced [226].

It has been shown that RJ promotes macrophage recovery, their number, and activity. Accordingly, the improved recovery of hematological parameters in EAT-bearing mice was reflected in prolonged survival. In general, tumor associated macrophages (TAM) stimulate tumor growth and inhibit the activity of NK cells and T lymphocytes [36,37,243]. This indicates that certain RJ components prevent the tumor-induced suppression of macrophages and stimulate their immunomodulatory activity, consequently increasing the control of tumor growth and spread. RJ has the ability to protect against macrophages immunosuppression caused by tumor secreting substances. RJ inhibited the increase in prostaglandin (PGE_2_) by 30%, which inhibited the tumoricidal activity of macrophages. PGE_2_ alters the immunological response including the mitogenesis of T lymphocytes, cytokine production, and tumor cytotoxicity mediated by macrophages and NK cells. Furthermore, RJ can inhibit the production of anti-inflammatory cytokines, such as TNF-α, IL-6, and IL-1, without reducing the cytotoxic effect of macrophages stimulated with LPS and/or LPS + IFN-γ [36]. Bincoletto et al. [243] argued that the RJ-induced reduction of PGE_2_ in EAT-bearing mice is the main reason for their survival. RJ is capable of modifying the biological response, reducing myelosuppression, and increasing the antitumor effect. The results of our studies on rats confirm those claims [227,228,229,230]. In LPS + IFN-γ-stimulated macrophages, RJ (2.5 mg/mL) reduced PGE_2_ synthesis by 63% in comparison to control macrophages [36]. These results also indicate the possibility of the effective use of RJ as a food supplement for increasing quality of life in patients with autoimmune diseases such as rheumatoid arthritis and inflammatory bowel diseases. The inhibition of PGE2 is important as PGE2 is involved in the process of carcinogenesis. PGE2 promotes the proliferation and invasion of tumor cells and inhibits the apoptotic process in various types of cancers [244,245]. In addition, it regulates the proliferation of lymphocytes and suppresses the tumoricidal activity of normal macrophages. On the contrary, RJ reverses myelosuppression in mice with EAT, promotes the hematopoietic function of the spleen, reduces PGE2 production, and prolongs the survival of mice. The inhibition of PGE2 synthesis reactivates the immune system response, stimulates the mitosis of T lymphocytes and lymphokine production, and increases the tumor-killing activity of macrophages and natural killer cells [229,243]. The stimulation of monocyte–macrophage cells was confirmed in the research of Wang et al. [246] who used RJ to stimulate human monocytic cells (MNC). After MNC stimulation, their filtered conditioning medium (MNC-CM) was used to treat U937 cells. Four conditioned media inhibited U937 cell growth by 25.9–50.6%. CD11b and CD14 expressions on the treated U937 cells were increased by 38.1–49.8% and 43.4–52.0%, respectively. In this way, Wang et al. [246] confirmed that RJ stimulates human mononuclear cells to secrete cytokines (IL-1β, TNF-α, and IFN-γ) and NO in MNC-CM, which inhibit the growth of U937 cells and induce their differentiation, which can be a natural and alternative pathway in the treatment of leukemia.

A 350 kDa glycoprotein (apisin) has been identified as an RJ component that stimulates the proliferation of human monocytes. The protective effect of RJ on hematopoiesis has also been observed in mice irradiated with X-rays [247,248,249]; RJ prevented the radiation-induced damage of stem cells. The immunostimulatory properties of RJ combined with propolis (product called “Apinhalin”) have been observed in granulocytopoiesis and lymphopoiesis following irradiation with the dose of 3 Gy and/or 5 Gy. The regeneration of cells was observed after only 10 days in comparison to the control group [250]. Moreover, RJ stimulated the production of antibodies and the proliferation of immunocompetent cells [230].

RJ also inhibited the bisphenol A-induced proliferation of human breast cancer cells (MCF-7) [34]. Bisphenol A is an environmental estrogen widely used in the production of polycarbonate plastics and polyepoxide. It is an endocrine disruptor that shows a weak binding affinity for estrogen receptors. Consequently, it negatively affects human health, especially in women [251]. In MCF-7 breast cancer cells, RJ prevented the stimulatory effect of bisphenol A on cell proliferation. The authors suggested that RJ inhibited estradiol-induced intracellular signaling events, not the binding of estradiol to the estrogen receptor.

In the renal cell carcinoma (RCC) model, it was shown that 10-HDA suppresses tumor growth, invasion, and metastasis through its strong anti-inflammatory effect. 10-HDA inhibited the secretion of TNF-α which promotes the proliferation of cancer cells and their malignant transformation. More importantly, the reduced concentration of TNF-α and TGF-β following RJ intake reduced the paraneoplastic syndrome in RCC patients [252]. Thus, the anti-cancer and anti-inflammatory action of 10-HDA was mediated through the reduced production of pro-inflammatory cytokines (TNF-α, IL-1β, and IL-8). Another fatty acid from RJ, 3,10-dihydroxydecanoic acid, stimulated the maturation of monocyte-derived dendritic cells and their Th1 polarizing capability, overall increasing the anti-cancer response [253]. Furthermore, 10-HDA may reduce the tumor vascularization. In addition, it has confirmed that RJ nanoparticles can alleviate the experimental model of breast cancer through suppressing regulatory T cells and upregulating Th1 cells [254]. Furthermore, new research from Xu and co-authors confirms that RJA exerts antitumor effects by affecting the glycolytic pathway in Human hepatocellular carcinoma (HCC) through the lactate modification pathway [255].

In a 4T1 breast cancer mice model, RJ also reduced the tumor weight, particularly when applied as a prophylactic treatment. The anticancer effect was accompanied with the increased SOD activity, total antioxidant capacity (T-AOC), and glutathione reductase activity in the liver, kidneys, and serum [256].

### 5.11. RJ as Protective Agent against Pro-Oxidants’ Toxicity and Chemo- and Radiotherapy Side Effects

Cytostatics have multiple effects on tumor cells: they prevent cell division, stop the division of already-dividing cells, induce spontaneous cell death, and reduce the growth of blood vessels that consequently decreases the delivery of food and oxygen into the tumor tissue. As cytostatics are not selective, they also act on healthy cells, causing various unwanted effects. All cytostatics cause side effects, some of which are common to all cytostatics, and others specific to individual cytostatics.

The most important unwanted consequences or side effects of chemotherapy are: bone marrow suppression (myelosuppression or myelotoxicity); weakness, nausea, and vomiting; oral mucositis (inflammation of the mucous membranes in the mouth and throat), stomatitis (inflammation of the membranes and tissues in the oral cavity and the entire mucosa of the digestive tract), and esophagitis (inflammation of the mucous membrane of the esophagus); diarrhea and dehydration; constipation; allergic reactions and anaphylaxis; hair loss; extravasation (exit of cytostatics from the blood vessel into the surrounding tissue); changes in the sense of taste; anemia due to the reduced number of erythrocytes, shortness of breath, and chronic fatigue; bleeding and bruising as a result of thrombocytopenia; phlebitis (inflammation and the formation of clots in superficial veins); increased infection risk (due to the reduced number of leukocytes); febrile neutropenia (a result of a reduced number of granulocytes); cardiac toxicity, nephrotoxicity, and bladder damage; pulmonary toxicity; neurological toxicity; gonadal dysfunction; hepatotoxicity; induction of secondary tumors; and tumor rapid disintegration (lysis) syndrome, among others [257]. The side effects can be as detrimental as the cancer itself and may greatly reduce one’s quality of life. Moreover, the addition of other drugs to ameliorate the side effects of the anticancer therapy may trigger other health hazards. The polypharmacy may result in predictable and unexpected drug–drug interactions, posing the additional risk to the patient’s health [258].

One of the main challenges in cancer therapy is to find effective drugs with minimal adverse effects. In that regard, natural products may minimize the unwanted side effects. They also may act synergistically with standard therapy, improve the efficacy of cytostatics, and improve the quality of life of cancer patients [252,253,257,258,259]. Moreover, natural products, such as RJ, are readily available, economical, and relatively safe.

Many beneficial effects of RJ in chemotherapy-induced toxicity have been reported. RJ protected lymphocytes against doxorubicin-induced genotoxicity. It improved the antioxidative response and increased the Nrf2/Bax and Bcl-2/Bax ratio [97,252,260,261,262]. Moreover, RJ inhibited the expression of caspase-3 and increased the expression of the anti-apoptotic protein Bcl-2 in the liver and kidneys of the cisplatin-treated rats [263,264]. On the contrary, RJ downregulated the expression of Bax in rats treated with cyclophosphamide [265]. It is likely that the anti-apoptotic effect of RJ might be assigned to its antioxidant capacity [260,261,262,263,264,265,266].

Previous studies have indicated that MRJPs contribute to the regulation of immune functions by stimulating macrophages and by attenuating the production of inflammatory mediators, at least in animal models [242]. Wang et al. [76] suggested that MRJPs promote immune functions in cyclophosphamide-induced mice by increasing the number of white blood cells, the production of anti-inflammatory cytokines IL-4 and IL-6, and the proliferation ability of T/B-lymphocytes in the spleen by modulating the composition of the immune-associated intestinal flora. The protective effect of RJ against cyclophosphamide-induced thrombocytopenia, as well as bone marrow, spleen, and testicular damages in rats, was confirmed by Khazaei and coauthors [267]. RJ pretreatment normalized the number of platelets, white and red blood cells, serum levels of platelet factor 4 (PF4), nitric oxide (NO) and ferric-reducing antioxidant power (FRAP), levels of serum biochemical factors, and histological structures of bone marrow, spleen, and testes [267]. Furthermore, RJ and 10-HDA reduced the weight loss in the body, thymus, and spleen of the cyclophosphamide-induced mice, improved the thymus/spleen indexes, stimulated the pathways involved in DNA/RNA/protein activities, restored the proliferative ability of cells in the thymus and spleen, and promoted the activities of the T and B lymphocytes [268]. It seems that RJ provides protection to many tissues and organs injured by chemotherapy, especially the liver and kidneys, through its antioxidant, anti-inflammatory, and anti-apoptotic activities.

Furthermore, RJ can modulate oxidative stress and apoptosis in the liver and kidneys of rats treated with cisplatin. Thus, prophylaxis with RJ significantly reduced the severity of lipid peroxidation in the liver and kidneys. It is considered that the biologically active, free amino acids such as aspartic acid, L-cysteine, cystine, tyrosine, glycine, lysine, leucine, isoleucine, and valine mediate the antioxidative effects of RJ [269]. In addition, the beneficial effect of RJ on the antioxidative status is visible through the increased GSH content and increased activities of antioxidative enzymes GPx, glutathione–S–transferase and SOD. In addition to the positive effects of RJ on chemotherapy-induced cellular damage, RJ ameliorated the damage of liver cells induced by exposure to tetrachloromethane, cadmium, and paracetamol and reduced the genotoxic and nephrotoxic effects of these chemicals [95,270,271]. ROS produced during the toxic attack of these compounds resulted in lipid peroxidation and DNA and protein oxidation. However, RJ inhibited lipid peroxidation and preserved the structure of the biological membranes, membrane potential, and permeability for various ions.

Different mechanisms of the action of RJ have been elucidated following treatment with compounds such as cyclophosphamide, bleomycin, doxorubicin, 5-fluorouracil (5-FU), thymoquinone, cisplatin, hydroxyurea, temozolomide, interferon alpha, and GE132 plus (a nutraceutical supplement) [250,251,263,265,272]. Thus, RJ reduced genotoxicity and DNA damage when used with cyclophosphamide. Following treatment with 5-FU, RJ reduced the cell viability and IC50 of 5-FU in colorectal cancer cells [273,274]. It is also capable of initiating apoptotic events in breast or liver and kidney cancer cells in animals treated with thymoquinone or cisplatin, respectively [269,275]. When combined with GE132, interferon alpha, or temozolomide, RJ increased the efficacy of these drugs, further exacerbating their anti-proliferative action [276,277]. Numerous studies have demonstrated the synergistic effect of RJ and chemotherapeutic drugs. When applied together, they increase the death of tumor cells, strengthen the protection by immune cells, and protect cells exposed to harmful drugs such as kidney and liver cells.

Radiotherapy is an important therapeutic modality in cancer therapy. The main objective of radiation treatment is to apply an effective dose of ionizing radiation to eliminate cancer cells in a well-defined cancer volume, with minimal side effects to the surrounding healthy tissue. However, the generation of free radical metabolites may lead to the injury of normal tissue, limiting the effectiveness of the therapy. RJ has shown the anti-inflammatory, DNA-protective, and anti-tumor effects in animal models. It may largely reduce the adverse effects of reactive species and limit the cellular damage induced by reactive oxygen and nitrogen species derived through radiotherapy or chemotherapy. In the adult male Sprague–Dawley rats, exposure of the whole body to irradiation induced prominent liver failure, characterized by a marked increase in serum AST, ALT, cholesterol, and triglyceride levels, and with the increased production of MDA and reduced levels of GPx, CAT and SOD in the lung and liver tissue. Pre- and post-treatment with RJ markedly decreased the oxidative stress parameters and ameliorated changes of biochemical parameters, increasing the antioxidant activities [249,278]. The authors suggested that the protective effect of RJ in irradiated animals may be due to the improved activity of endogenous antioxidants, the free radical scavenging activity of RJ, and decreased lipid peroxidation. In addition, the antioxidative, as well as the anti-inflammatory effects of RJ, may be achieved through the increased expression of Nrf2 and decreased levels of NF-κB and pro-inflammatory cytokines. The data of Sarhan et al. [279] have shown that the whole-body gamma exposure of rats by fractionated doses (5 × 2 Gy) resulted in hyperlipidemia and increased serum levels of TC, TG, and LDL-C, together with the significant decrease in HDL-C. The GSH content and SOD activity were decreased, as well as MDA, creatinine kinase-MB (CK-MB), and cardiac troponin I (cTnI). In addition, the histopathologic changes were observed in the heart tissue. In rats supplemented with RJ (250 mg/kg/day), these changes were reduced. Furthermore, RJ may improve the symptoms of oral mucositis and shorten the healing time [280,281]. Rafat et al. [248] also demonstrated the radioprotective effect of RJ. RJ prevented radiation-induced apoptosis in human peripheral blood leukocytes. The blood samples were taken on days 0, 4, 7, and 14 from healthy male volunteers that received a 1000 mg RJ capsule per day for 14 consecutive days. Samples were then exposed to the 4 Gy X-ray. RJ modified the radiation-induced apoptosis of peripheral blood leukocytes, likely through its antioxidant properties and free radical scavenging ability. RJ-mediated protection against the mutagenic effect of Adriamycin and gamma radiation was investigated by El-Fiky et al. [282]. The study showed that pretreatment with RJ reduces DNA fragmentation at two days after gamma radiation at a level *p* < 0.01, reaching *p* < 0.001 at days 4, 7, and 14 after exposure. However, the effects of RJ were not significant in the combined treatment (Adriamycin plus gamma radiation), except for the total number of structural aberrations at day 2 (changes were decreased at *p* < 0.05). Fatmawati et al. [283] reported the protective effect of RJ against UV radiation in Wistar rats exposed to 40 Watt UV-B lamps for 2 h daily during two weeks. RJ was applied as a cream (at concentrations of 2.5%, 5%, and 10%) and protected the skin from UV rays. RJ reduced inflammatory processes and strengthened the antioxidant defense. The highest dose of RJ significantly increased Nrf2 levels and decreased the expression of NF-κB and TNF-α.

The effects of RJ and 10-HDA on UVB-induced photoaging were tested by measuring procollagen type I, TGF-β1, and MMP-1. In UVB-irradiated human skin fibroblasts treated with RJ and 10-HDA, procollagen type I and TGF-β1 were increased, whereas changes in MMP-1 were not observed. It was suggested that potentially RJ could be effective in skin protection from UVB-induced photoaging through enhanced collagen production [117,284].

Apart from chemotherapy and radiation, previous studies reported the in vivo protective and antioxidative roles of RJ against CCl4, oxymetholone, paracetamol, celecoxib, and fumonisin-induced liver and kidney injury, without identifying the responsible components [270,285,286,287,288,289,290,291].

### 5.12. Antimicrobial Activity of Royal Jelly

Due to the huge problem of the rising resistance of microorganisms to known antibiotics, there is an urgent need for novel antibiotics with the advanced mechanisms of action on which microorganisms cannot develop resistance. In this decade, molds and bacteria have been investigated as the source of new antibiotics. Insects only recently became interesting to scientists as fat tissue and other insect cells produce numerous antimicrobial peptides that can be found in their hemolymph. Antimicrobial peptides (AMPs) are also present in RJ [48,63,68,292,293,294,295,296,297,298,299,300]. In addition to antibacterial activity, these peptides show antiviral, antitumoral, immunoregulatory, and hepatoprotective effects as well. The cationic AMPs interact with the negatively charged membranes of various microorganisms and change their membrane electrochemical potential, which ultimately ends in bacterial death due to the disturbance of membrane integrity. Some examples of AMPs are royalisin, jelleines, and aspimin which are present in RJ in small amounts [293]. Peptides present in insects impact bacteria through several mechanisms: (i) creating peptide monomers that join and form big transmembraneous channels in the bacterial wall, and (ii) creating peptides that act as detergents and destroy the bacterial cell wall. Based on the spectrum of activity, the AMPs can be grouped into three categories: (i) peptides that are effective only against bacteria and not towards normal cells of mammals and fungi (selective peptides), (ii) peptides that are effective against bacteria, but also against normal mammalian cells and potentially fungi, and (iii) peptides that are effective exclusively against fungi [294]. The key AMPs found in RJ are defensins, the cysteine-rich (cationic) peptides, such as royalisin. Royalisin is an amphipathic peptide that makes up to 6.5% of the total protein content in RJ. The primary structure of royalisin is composed of 51 amino acids, of which six cysteines form three disulfide bonds [68,295]. These bonds are important for the high stability of royalisin at a low pH and high temperature. The molecular weight of the protein is 5.5 kDa. In addition to royalisin, 10-HDA contributes to the antibacterial activity of RJ [68]. 10-HDA showed prominent bactericidal activity towards animal- and human-specific pathogens, including several strains of *Staphylococcus* (*S. aureus*, *S. alactolyticus*, *S. intermedius B*, *S. xylosus*, and *S. cholearasuis*), *Vibrio parahaemolyticus*, and hemolytic *Escherichia coli* [68]. The antibacterial activity of royalisin and 10-HDA have been reported in both Gram-positive and Gram-negative species of bacteria [48,63,68,292,293,294,295]. These two antibacterial components are important for bees themselves.

Using reverse-phase HPLC 4, Fontana et al. [296] have isolated Jelleine-I-IV, yet another antibacterial peptide from RJ. Jelleine-I-III were effective against yeast, Gram-positive, and Gram-negative bacterial species while Jelleine-IV did not show antibacterial activity. The primary structure of jelleines do not show similarity with other antimicrobial peptides produced by honeybees such as hymenoptaecin, abaecin, apidaecin, and royalisin. Han et al. [297] have studied the antibacterial activities of Jelleine-II and artificially phosphorylated Jelleine-II in one threonine residue against *Paenibacillus larvae*, *Staphylococcus aureus*, *Bacilus subtilis*, *Pseudomonas aeruginosa*, and *Escherichia coli*. The native form of the Jelleine-II showed antibacterial properties, whereas the activity of the phosphorylated form was markedly decreased. It was concluded that phosphorylation adds the negative charge into the peptide, decreasing the antibacterial function. The hydrophobic residues present in jelleines and royalisin are crucial for the observed antimicrobial properties as they modify the functions of bacterial membranes. On the contrary, the halogenation of jellines increased the binding affinity to the bacterial wall and improved the antimicrobial and antibiofilm activity 1- to 8-fold. The proteolytic stability was also improved by 10- to 100-fold. The halogenation of chlorine-, bromine-, and iodine-jelleine-I was more effective than fluorine-jelleine-I [292,298,299]. The antibacterial activity of halogenated derivatives of jelleine-1 (applied at 1 μM to 256 μM) was confirmed against *Staphylococcus aureus*, *Bacilus subtilis*, *Staphylococcus epidermidis*, *Escherichia coli*, *Pseudomonas aeruginosa*, *Klebsiella influenza*, and *Cronobacter sakazakii* at 10^5^ to 10^6^ CFU/mL. The apolipophorin III-like protein from RJ also contributes to its antimicrobial activity. The apolipophorin III-like protein is a lipid-binding protein that binds to the components of the bacterial cell wall. The antibacterial effect of the glucose oxidase present in RJ also needs to be emphasized. This enzyme catalyzes the oxidation of glucose into hydrogen peroxide which shows antimicrobial activity.

In addition to AMPs, the antimicrobial properties of RJ could be assigned to royalactin (MRJP1). MRJPs demonstrate strong antimicrobial and bactericidal activities even against the most drug-resistant bacterial strains such as methicillin-resistant *Staphylococcus aureus*, *Pseudomonas aeruginosa*, and *Klebsiella pneumoniae*, vancomycin-resistant *Enterococci*, as well as extended-spectrum β-lactamase-producing *Proteus mirabilis* and Escherichia coli [48,63,296,300]. Besides being antibacterial, MRJP1 also has antifungal and immuno-stimulatory properties. Due to the negatively charged surface, even at a neutral pH, the oligomer form of MRJP1 (280–420 kDa), called apalbumin, has better storage stability and resistance to heat than the monomeric form. The antibacterial activity of MRJP2 was also reported. The effect on *Paenibacillus larvae* was likely due to the presence of the peptides apidaecin and hymenoptaecin [48,295]. MRJP1 and MRJP2 have a different pattern of glycosylation [295,300,301]. At least for *Paenibacillus larvae*, the glycosylation may affect the antimicrobial activity of MRJP2 [295]. In yet another study, glps, the glycoprotein fraction isolated from honey, was capable of permeabilizing and agglutinating the bacterial membrane. Mass spectroscopy revealed that glps resembles the MRJP1 precursor-harboring jelleins 1, 2, and 4. The authors suggested that high-mannose N-glycans mediate the lectin-like effect of MRJP1, while jelleins contribute to membrane permeabilization [301]. Interestingly, exosome-like extracellular vesicles (EVs) have been found in honey. These vesicles contain MRJP1, defensin-1, and jellein-3 and show antibacterial and antibiofilm activity against oral streptococci which indicates their potential in the prevention of dental caries [302,303]. In closure, the MRJPs demonstrate powerful antimicrobial and bactericidal activities, even against the highly resistant strains such as MRSA, *Pseudomonas aeruginosa*, *Klebsiella pneumoniae*, vancomycin-resistant Enterococci, as well as *Proteus mirabilis* and *Escherichia coli* that produce extended-spectrum beta lactamase [63,300].

The phenolic components of RJ are also important for its antimicrobial activity via various mechanisms. The main components of RJ that contribute to antioxidant and antimicrobial activity are flavanones (naringenin, naringin, hesperetin, isosacuranetin, and pinocembrin), flavones (chrysin, luteolin glucoside, acacetin, apigenin, and its glycoside), flavonol (quercetin, kaempherol, galangin, fisetin, and isorhamnetin), and isoflavones [62,63]. Phenolic components may cross the microbial cellular membranes and impair membrane fluidity. Interactions with membrane enzymes and proteins may result in the flow of protons in the opposite direction and disturb cellular activities such as energy production, membrane transport, and other metabolic regulatory functions. Phenolic compounds also inhibit the synthesis of DNA and RNA and protein translation, and may suppress microbial virulence factors [304,305]. They can also degrade the cell wall and cytoplasmic membranes, induce the leakage of the cellular content, and change fatty acid and phospholipid constituents. Furthermore, they act synergistically with antibiotics, thus increasing their effectiveness and reducing the dose. Besides showing their antibacterial effect, phenolic components also inhibit the growth of protozoa, food-related pathogens, and fungi. The antifungal properties of RJ have been shown against *Aspergillus fumigants*, *Aspergillus niger*, *Candida albicans*, and *Syncephalastrum racemosum* [304]. Finally, high concentrations of RJ are effective against *Pseudomonas Aeruginosa* which may be important when using RJ in the wound healing process [304].

Furthermore, RJ can inhibit the growth of *Trypanosoma cruzi*, a parasitic protozoan. Its activity has been confirmed for another parasite, dysenteric amoeba. It effects flu viruses as well. Multiple small doses of royal jelly increase resistance to stress in laboratory animals while high doses are lethal. Antibacterial and bactericidal properties have been proven against the species *Bacillus alvei*, *Streptococcus haem*, *Staphylococcus aureus*, *Proteus vulgaris*, *Micrococus*, and *Listeria monocytogene* [101,292,293,294,295,296,297,298,299,300,306].

It is interesting that RJ was used as an adjuvant for the HIV-1 multi-epitope vaccine candidate. It was supposed that the components of RJ act as immunopotentiators and promote the Th1 immune response. RJ applied alone stimulates cellular and humoral immune responses, but shows synergistic effects when combined with alum, thus improving the immunologic parameters such as the production of total antibodies and IgM, IgG1, IgG2a, and IgG2b isotypes. As an adjuvant, RJ stimulated lymphocyte proliferation and the release of IFN-γ which further activated TCD8+ and TCD4+ lymphocytes in the fight against viral infections. The induction of the Th1 response is crucial for combating viral infections such as HIV-1. 10-HDA was the main component of RJ for the production of the Th1 cytokine pattern in human monocyte-derived dendritic cells. It seems that T cells stimulated with RJ may stimulate B lymphocytes to produce antibodies. RJ can induce the production of all isotypes of antibodies, i.e., the poly-isotypic humoral immune response, indicating a better biological response [307]. In addition to RJ, purified MRJPs of *Apis mellifera* also showed powerful antiviral activity against HCV, HIV, and SARS-CoV-2 [305,306,307,308,309,310,311]. The antiviral effects of MRJP2 and its isoform X1 have been studied by the computational tools and 3D structure of the SARS-CoV-2 proteins (from the Protein Data Bank) which includes papain-like protease (non-structural protein nsp3), 3CL protease (nsp5), the nsp5-inhibitor complex, and RNA replicase (nsp9). The RNA-dependent RNA polymerase (nsp12)-cofactor (nsp7-nsp8) complex and the methyltransferase (nsp16)-cofactor (nsp10) complex were also involved. MRJP2 and MRJP2 X1 exerted inhibitory effects on SARS-CoV-2 non-structural proteins (main and papain proteases, RNA-dependent RNA polymerase, RNA replicase, and methyltransferase). The obtained results suggest that functional food proteins could be effective in the prevention of the SARS-CoV-2 cell-attachment. The antiviral action was assigned to their sialidase activity and ability to interact with the binding sites of the angiotensin converting enzyme-2 (ACE2) on the viral spike. The docking studies indicated that the inhibitory activity of MRJP2 and MRJP2 isoform X1 was based on their ability to bind to the active sites or the cofactor-binding site on the viral non-structural protein nsp3, nsp5, nsp9, nsp12, and nsp16. Therefore, the RJ proteins could be effective in the prevention of the severe complications of the disease in the lungs, such as hypoxia, and in the prevention of inflammation and thrombosis. In conclusion, RJ proteins have an important role both in the prevention of viral replication and in the prevention of life-threatening complications [308].

### 5.13. Wound-Healing Activity of Royal Jelly

Wound healing is a programmed biological process involving inflammation and cell proliferation, differentiation, and migration, as well as numerous intracellular and extracellular components, like inflammatory mediators (cytokines and chemokines), growth factors, and ATP, among others [312]. In the process of wound healing, the proliferation and differentiation of fibroblasts are critical for the formation of the granulation tissue and connection of wound edges. The proliferation and migration of keratinocytes from the wound enables re-epithelialization, protecting the wound from infection and desiccation. Macrophages are also important for wound healing by dampening inflammation. They produce various regulators that promote an inflammatory response and remove bacteria and necrotic tissue through the phagocytic activity [36,313,314,315,316,317].

In general, it has been shown that RJ and its components promote wound healing at both animal and cellular levels [318,319,320,321,322,323,324,325,326]. Some components of RJ show anti-inflammatory effects (SA, 10-HDA, and 10-HDAA), some components promote proliferation and migration (MRJPs, especially MRJP2, MRJP3, and MRJP7), whereas some components stimulate regenerative activities (royalactin, royalisin, and 10-HDA) [313,314,315,316,317,318,319,320,321,322,323,324,325].

In streptozotocin-induced diabetic rats, the wound-healing activity of RJ was attributed to its royalisin-mediated antimicrobial effects, the anti-inflammatory action, and the ability to reduce exudation and collagen formation in granulation tissue [313,314]. Kohno et al. [36] suggested that the anti-inflammatory actions of RJ on the inhibition of the proinflammatory cytokine are released by activated macrophages as well as the scavenging activity. In streptozotocin-induced diabetic rats, RJ also shortened the time required for the recovery of desquamated skin lesions [313,314]. El-Gayar et al. [314] have shown the wound healing effect of RJ in a rat model of an MRSA skin infection. In another study, Lin et al. [315] have tested the wound healing efficacy of different RJ samples in excisional full-thickness wounds. The most potent was *Castanea mollissima* Bl RJ. This type of RJ accelerated wound repair by promoting the proliferative and migratory abilities of keratinocytes by 50.9% and 14.9%, respectively. Furthermore, *Castanea mollissima* Bl RJ inhibited the production of nitric oxide by 46.2%, and promoted cell growth by increasing the levels of TGF-β by 44.7%. On the contrary, for *Brassica napus* L., RJ ameliorated inflammation by reducing the secretion of TNF-α by 21.3%. Hence, in complicated wound models, such as MRSA skin infections, diabetic foot ulcers, and infected cutaneous wounds, RJ could be considered as an option for wound care. Further research should explore in more details the range of applications of a particular type of RJ in chronic wounds, and identify specific components of RJ that promote wound healing.

Patients undergoing radiotherapy and chemoradiotherapy for the treatment of head and neck cancers are often faced with oral mucositis, usually accompanied with the ulcerations of the oral mucosa. Oral mucositis develops in 15–40% of patients treated with standard chemotherapy, and in all patients receiving radiation therapy as part of the head and neck cancer treatment, oral mucositis occurs at some degree. These patients also may experience pain, dysphonia, and dysphagia. Tissues with the most prominent mitotic activity, such as oral mucosa, are rapidly affected by radiation. A quick response is also characteristic for chemotherapeutics such as cisplatin, S-1, nedaplatin, and docetaxel. The level of normal tissue damage following chemoradiotherapy depends on the dose, fractionation schedule, and volume of the treated tissue. A beneficial effect of RJ in reducing chemoradiotherapy-induced oral mucositis in head and neck cancer patients and animal models was also observed [326,327,328,329,330,331]. For example, the application of RJ films (10% and 30%) accelerated recovery from a 5-FU-induced injury by showing the free radical-scavenging activity and by reducing the myeloperoxidase activity and production of pro-inflammatory cytokines in hamsters [327,329,330]. Erdem and Güngörmüş [280] performed studies in patients undergoing radiotherapy and chemotherapy. They reported a significantly shorter mean time for the resolution of oral mucositis in the RJ group. These data suggest that RJ possesses a healing effect against the chemotherapy- and radiotherapy-induced oral mucositis that is largely mediated by the anti-inflammatory and anti-oxidative activities of RJ [328,331].

Chen et al. [332] have found that MRJPs from RJ could induce the proliferation of human cells and could replace the fetal bovine serum in culture media. The growth-promoting activity was confirmed in many cell lines, including human embryonic lung fibroblasts (HFL-I) [111], human monocytes (U-937 and HB4C5) [21], human lymphoid cells (U-937, THP-1, U-M, HB4C5, and HF10B4) [33,246,332], human keratinocytes [26], human microvascular endothelial cells (HMEC-1) [115], the primary culture of rat hepatic cells [26,27], rat small intestine epithelial cells [74], murine fibroblasts (NIH-3T3) [29], stem cells [30], monocyte/macrophage-like cells *RAW 264.7* [324] and monkey kidney epithelial cells [31], and Tn-5B1-4 insect cells [112].

The treatment of the human embryonic lung fibroblast cell line (HFL-I) with the MRJP mixture was confirmed to promote proliferation and reduce senescence [111]. Moreover, cells that were grown in MRJPs-containing medium had longer telomeres compared to cells grown in media without MRJPs. The molecular mechanisms underlying the anti-senescence effect of MRJPs were associated with the upregulation of *SOD1* and the downregulation of *mTOR*, catenin beta like-1 (*CTNNB*), and *TP53* at the gene level [111]. A similar effect was observed for recombinant MRJPs 1–7 which increased the viability of NIH-3T3 cells (murine fibroblasts) by protecting them against oxidative stress-induced apoptosis [29]. It is interesting that oligomeric MRJP1 stimulated the proliferation of human monocytes (U-937 and HB4C5 cell lines), whereas the monomeric form (royalactin) failed to stimulate the proliferation of these cells. Oligomeric MRJP1 also stimulated the growth of Jurkat and IEC-6 cells, while monomeric MRJP1 promoted the proliferation of human lymphoid cell lines, such as U-937, THP-1, U-M, HB4C5, and HF10B4 [31,332]. Furthermore, the monomeric MRJP1 may act as a pluripotency factor and activate the pluripotent gene regulatory network that enables the self-renewal of mouse embryonic stem cells [44]. MRJP2, MRJP3, and MRJP7 have the potential to promote wound healing by inducing the proliferation and migration of human epidermal keratinocytes [26]. The carboxyl-terminal penta-peptide repeats (TPRs) of MRJP3 stimulated the growth and wound healing activity of THP-1 and monkey kidney epithelial cells (Vero) [31].

### 5.14. Antiallergic Effect of Royal Jelly

An antiallergic effect of RJ has been confirmed in the mice model [37]. The antiallergic activity of RJ was attributed to MRJP3 which inhibited the production of IL-4 in mice splenocytes immunized with ovalbumin. MRJP3 not only inhibited IL-4, the key factor in immunoglobulin class switching to IgE in B cells, but also inhibited the production of IL-2 and IFN-γ by T cells and inhibited the proliferation of T cells. The potent immunoregulatory effect of MRJP3 regarding the inhibition of IgE and IgG1 production in immunized mice could be of clinical significance in designing MRJP3-based peptides with antiallergic properties.

In contrast to MRJP3, MRJP1 and MRJP2 can cause allergic reactions. They interacted with immunoglobulin E (IgE) of sera from patients exhibiting an RJ allergy [31,37]. IgE-binding was dependent on the glycosylation degree of MRJP1 and MRJP2.

Oka et al. [333] suggested that the suppression of an allergic reaction by RJ is related to the maintenance of macrophage function and the increased Th1/Th2 response. They have found that RJ maintains the GSH levels, increases the production of IL-12 p40 mRNA and NO expression, and reduces the production of E2 prostaglandin in the macrophage cells of mice immunized with 2, 4-dinitrophenylated keyhole limpet hemocyanin (DNP-KLH). Therefore, they concluded that RJ suppresses the production of allergen-specific IgE antibodies and histamine release, restores the macrophage function, and improves the Th1/Th2 cell response. The GSH levels may affect the response of the Th cells. The GSH increase in macrophages is a key factor for increasing the IL-12 levels and streamlining the Th1 response, which further favors a decrease in prostaglandin E2. It has been shown that prostaglandin E2 increases the suppression Th1 response and increases IgE production. In addition, it has been reported that increased NO levels suppress the degranulation of mast cells. Taken together, these findings indicate that RJ can protect from allergies or alleviate the symptoms of allergy.

RJ also reduced the development of skin lesions similar to atopic dermatitis in NC/Nga mice treated with picryl chloride [334]. The oral administration of RJ decreased the total skin weight, reduced the hypertrophy and hyperkeratosis of the epidermis, and inhibited the infiltration of inflammatory cells. The main mechanism of these effects was based on the reduced production of trinitrophenyl-conjugated keyhole limpet hemocyanin (TNP-KLH) TNP-specific IFN-γ and enhanced iNOS expression [334]. In another study, the oral intake of RJ reduced the number of splenic autoreactive B cells, serum IL-10 levels, and anti ssDNA, dsDNA, and erythrocyte autoantibodies in systemic lupus erythematosus (SLE)-prone New Zealand Black × New Zealand White F1 mice [335]. The positive effect of RJ on SLE was also confirmed in children taking 2 g of RJ for 3 months. The improvement of the disease severity was evident through the increased number of CD4+ and CD8+ regulatory T cells and a reduced number of apoptotic CD4+ T cells [336]. A potential immunoregulatory effect of RJ against Graves’ disease was also demonstrated. Lymphocytes obtained from the patients with Graves’ disease were incubated with RJ. This treatment reduced the level of TNF-α and increased the levels of IFN-γ, and shifted the Th1/Th2 cytokine ratios towards Th1 dominance [337].

Guendouz et al. [338] have demonstrated that RJ may help in the prevention of systemic and anaphylactic responses in a murine model of a cow’s milk allergy. RJ was applied at doses of 0.5, 1, and 1.5 g/kg during seven days and significantly inhibited the production of serum IgG (31.15–43.78%) and IgE (64.28–66.6%) against β-lactoglobulin and reduced the plasma histamine level after the β-lactoglobulin challenge (66.62–67.36%). In addition, RJ reduced intestinal dysfunction by abolishing the sensitization-induced secretory response (70.73–72.23%). It also prevented tissue damage and increased the length of jejunal villi by 44.32–59.01%. These effects may be related to its inhibitory effects on the degranulation of mast cells [338].

It is also interesting to note that protease-treated RJ can promote an antigen-specific mucosal IgA response by enhancing the uptake of antigens by M cells, increasing the efficacy of the immune response of the intestinal mucosa [339].

### 5.15. Allergic Reactions as Side Effects of RJ Application

RJ is broadly used as a dietary supplement and for cosmetic purposes, and is generally considered as relatively safe. However, various proteins present in RJ can cause side effects such as allergic reactions. A mild-to-severe allergic reactions may include skin rashes, eczema, contact dermatitis, allergic rhinitis, conjunctivitis, minor gastrointestinal problems, hemorrhagic colitis, acute asthma, bronchospasm, anaphylactic shock, and in some cases, even death [340]. Considering the possibility of unwanted effects, the consumption of RJ is not recommended for expectant mothers, lactating women, and individuals prone to allergic reactions. Allergic reactions may occur after the first intake of RJ, suggesting the cross-reactivity with other allergens, especially mite and arthropod allergens. According to Hata et al. [340], for those with a history of allergic diseases, such as atopic dermatitis, asthma, and allergic rhinitis, caution is needed when consuming RJ products because of the potential cross-reactivity. In patients with atopic dermatitis who had never consumed RJ, RJ-binding IgE antibodies have been found. The cross-reactivity with RJ has been demonstrated for the European and American house dust mite, snow crab, edible crab, German cockroach, and honeybee venom. Most RJ proteins (app. 90%) are members of the MRJPs, and MRJP1, MRJP2, and MRJP3 are identified as allergens [340,341,342,343]. Li et al. [344] suggested that MRJP3 is the main cause of anaphylaxis and cross-reactivity with honeycomb, and that patients with an allergic reaction to RJ should avoid other bee products.

## 6. Scientific Claims about Effectiveness of RJ to Human Health According to PASSCLAIM Classification

The main objectives of the PASSCLAIM project (Process for the Assessment of Scientific Support for Claims on Foods) were to define the group of widely applicable criteria for the scientific explanation of health claims about the effectiveness of food. These criteria are considered powerful indicators for the assessment of the quality of data that support claims on health benefits of foods. According to EU Regulation No 1924/2006, different health claims about the benefits of RJ can be derived. According to the PASSCLAIM project classification of the International Life Science Institute (ILSI), the claims are grouped into the following themed areas:

(1) Diet and cardiovascular diseases,
(2) Bone health and osteoporosis,
(3) Physical strength and fitness,
(4) The regulation of body mass, insulin sensitivity, and risk of diabetes,
(5) Diet and cancer,
(6) Mental health and effectiveness,
(7) Bowel health, digestion, and immunity.

The health claims of RJ are supported for categories of diet and cardiovascular diseases, physical strength and fitness, diet and cancer, and mental health and effectiveness. In particular, a preventive intake of RJ at doses of 100 to 200 mg daily may enhance the health of the cardiovascular system by decreasing blood fats and cholesterol, the main cardiovascular risk factors [345]. RJ should be taken for 30 days and followed by 2-to-3-week break. After a break, it is recommended to restart with lower doses. Regarding physical strength, the intake of RJ may increase physical strength, fitness, and work ability, especially in the elderly (anti-age effect). Taking RJ also may decrease one’s risk of cancer and enhance their mental status and physical effectiveness [177,191,197,346,347]. Again, these effects are particularly important in the elderly. Some of the above-mentioned effects are shown in Table 3 and Table 4.

## 7. Need for Standardization of Important Biologically Active Compounds and Determination of Validity and Quality of Products with RJ

RJ has been used for a long time. The first recommendation for its use in human therapy dates from 1922 in France. Since then, RJ has become the subject of numerous studies. In many European and world countries, the medicinal use of RJ is monitored by the relevant ministries of health and regulatory bodies. In an effort to ensure the safe usage of RJ as a medicinal product, it is important to guarantee the presence of specific compounds important for its health benefits and to ensure quality production. Hence, the chemical composition and quality of RJ should be analyzed continuously. The basic nutritional requirements of RJ that must be ensured include water (min 62.0%–max 68.5%), lipids (min 2%–max 8%), 10-HDA (min 1.4%), proteins (min 11%–max 18%), total carbohydrates (min 7%–max 18%), and individual carbohydrates [fructose (2.3–7.6%), glucose, (2.9–8.1%), sucrose (<0.1–2.1%), maltose, and maltotriose (0.0–1.0%)] [7].

Various factors may affect the production and chemical composition of RJ, including the post-grafting period, harvest time, diet of the bee colonies, and seasonal variations [20,348]. Regarding the yield and nutritional composition, the optimal time to harvest RJ is after 72 h of grafting. On the contrary, the minimum yield is observed after 24 h. The lowest moisture and the highest content of crude proteins, ash, fructose, and glucose were obtained 72 h after harvesting. However, RJ collected after 24 h contained the highest lipid concentration. The content of 10-HDA in RJ also varies. It is dependent on the season of harvesting. In one study, the amount of 10-HDA was monitored from April to August. The highest concentration of 10-HDA was measured in June (1.84%), whereas the lowest amount was found in April (1.03%) [349]. Furthermore, a change of acidity was observed over time. These findings indicate the complexity of the RJ content. Accordingly, a comprehensive understanding is needed to produce RJ as a medical product with a defined chemical composition [350]. Furthermore, it is hard to gather data obtained in different studies due to the lack of homogeneity of the original materials with different sampling methods and production conditions. Additional sources of variability are various experimental conditions as well as the diversity of the analytical methods applied. Of note, it has been reported that pesticides may affect the quality and quantity of RJ. Milone et al. [351] observed differences in the metabolome, proteome, and phytosterol profile of RJ produced by colonies exposed to the multi-pesticide pollen. Some of the essential nutrients, such as 24-methylenecholesterol, MRJPs, and 10-HDA, were significantly reduced. Additionally, the quantity of RJ provided per queen was lower in colonies exposed to pesticides. According to the United States Environmental Protection Agency (USEPA) risk assessment guidelines, exposure to pesticides is app. 100 times lower in RJ compared to pollen and nectar (USEPA, 2014).

Milone et al. [351] also observed a reduction in the average RJ per cell between control and pesticide-exposed colonies (283 mg vs. 198 mg, respectively), but the difference was not statistically significant. The complete knowledge of the RJ components is essential for defining the standard composition and assessment of the quality of marketed products containing RJ [349,350,351,352,353]. Some countries, like Switzerland, Bulgaria, Brazil, and Uruguay, have defined national standards for RJ. Also, the difference between fresh RJ and lyophilized samples needs to be clearly stated. In general, the lyophilized RJ contains <5% water, 27–41% proteins, 22–31% carbohydrates, and 15–30% fat [353,354,355]. The analytical data indicate that the exposure to the temperature of 4 °C does not change its composition. In addition, the storage of frozen RJ prevents the decomposition of biologically active proteins. Hence, RJ needs to be frozen immediately after collection to preserve the bioactive compounds [352]. Some important differences in RJ composition are shown in Table 1.

The main nutritional value of dietary proteins, as well as RJ, is determined by the amino acid composition. Therefore, monitoring changes in the amino acid composition can be an effective method of assessing the quality of RJ. One of the most powerful tools for analyzing amino acid composition in RJ is ultra-performance liquid chromatography (UPLC). By using UPLC, it was determined that the average levels of free amino acids (FAAs) and total amino acids (TAA) in fresh RJ were 9.21 and 111.27 mg/g, respectively. The most abundant FAAs in RJ are proline, glutamine, lysine, and glutamic acid, whereas the most common TAAs are asparagine, glutamine, leucine, and lysine. Although the concentrations of the majority of the FAAs and TAAs did not change significantly during the storage, the amount of total methionine and free glutamine decreased over time. Hence, the authors suggested that the levels of total methionine and free glutamine may be considered as a marker of RJ quality. In another study, the concentrations of proline and lysine increased during the first three months, and then decreased after 6–10 months, indicating proteolytic activity over time.

Besides proteins, 10-HDA and 10-HDAA are specific components of RJ [5,6,7,13,14,353,356]. HDA is considered as the best marker of RJ quality. Moreover, it may be used as an indicator of forgery. Based on the standards of the International Organization for Standardization (ISO), the total amount of 10-HDA should be more than 1.4% to meet the quality control parameters (ISO, 2016). However, the composition of RJ and amounts of 10-HDA may vary widely. In two RJ samples of different origins (France and Thailand) stored at room temperature for one year, a reduction of 0.4% and 0.6% of 10-HDA was observed. In addition, 10-HDA is difficult to use as a measure of freshness as its values differ in fresh RJ. In general, the RJ of a European origin contains lower levels of 10-HDA. Hence, it is considered that the total concentration of fatty acids is a better indicator of freshness than 10-HDA itself [353,356]. Although the specification of RJ products requires the content of 10-HDA, which is chemically stable and unique to RJ, a potential limitation of using 10-HDA is that its levels in RJ decline during the production process. Furthermore, there is a possibility of manipulation by adding RJ rich in 10-HDA. On the contrary, amounts of apisin are fairly constant among different RJ samples. Therefore, it is likely that apisin could be used as an indicator for the evaluation of RJ quality. MRJP1, the main constituent of apisin, plays an essential role not only in the assessment of RJ quality, but also in the determination of RJ freshness [349,350,351,352,353]. However, properties of MRJP1 are dependent on storage conditions. Storage above 4 °C induces the degradation of MRJP1 due to its lytic activity. Furthermore, Mureşan and Buttstedt [357] investigated the stability of MRJPs in different pH environments and found a direct correlation between the pH levels and protein stability. In addition, carbohydrates from RJ, including trehalose, maltose, erlose, melibiose, ribose, gentiobiose, isomaltose, raffinose, and melezitose, are important for bee products’ authenticity. The carbohydrate analysis also may help in the detection of forged RJ [353,354,358,359]. The ISO recommendation for the determination of the carbohydrate constituent is liquid chromatographic analysis (the reference method), titration method gas chromatographic analysis, and the Hydrophilic Interaction Liquid Chromatography–tandem Mass Spectrometry (HILIC-MS/MS) method [13,358,359,360]. Furthermore, the authenticity of RJ production can be determined by measuring the ratio of stable C and N isotopes. Geographical authenticity can be determined by pollen analysis [348,350]. In particular, the 87Sr/86Sr ratio indicates the geographic origin [361,362]. The levels of ADP, ATP, and AMP are used as indicators of the freshness of RJ as their levels are high in fresh samples. Taken together, the key parameters in the estimation of RJ quality are organoleptic, physical, and chemical properties and its composition as follows: (i) the water content in fresh and frozen samples of RJ; (ii) the concentration of total proteins and free amino acids; (iii) the concentration of carbohydrates; (iv) the concentration of lipids; total fatty acids and free acids; (v) 10-HDA; (vi) minerals; (vii) acidity; (viii) sediment analysis; (ix) furosine; and (xj) potential contamination. In addition, the freshness of RJ or its products may be estimated by measuring the activity of glucose oxidase, furosine, or superoxide dismutase [353,354,355,356]. Therefore, RJ and RJ-containing products need to be prepared according to detailed sanitary and hygienic protocols and monitored by trained experts to maintain the beneficial effects.

## 8. Closing Remarks

The biological characteristics of RJ, serving as the base for its potential use, are the following: its biostimulatory effect—the ability to increase tissue respiration, oxidative phosphorylation, exchange of matter, energy and fitness, increased resistance to stress and illnesses, the maintenance and strengthening of the hair, skin, and nails, the regeneration of cells and tissues, decrease in cholesterol levels, positive effect on the vascular system, stabilization of blood pressure, increase in memory (acetyl choline neurotransmitter), stimulation of the immune system, increased appetite, digestion regulation, hormonal balance maintenance, ease of menstrual symptoms, help with impotence and frigidity, fatigue and tiredness relief, positive effect on arthritis, anemia, muscle dystrophy, and neurodegenerative diseases, and pro-regenerative, anti-inflammatory, antiviral, and antibiotic effects (Table 3 and Table 4).

It is interesting that exosome-like extracellular vesicles (EVs) found in honey from *Apis mellifera* contain MRJP1, defensin-1, and jellein-3 as intravesicular cargo, which indicates that honey-derived EVs could represent innovative approaches for preventing dental caries. RJ also contains a considerable number of extracellular vesicles (EVs). The molecular analysis of RJEV confirmed the presence of exosomal markers such as CD63 and syntenin, and cargo molecules MRJP1, defensin-1, and jellein-3. RJEV can modulate the differentiation and secretome of mesenchymal stem cells (MSCs), as well as reduce LPS-induced inflammation in macrophages by blocking the MAPK pathway. The most interesting for wound healing is the secretory profile of MSCs, which secrete various proteins, especially increasing the level of IGF, HGF, and VEGF, but not FGF acting on pro-angiogenic and promigratory effects in wound healing, while having minor effects on scars and fibrosis. Overall, RJEV exhibits a significant antibacterial effect and promotes wound healing by modulating underlying cellular responses. The antibacterial and antifungal activities of MRJPs and jelleines are achieved through the interactions of positively charged amino acids with the hydrophobic residues in bacterial cell membranes. Furthermore, it is important to highlight that RJ and its proteins prevent the replication of the SARS-CoV-2 virus and severe complications of the disease. It is possible that isolating EVs from raw RJ will reduce the high complexity of RJ components, enable standardization and quality control, and thus bring the application of natural nano-therapy closer to the clinic.

Regarding potential side effects, RJ can cause contact dermatitis and anaphylaxis in some people due to the presence of many proteins. The usage of RJ is limited in diseases such as bronchial asthma exacerbations (RJ is astringent and can lead to bronchiectasis), Addison disease, adrenal gland diseases, and tumors in the acute phase. The estrogenic and proliferative activities of RJ have been shown in MCF-7. However, the procarcinogenic and estrogenic effects of RJ on other tumors have not been confirmed. On the other hand, RJ is effective against anti-cancer agent-induced toxicities, such as mucositis, fibrosis, cardiotoxicity, intestinal damage, and renal and hepatic dysfunction. In addition, the modulatory effect of RJ on various biological activities, including cell survival, inflammation, and oxidative stress, is closely associated with its effects (Table 3 and Table 4). An overview of the molecular and cellular mechanisms of RJ is shown in Table 5, while the doses of RJ used in some clinical trials are shown in Table 6.

Based on the above-mentioned characteristics of RJ and its effect on human health and quality of life, appropriate methods should be used for the standardization of RJ and RJ-containing products, especially regarding biologically active compounds, to ensure maximal effectiveness for human health. Knowledge about the main biological compounds and their seasonal and geographical variability, variability over time, proper ways of storage, as well as the determination of the safe therapeutic dose, are important factors in the prevention and protection from disease. Finally, knowledge about the medicinal effects of RJ will enable its wider use and will promote RJ production.

## Figures and Tables

**Table 1:** Key components and their content in fresh and lyophilized royal jelly.

Contents	Fresh Royal Jelly (%)	Lyophilized Royal Jelly (%)	References
Water	60–70	<5	
Lipids	3–8	8–19	
10-hydroxy-2-decenoic acid (10-HDA)	>1.4	>3.5	
Proteins	9–18	27–41	
Fructose + glucose + sucrose	7–18	-	
Fructose	3–13	-	
Glucose	4–8	-	[2,3,5,6,7,8,9,10,11,12,13,14,15,16,17,18,19,20,21,22,23]
Sucrose	0.5–2.0	-	
Minerals	0.7–1.5		
Ash (dry weight)	0.8–3.0	2–5	
pH	3.4–4.5	3.4–4.5	
Acidity (mL 0.1N NaOH/g)	3.0–6.0	-	
Furasine (mg/100 g protein)	<50	-	

**Table 2:** Bioactive components of royal jelly and their functional activity. n.d.: Not determined.

Bioactive Component	Fresh Royal Jelly (%)	Lyophilized Royal Jelly (%)	Functional Activities	References
**Proteins**				
MRPJ1 (Alternative name: Royalactin, apalbumin 1, D III)	5.89%		Antimicrobial, antibacterial, antifungal,	[2,3,12,14,18,48,63]
			wound healing,	[3,7,13,26,31,48,57]
			antiproliferative, antioxidant, anti-inflammatory, antitumoral,	[7,13,29,58,68,69,70,71,72,73,74,75,76]
			immunomodulatory,	[13,14,15,17,19,69]
			hypocholesterolemic, anti-hypertensive,	[27,28]
			proliferation of intestinal epithelial cells (IEC-6),	[31]
			increase in lifespan in invertebrates,	[11,26,53]
			proliferation rat hepatocytes,	[26]
			larvae differentiation into queen via epidermal growth factor signaling,	[11,30,43,44,47,48]
			self-renewal of stem cells,	[11,26,30]
			proliferation of human monocytes and Jurkat lymphoid cell	[33,35,44]
MRPJ2 and isoform (Alternative name: Apalbumin 2)	1.41%		Antimicrobial, antibacterial, antifungal, antiviral,	[2,3,12,14,18,48,63]
			wound healing,	[26,31]
			antioxidant, antitumoral,	[29,62,63,64,65,66,67,68]
			anti-allergic,	[31,37]
			hepato-renal protective,	
			promotione of caspase-dependent apoptosis, inhibition of bcl-2 and p53 expression in HepG2 cells	[60,61]
MRPJ3	1.66%		Wound healing,	[26,31]
			anti-allergic,	[31,37]
			anti-aging,	[53]
			anti-inflammatory, antitumoral,	[34,36,69,70,71]
			immunoregulatory effect, modulation of immune responses of T cells, suppression of proinflammatory cytokine secretion, decrease of IL-4, IL-2, and IFN-γ in vitro and anti-OVA IgE and IgG1 in vivo	[13,37]
MRPJ4	0.89%		Antimicrobial	[2,3,12,14,18]
MRPJ7	0.51%		Wound healing	[14,17,19,26]
**Enzymes**				
Glucose oxidase		0.08%	Carbohydrate metabolism, antibacterial	[3,5,15]
Gluco-cerebrosidase			Hydrolysis glucosylceramide	[22,53]
Alpha-glucosidase			Hydrolysis of polysaccharides and oligosaccharides into monomers	[22,53]
**Antimicrobial peptides and proteins**				
Royalisin	0.83%		Antimicrobial, antibacterial, antifungal, inhibition of Gram-positive bacteria through damage to cell walls and cell membranes	[12,13,14,15,16,48,63,68]
Apisimin	0.13%		Antibacterial,	[9,10,12,17,48]
			increases the proliferation and stimulation of human monocytes	[21,33]
Yelleines I-III	0.37%		Antimicrobial, antibacterial, inhibition of yeast, Gram-positive and Gram-negative bacteria, cell degranulation, hemolysis, increase immune response	[13,14,15,16,48,63,68]
Yelleine IV	-		Antimicrobial	[48]
Venom protein 2	-		Protection of larvae from diseases infection	[48,63]
Apolipophorin-III-like protein	0.08%		Antimicrobial, immunoregulatory effect, stimulation of immune response	[48,63]
**Lipids and fatty acids**				
10-hydroxy-2-decenoic (10-HDA),	0.75–3.39%		Immunomodulatory,	[13,67,69,76,77]
			antioxidant,	[7,52,53,62,68]
			antiaging,	[53,67]
			neurotrophic and neurogenesis inductor,	[4,58,68]
			anti-inflammatory functions,	[7,13,58,62,63,68,69]
			antimicrobial, antibacterial,	[48,67,68]
			estrogenic,	[28,58]
			antialergic,	[7]
			antiosteoporetic,	[35,58]
			antitumor activity,	[7,34,60,61]
			activation of TRPA1 and TRPV1 receptors and increased longevity in *C. Elegans*,	[52,53,59]
			anti-ultraviolet B properties and skin protection,	[59]
			decrease of IL-6 production by reducing expression of IκBζ in RAW264 cells,	[48,63,68]
			inhibition of NO production through the inhibition of NF-κB activation	[13,58,69]
10-hydroxydecanoic acid (10-HDAA)	0.78–1.05%		Anti-inflammatory,	[67,68]
			estrogenic,	[28,60]
			activation of TRPA1 and TRPV1 receptors	[52,53,59]
8-hydroxy octanoic acid	0.18–0.39%		Varroa-repellent	[3,35,67,68]
3-hydroxydecanoic acid	0.05–0.09%		Antifungal	[3,5,35,67,68]
3,10-dihydroxydecanoic acid	0.26–0.46%		Immunomodulatory, stimulation of dendritic cell, differentiation, inhibition of the proliferation of allogeneic T cells and dendritic cell-dependent production of IL-2	[22,23,35]
9-hydroxy-2-decenoic acid	0.07–0.15%		Signal components (pheromone) of honeybee queen	[35,49,50]
1,10-decanedioic acid (sebacic)	0.15–0.24%		Estrogenic,	[28,53]
			anti-inflammatory,	[36,68,69]
			hypotensive	[8,23]
2-Decenedioic	0.18–0.33%	<0.1–3.6%	Anti-inflammatory	[36,68,69]
Phenols	0.24–0.6%	4–10%	Antioxidant	[8,9,10,11,62,63,64,65,66,67]
**Phenolic Acids**				
Ferulic acid	68.42 mg/100 g		Antioxidant, anti-inflammatory, and antiviral properties	[62,63]
Chlorogenic Acid	37.61 mg/100 g			
Caffeic Acid	5.14 mg/100 g			
**Flavonoids**				
Quercetin	16.13 mg/100 g		Anticancer, antioxidant, anti-inflammatory, and antiviral properties, neuroprotective and cardio-protective	[62,63,64,65,66,67]
Naringin	0.47 mg/100 g			
Hesperetin	0.85 mg/100 g			
Galangin	0.51 mg/100 g			
**Waxes**	0.3–0.36%	5–6%	-	
Steroids	0.18–0.24%	3–4%	Effects on collagen synthesis	
24-methylene cholesterol	6.06 mg/lipid		Estrogenic	[2,3,28]
Phospholipids	0.02–0.04%	(0.4–0.8%)	-	
**Carbohydrates**				
Fructose + glucose + sucrose Fructose glucose	7–18% 3–13% 4–8%	90% of the total sugar 2.3–7.6% 2.9–8.1%	Act as an energy source, help in control of blood glucose and insulin metabolism, participate in cholesterol and triglyceride metabolism, and help with fermentation	[2,3,5,6,7,52]
Sucrose	0.5–2.0%	<0.1–3.6%	Increases mental alertness, memory, reaction readiness, attention and the ability to solve mathematical problems, as well as reducing the feeling of fatigue	[2,3,5,6,7,52]
Trehalose, maltose, erlose, melibiose, ribose gentiobiose, isomaltose, raffinose, and melezitose			Modulate glucose homeostasis, reduce bone resorption and inflammation, induce autophagy and alleviate Huntington’s disease, neurodegenerative and cardiometabolic diseases, enhance resistance to oxidative stress	[2,3,5,6,7,52]
**Vitamins**				
Vitamin A	1.10 mg/100 g		Helps form and maintain healthy teeth, skeletal and soft tissue, mucus membranes, and skin, reproduction, immunity, maintenance of the visual system, and epithelial cellular integrity	[2,3,5,6,7]
Vitamin B1	2.06 mg/100 g		Transketolation, metabolism of fats, proteins, and nucleic acids	[2,3,5,6,7]
Vitamin B2	2.77 mg/100 g		Precursor of FMN and FAD	[2,3,5,6,7]
Niacin (B3)	42.42 mg/100 g		Increase HDL	[2,3,5,6,7]
Vitamin B5 (Pantothenic acid)	52.80 mg/100 g		Constituent of coenzyme A, plays a role in breaking down fats and carbohydrates for energy, crucial for red blood cell production, production of sex and stress-related hormones from the adrenal glands, reduces cholesterol levels	[2,3,5,6,7]
Vitamin B6	11.90 mg/100 g		Plays a role in cognitive development through the biosynthesis of neurotransmitters and in maintaining normal levels of homocysteine, an amino acid in the blood, gluconeogenesis and glycogenolysis, immune function, and hemoglobin formation, transamination, and decarboxylation of amino acids	[2,3,5,6,7]
Vitamin B9 (Folic acid)	0.40 mg/100 g		Helps to form DNA, RNA, and protein metabolism, rapid growth during pregnancy and fetal development, supports healthy blood cells, plays a key role in breaking down homocysteine, DNA biosynthesis, and methylation	[2,3,5,6,7]
Vitamin B12	0.15 mg/100 g		Plays an essential role in red blood cell formation, cell metabolism, nerve function, and the production of DNA, the molecules inside cells that carry genetic information	[2,3,5,6,7]
Vitamin C (Ascorbic acid)	2.00 mg/100 g		Antioxidant activity, form blood vessels, cartilage, muscle, and collagen in bones	[2,3,5,6,7]
Vitamin D	0.2 mg/100 g		Calcium and phosphorus absorption, essential for the bones and teeth, the immune system, brain health, and for regulating inflammation	[2,3,5,6,7]
Vitamin E	5.00 mg/100 g		Antioxidant activity	[2,3,5,6,7]
**Free amino acid**				
Lysine	62.43 mg/100 g		Prevent chronic fatigue, help maintain skin, hair, nails, bones, joints, hormonal regulation, sexual vitality, weight regulation, body vitality, recovery from illness, stimulation of the immune system, health of the cardiovascular system	[3,57,64,65]
Proline	58.76 mg/100 g			
Cysteine	21.76 mg/100 g			
aspartic acid	17.33 mg/100 g			
valine, glutamic acid, serine, glycine, cysteine, threonine, alanine, tyrosine, phenylalanine, hydroxyproline, leucine-isoleucine, and glutamine	less than 5 mg/100 g			
**Minerals**				
Macroelements	321.1–357.4 mg/100 g			
Na	0.30–13.8 mg/100 g		Helps maintain normal blood pressure, supports the work of your nerves and muscles, and regulates your body’s fluid balance	[54,55]
K	321.1–357.4 mg/100 g		Regulates fluid balance, decreases blood pressure, regulates electrical activity of muscle cells and heart, and improves bone mineral density	[53,54]
Ca	22.8–24.0 mg/100	14.5–113	Effect on bone mineral density and bone mineral content, prevent fat accumulation, regulate blood pressure and premenstrual syndrome, and reduce the risk of colon cancer	[57,58]
Mg	44.0–50.4 mg/100		Helps with high blood pressure, cardiovascular diseases, osteoporosis and diabetes, reduces the inflammatory processes, improves glucose and insulin metabolism, and normalizes the lipid profile	[52,53,54,55,56]
P	338.4–412.1 mg/100		Plays key roles in regulation of gene transcription, activation of enzymes, maintenance of normal pH in extracellular fluid, and intracellular energy storage, helps in the formation of bones and teeth	[52,53,54,55,56]
S	153.2–169.3 mg/100		Plays role in formation of collagen and keratin proteins, responsible for wound healing and for the health of the skin, hair, nails, and connective tissue, is important for proper development of cartilage and tendons, participates in detoxification processes, blood coagulation, and the production of some bile of acids	[52,53,54,55,56]
Microelements				
Fe	n.d.		Oxygen transport, DNA synthesis, and electron transport, metabolic processes and energy, regulates macrophage polarization, activity and function of neutrophils, NK, T, and B cells	[3,11,50,52]
Mn	0.01–0.08 mg/100		Oxidative phosphorylation, fatty acids, and cholesterol metabolism, mucopolysaccharide metabolism, and urea cycle	[3,11,52]
Zn	2.07–2.58 mg/100		Supports growth, development, and immune functions	[3,11,52]
Cr	0.03–0.15 mg/100		Helps control whole body metabolism, utilizes energy, controls body sugar, and body function.	[3,11,52]
Cu	0.31–0.39 mg/100		Supports energy production, iron metabolism, neuropeptide activation, connective tissue synthesis, and neurotransmitter synthesis, angiogenesis, neurohormone homeostasis, and regulation of gene expression, brain development, pigmentation, and immune system functioning, defense against oxidative damage	[3,11,50,52]

**Table 3:** Overview of biological and pharmacological effects of royal jelly shown in cell cultures and animal models.

Effect	References
**Antibacterial, antiviral, antiparasitic effect** **(model—microrganisms)**	
Antibacterial	[48,294,300,301,302]
Antifungal (fungicidal)	[3,7,48,57,295,304]
Antiviral	[293,305,306,307,308,309,310,311]
Activity against different parasites of family *Trypanosomidae*	[306]
**Bio-stimulatory effect and anti-ageing effect** **(model cell culture and laboratory animals)**	
Estrogenic and gonadotropic effects confirmed on cells and in rats	[28,86,207]
Increase in growth and animal weight	[52,108,109,110,291,345]
Anticancer effects, increases immune cells’ activity and stress resistance	[36,37,226,227,228,229,230,232,243,246,254]
Increases reproduction capacity in rats and sheep	[121,209,211,212,213,214]
In vitro increases oxygen consumption in tissues, antihypoxic effect	[180,191,250]
Against male rabbits’ infertility, increases sexual effectiveness in rats and hamsters	[55,59,212]
Prolongs lifespan in rats and hamsters	[39,104,105,106,107,129]
**Immunomodulating effects: anticancer, antiallergic, and anti-inflammatory** **(model—laboratory animals and humans)**	
Immunostimulant activity, increase in number of leukocytes	[68,77,125,230,246,268]
Anticancer effects	[34,35,36,37,38,39,40,42,44,49,50]
Prevents autoimmunity in mice	[335,336,337]
Anti-inflammatory effects	[7,13,36,68,69,70,127,137,262,271,304,334]
**Heart and circulatory system effects** **(model—laboratory animals and humans)**	
Decreases increased blood pressure, hypotensive effect, vasodilatation	[53,54,55,56,58,59,177,187,190,191,193,194,195,196,197]
Antiatherosclerotic effect: decreases cholesterol and triglyceride concentration in blood, increases HDL, decreases fibrinogen in plasma and thrombosis	[27,28,127,143,150,172,173,174,187,203,308]
Cardio-protective effect, prevents myocarditis	[58,77,78,79,80,92,93,100,101,102,103,172,173,178,202,345]
Increases thyroxin levels, albumin/globulin ratio in blood, and decreases serum proteins after oral application in rats	[75,76,113]
**Effects on central and vegetative nervous system** **(model—laboratory animals)**	
Effects on central nervous activity—protection and activation	[28,29]
Increases brain cell differentiation	[154,156,157]
Improves memory and learning through upregulation of neurotrophic factor synthesis such as that of GDNF and other neurotrophins	[137,139,156,157,176]
Acetyl-choline-like effects on bowels and smooth muscles nerves	[137,154,156,176,177,197]
Calming effects (rat)	[112,157]
**Antioxidative, hepatoprotective and radio protective effects** **(model—laboratory animals)**	
Antioxidative effect	[52,58,62,64,67,83,103,108,127,147]
Liver protection	[81,82,83,92,100,240,263,269,290]
Reducing stress and teratogen effects, lung edema, and liver or kidney damage after mycotoxine intake in rats	[67,98,99,100,101,102,107]
Stimulates DNA synthesis in hepatocytes and protects cells from apoptosis and mitogen effect, prolongs cell proliferation, and increases albumin production	[26,113]
Radiation protective effect in animal experiments	[247,249,250,278,279,280,281,282,283]
**Other effects** **(model—laboratory animals and humans)**	
Prevents osteoporosis and promotes bone formation	[87,127,204,205,206,207,208,209,210,211,212,213,214,215,216,217,218,219,220]
Skin protection: promotes collagen build-up	[114,117,118,119,120,131,304,318,325,334]
Hyperglycemic effect, prevents insulin resistance, antidiabetic effect	[166,167,169,174,184]
Decreases experimental colitis in rats	[61,62]
Antiallergic activity, decreases development of atopic dermatitis as skin lesions in rats	[334,340]

**Table 4:** Medical effects of royal jelly in humans.

Usage	References
**Pediatrics:**	
Pre-term babies or inadequate food intake: improves overall condition, increase in weight, appetite, red blood cells, and hemoglobin	[6,112]
**Geriatrics:**	
Increase in overall well-being and recuperation from fatigue and menopausal problems	[90,121,202,211,223,224]
Against stenocardia and after heart attack; arteriosclerosis and atherosclerosis; hypertension	[187,188,190,195,197,202,203,204]
Against respiratory system diseases, asthma	[333,338,339]
Against diseases of the eye, e.g., blepharitis, conjunctivitis and retina burns, circulatory disorders in the eye	[97,126,161]
Bio-stimulating effect, increases physical endurance and work ability and increases resistance to hypoxia	[52,124,125,152]
Increase memory, neuro-vegetative activation	[137,138,141,145,147,154,157]
Anti diabetes 1	[162,163,164,165,166,167,168,173,174]
Anticancer effects	[77,89]
Prevention of stomach and duodenum ulcer, stomach problems	[175,176,177]
Promote skin regeneration and skin lesion healing	[114,117,131,304,318,334]
Prevent degenerative processes and rheumatism	[85,88]
Prevent warts, acne, ulcers, seborea, neurodermatitis	[280,284,334]
Prevent kidney dysfunction	[264,265,269,288]

**Table 5:** Overview of the molecular and cellular effects of RJ and its biologically active components.

Biological Activity	Active Component	Molecular and Cellular Effect of RJ	References
**Cellular senescence**			
Immune system			
	RJ and its components	– stimulate immune surveillance and phagocytic potential – stimulate stress-induced replication – increase response of effector cells (T, B, macrophages, NK) – decrease risk of chronic, autoimmune diseases and cancer – increase number of hematopoietic stem cells in peripheral blood	[124,125] [98,132,133,134] [101,102]
Nervous system			
	RJ and its components	– decrease senescence in glial cells (microglia and astrocytes) – reduce neurotoxicity and accumulation of senescent microglial cells – decrease risk of neurodegenerative diseases	[52,110]
Cardiovascular system			
	RJ and its components	– decrease number of senescent cells – prevent changes of cellular signaling and maintain homeostasis in stressful conditions – reduce SASP induction – decrease risk of CVD	[98,132,133,134]
Genes expresion and pathways involved in senescence			
	MRP1	– prevents disruption of tumor suppressors (p53, p16, RB, PTEN) – regulates activation of cell cycle effectors (cyclins, CDKs) – regulates DNA damage response (ATR, ATM, Chk1) – acts on epigenetic changes and epigenetics regulators – upregulates SOD1 and downregulates mTOR, CTNNB1, and TP53, and has a stimulatory effect on DNA and protein syntheses – inhibits SASP pro-inflammatory factors, mostly NF-kB-dependent factors such as IL-6, IL-8, IL-1β, MCP-1, MCP-2 and MCP-4, HGF, FGF, and MMPs – upregulates S6K1, MAPK, and EGFR in the EGFR-mediated signaling – activates the Nrf2–HO-1 pathway – reduces expression of the proinflammatory cytokines (IL-1, IL-6, COX-2, and MCP-1) – reduces activation of NF-κB and c-Jun N-terminal kinase (JNK) pathways	[98,102,110] [111] [115] [129,130,131,132] [43,44,105,106,107] [132,133,134] [109,110,117,118,119,120,121]
**Cognition and AD-related pathology**			
	RJ and its lipids	– alleviate Aβ pathology (reduce Aβ influx through the BBB via RAGE inhibition) – prevent cleavage of APP into Aβ by inhibiting BACE1 – facilitate degradation and clearance of Aβ by IDE, NEP, and LRP1 – activate AMPK and inhibits mTOR pathway, and promote autophagy and antioxidant production – suppress microglial inflammation by inhibiting various oxidative, inflammatory, and apoptotic pathways, e.g., iNOS and NF-κB – enhance production of neurotrophins such as NGF and BDNF by binding to estrogen receptors β and α – promote ACh production, neurogenesis, and synaptogenesis	[58,139,147] [58,135,136,137,139,140] [145,150] [58,145,146] [58] [146,155] [58,138,139,140,141,142,143,144,145,146,147,148,149,150,151,152,156] [147,154,156] [154,156]
	MRJPs	– improve brain metabolism by increasing levels of glucose and phosphoenolpyruvic acid – increase the level of nicotinic acid mononucleotide (NaMN), a precursor of NAD+, and xanthosine, which sustains the nucleic acid metabolism and supports DNA repair in aged rats – stimulate production of neuroprotective molecules in aged rats, mainly of cysteic acid – enhance memory	[139,159]
**Aging and longetivity**			
	MRJPs	– upregulate S6K1, MAPK, and EGFR in the EGFR-mediated signaling – increase telomere length, decrease senescence, and stimulate proliferation in human embryonic lung fibroblasts (HFL1 cell line) by downregulating p53, catenin beta like-1, and mTOR pathways – increase SOD1 gene expression and decrease levels of malonaldehyde, an oxidative stress marker	[11,111] [101,102,123] [110]
**Anti-diabetic**			
	RJ	– increases insulin concentration and levels of albumin and total proteins in type 2 diabetes – increases serum apolipoproteins A-I (Apo-A-I), changes levels of apolipoproteins B and ratio of Apo-B and Apo-A-I – reduces FBS, HbA1c, and HOMA-IR – improves serum levels of triglycerides, cholesterol, HDL, LDL, VLDL, and Apo-A1 – reduces oxidative stress – increases antioxidant enzymes and total antioxidant capacity – regulates glycemic parameters (fasting blood glucose and glucose clearance as the most affected parameters) – reduces AST, ALT, ALP – maintains homeostasis by decreasing insulin resistance – decreases mRNA expression of glucose-6-phosphatase during long-term application – increases expression of adiponectin and adiponectin receptor 1 mRNA and pAMPK protein – improves hyperglycemia and insulin resistance by activating the expression of PGC-1α – improves hyperglycemia and partially reduces body weight in obese/diabetic KK-Ay mice – promotes expression of peroxisome proliferator-activated receptor-α (PPARα) and peroxisome proliferator-activated receptor-γ coactivator-1α (Pgc-1α) which improves lipid utilization and reduces body weight in KK-Ay mice – facilitates and enhances GLUT4 translocation	[170,171] [167,172] [28,127,172,173,174,203] [173] [167,168,169] [188] [183,189] [84,188,189] [181] [183] [184] [168]
**Obesity**			
	HDAA HDEA	– decrease obesity – decrease body fat accumulation – decrease total cholesterol – decrease insulin resistance – decreas C-reactive protein – increases adiponectin, total antioxidant capacity, endogenous antioxidants, bilirubin, and leptin – improve thermogenesis in brown adipose tissue (BAT) – increases UCP1 – reduce hyperglycemia and liver steatosis – increases irisin and metabolic thermogenesis in brown adipose tissues	[182,183,184,185,186] [184] [189] [182] [186] [185]
**Hypotensive and hypolipidemic activity**			
	MRJP1, MRJP2, and MRJP3	– interact with bile acids, enhance the excretion of fecal bile acids, increase fecal excretion of cholesterol, and increase the hepatic cholesterol catabolism – show hypotensive effect acting on VSMCs, which regulate blood pressure – peptides derived from MRJP1 after gastrointestinal digestion show ACE inhibitory activity – RJ proteins, including royalisin and degradation products of MRJP1 and MRJP3 inhibit macrophage proliferation in atherosclerotic plaques	[187,188,197] [197,198] [190,196,200] [199]
	Peptides (Ile-Tyr, Val-Tyr, and Ile-Val-Tyr) formed by hydrolysis of RJ by protease N	– inhibit ACE activity – have anti-hypertensive effects by decreasing systolic blood pressure	[190]
	Peptides derived from MRJP1	– have strong ACE inhibitory activity – degradation products of MRJP1 and MRJP3 bind LDL-C and oxidized LDL-C components reducing atherosclerotic lesions – promote regression of atherosclerotic plaque by lowering plaque inflammation	[357] [28] [199,202,203,204]
**Anti-inflammatory**			
	MRJP1 MRJP2, MRJP3, 10-HDA	– increase macrophage stimulation – inhibit activation of p38 and JNK/AP-1 signaling pathways – decrease production of IgE and IgG1 – decrease levels of MMP-1, MMP-3, and p38 – inhibit lipolysaccharide (LPS)-induced production of inhibitor of kappa-B-zeta (IkB-z) and IL-6 – inhibit NF-κB and JNK pathways – inhibit production of TNF-α, IL-1β, IL-8, and NF-κB – reduce expression of the proinflammatory cytokines (IL-1, IL-6, COX-2, and MCP-1) – 10-HDA acts as histone deacetylase inhibitor (HDACI) and inhibits proliferation of FLS cells induced by RA	[62,63,68,75] [85] [13,69] [85,88]
**Inflammatory bowel diseases**	RJ	– improves the colonic mucosal barrier and intestinal gut microbiota – reduces number of CD3+, CD5+, CD8+, and CD45+ T cells; secretion of pro-inflammatory cytokines IL-1β, TNF-α, COX-2, and NF-κB in TNBS-induced rat colitis model – alleviates the ulcerative erosion of colon tissue and number of colonic CD3+, CD45+ T cells, and mast cells in acetic-acid-induced colitis model – increases production of anti-inflammatory cytokine IL-10 and GPx activity – decreases intestinal permeability by increasing the expression of tight-junction proteins, goblet cells, and mucin MUC2 levels in DSS-induced ulcerative colitis model – reduces expression of proinflammatory cytokine IL-6 and increases expression of anti-inflammatory cytokine IL-10 and sIgA – increases immunity and antioxidant activity, and regulates composition and structure of the gut microbiota – improves gut dysbiosis and inflammation of the intestinal mucosa, regulates expression of nutrient absorption-related genes in the small intestine, increases excretion of saturated fatty acids in feces, and increases SCFA-producing intestinal bacteria – acts on the suppression of the secretion of pro-inflammatory cytokines such as IL1, TNF-α, and IL12, which affect number of innate lymphoid cells (ILC1 and ILC3) and their cytokines production (TNF-α, IFN-γ, IL-17, and IL-22)	[72,73,74] [77,78,79,80,81,82,83,84,85,86,87,88,89] [77,78]
	MRJP1, MRJP2, and MRJP3	– stimulate mouse macrophages and promote the immune response – improve the quantity of peripheral blood leukocytes, immunoglobulin content, immune factor levels, and the proliferation ability of spleen lymphocytes	[76]
**Non-alcoholic fatty liver disease (NAFLD** **) and non-alcoholic steatohepatitis (NASH)**	RJ and its 10-HDA, 10-HDAA, 2-DA, and SA	– improve adverse effects of estrogen deficiency, including body and liver weight gain, mental disorders, serum lipid profile abnormalities, liver lipid deposition, lipid peroxidation, and circadian gene expression disorders – decrease body and liver weights, improve cholesterol levels, downregulate ALT and AST levels, and combat NAFLD in OVX rats – attenuate the hepatic steatosis and liver injury – demonstrate strong anti-oxidative activity and capacity to ameliorate the disturbances of the circadian genes, *Per1* and *Per2*, provide quick energy to the liver, which reduces TC deposition and dyslipidemia – regulate activation of ERβ – decrease TC and LDL-C levels and increase HDL-C	[80,81,82,83,84,85] [82] [35]
**Rheumatoid arthritis (RA)**	10-HDA	– inhibits activity of MMP-1, MMP-3, and p38 and JNK–AP-1 signaling pathways – inhibits TNF-α-induced expression of inflammatory cytokines and ECM-degrading enzymes – prevents cell proliferation of fibroblast-like synoviocytes by inhibiting target genes of PI3K–AKT pathway and genes of cytokine–cytokine receptor interactions – enhances Nrf2 translocation to the nucleus, activating the gene expression of the antioxidant and detoxifying enzymes	[86] [89]
**Multiple sclerosis**	RJ and 10-HDA	– reduce demyelination, modulate the inflammatory response by acting on the polarization of Th17 and Th1 cells, and reduce the infiltration of inflammatory cells in the CNS	[90,91]
**Herpes stromal keratitis (HSK)**	RJ and MRJP3 or its C-terminal tandem pentapeptide repeats (TPRs) sequence	– inhibit production of pro-inflammatory cytokines TNF-α, IL-1β, IL-2, IL-6, and IL-33	[92,93,363]
**Anti-cancer**			
	MRJPs MRJP-1 10-HDA	– decrease activity of MMP-9, AKT, and MAPK – increase levels of caspase 3 and 9, and Bax – inhibit metabolism of 2-aminofluorene (2-AF) metabolites in a human liver tumor cell line and decrease the 2-AF in J5 cells – inhibit tumor-induced angiogenesis and/or the activation of immune function – inhibit proliferation of B16F1 melanoma cells by inhibiting the expression of microphthalmia-associated transcription factor, tyrosinase-related protein 1 (TRP-1) and TRP-2, and inhibit melanin production, then inhibit skin pigmentation in mice – induce apoptosis of A549 human lung cancer cells through the ROS-ERK-p38 and CHOP pathways – inhibit production of TNF-α, IL-1β, IL-8, and NF-κB in WiDr cells – reduce Bcl-2 and Ki-67 expression, enhance p53 and caspase 3/7 – inhibit the expression of Bcl-2 and p53 in HepG2 cells – reduce the paraneoplastic syndrome in RCC patients by decreasing concentration of TNF-α and TGF-β – acts as a potent HDAC inhibitor	[232,252] [242,243] [240] [232] [233] [234,235] [68] [275] [241] [252] [47,88]
**Anti-oxidative**			
	MRJP-2 dipeptides Free amino acids	– increase SOD, CAT, GR, and GPx activities and GSH levels – decrease levels of ROS – decrease MMPs, MDA, and NO – upregulate Nrf2 pathway – enhance HO-1 expression	[62,63,64,65,66,67,68] [85] [67,88] [67]
	4-hydroperoxy-2-decenoic acid ethyl ester (HPO-DAEE), and RJ fatty acid derivative	– increases the expression of HO-1 mRNA by activating Nrf2 signaling pathway in human neuroblastoma SH-SY5Y cells – promotes phosphorylation of eukaryotic initiation factor 2α (eIF2α) and nuclear accumulation of the activating transcription factor-4 (ATF4) – suppresses UVA-induced expression of MMP-1 and MMP-3 by inhibiting the activation of JNK and p38-MAPK pathways in human dermal fibroblasts – suppresses the expression of tyrosinase-related protein (TRP)-1 and TRP-2, which are oxidases and rate-limiting enzymes that regulate melanin production	[67,68] [237,238] [98] [117,224] [233]
**Immunoregulatory activity**			
	RJ and 10-HDA	– directly activate the immune functions of macrophages, T lymphocytes, B lymphocytes, and natural killer cells – promote macrophage recovery, their number, and activity – modulate innate immunity through IIS/DAF-16, p38 MAPK, and Wnt signaling pathways – stimulate production of cytokines, enabling regulation of the immune response – restore the proliferation of thymus and spleen cells – stimulate the hematopoietic function of the spleen and the survival of mice by reducing the level of prostaglandin which regulates the proliferation of lymphocytes and inhibits the tumoricidal activity of normal macrophages – increase plaque-forming splenocytes, antibody production, and immunocompetent cell proliferation – modulate immune responses via downregulation of NLRP1 – induce maturation of immune cells, stimulate innate and adaptive immune responses	[226] [36,226,227,228,229,230,246] [125] [246] [229,243,244,245] [230] [13] [76,238,243,246,250] [267,268]
	MRP3	– suppresses production of IL-4, inhibits serum levels of anti-OVA IgE and IgG1	[37]
	MRJPs	– increase immunoglobulin content, immune factors levels, and proliferation of spleen lymphocytes	[74,77,246]
**Anti-allergic effect**			
	RJ	– inhibits mast cell degranulation, suppresses cysteinyl-leukotriene release, reduces serum histamine, IgG, and IgE levels in various allergic conditions by suppressing expression of histamine H1 receptor – restores the macrophage function and improves Th1/Th2 cell response – enhances antigen-specific mucosal IgA response	[37,333,334,335,336,337,338] [333] [339]
**Osteoporosis**			
	10-HDA	– inhibits osteoclast differentiation and function by suppression of the NF-kB signaling pathway and its downstream molecules including NFATc1, CtsK, TRAP, V-ATPase D2, and MMP9, via FFAR4 – decreases RANK, RANKL, NF-κB, IL-1β,TNF-α, caspase 3, MMP9 and CTX-1, and number of mature osteoclast – increases ALP, RUNX2, OSX, collagen type 1, OPG – increases proliferation, osteoblast differentiation, and bone mineralization – increases bone strength	[206] [206,222] [206,220,221] [37,66,86,121,223,224,225]
	RJ and RJP	– enhance intestinal calcium absorption – RJP prevents bone abnormality caused by sex hormone deficiency – Inhibit RANKL-induced osteoclastogenesis	[220,221]
**Estrogen effect**			
	RJ and 10-HDA	– improves regularity of the estrus cycle, ovarian histology and function, and level of reproductive hormones (LH, T, FSH, E2) – improves ovarian oxidant–antioxidant status (MDA, TAC, GPx) – increases reproductive potential, follicular growth, and estradiol secretion	[121,211,212,213,214] [212,213] [208,209,210,211]
**Spematogenesis**			
	RJ	– increases cellular antioxidant activity and levels of detoxifying molecules, including GSH, GPx, GR, SOD, CAT, and Nrf2 – preserves percentage of viable spermatozoa and glutathione levels during exposure to toxic metals and pro-oxidants – regulates levels of FSH, LH, TSH, T4, T3, and testosterone – protects testicular structure from the damaging effect of diabetic oxidative stress and preserve male fertility through its antioxidant effect	[215,216] [217] [217,218,219]
**Growth-promoting and wound healing activities**			
	RJ MRJP1	– stimulate growth of human lymphoid cell lines (U-937, THP-1, U-M, HB4C5, HF10B4) – RJ induces proliferation of human cell lines and partially replaces FBS – upregulates SOD1, mTOR, and catenin beta like-1, and downregulates p53 – promote wound repair – inhibit production of proinflammatory cytokine and NO – show scavenging activity – reduce MPO activity – posseses a healing effect against the chemotherapy- and radiotherapy-induced oral mucositis	[21,26,33,111,246,332] [36,313,314,315,316,317,318,319,320,321,322,323,324,325] [111] [313,314,315] [313,314,327,329,330] [326,327,328,329,330,331]
	SA, 10-HDA, and 10-HDAA	– have anti-inflammatory activity	[53]
	Royalactin, royalisin, 10-HDA	– show regenerative ability	[44,105,313,314,315,316,317,318,319,320,321,322,323,324,325]
	MRJP mixture (MRP1-MRP7)	– increases proliferation, minimizes senescence, and elongates telomeres – increases viability by protecting NIH-3T3 cells against oxidative stress-induced cell apoptosis – activates a pluripotency gene network that enables self-renewal in mouse embryonic stem cells (mESC) – promotes wound healing by inducing proliferation and migration of human epidermal keratinocytes – contribute to tissue regeneration and wound closure	[26,111,313,314,315,316,317,318,319,320,321,322,323,324,325] [29] [44] [26] [313,314,315]
**Antimicrobial activity**			
	cysteine-rich AMPs and royalisin Jelleine-I-IV Chlorine-jelleine-I (Cl-J-I), bromine-jelleine-I (Br-J-I), and iodine-jelleine-I (I-J-I), MRJP1	– interact with membrane enzymes and proteins and cause an opposite flow of protons, affect cellular activity including energy production (membrane-coupled), membrane transport, metabolic regulatory functions, and synthesis of DNA and RNA, prevent protein translation, and suppress microbial virulence factors – participate in membrane permeabilization of the bacterial cell – increase total antibody and isotypes of antibodies such as IgM, IgG1, IgG2a, and IgG2b	[48,63,68,292,293,294,295,296,297,298,299,300] [301,304,305] [307]
	glucose oxidase enzyme (GOx)	– catalyzes oxidation of glucose to hydrogen peroxide which shows prominent antibacterial effect	[292,298,299]
**Antiviral activity**		-	
	10-HDA	– promotes human monocyte-derived dendritic cells to produce Th1 cytokine pattern – stimulates B lymphocytes to produce antibodies against HCV and HIV	[48,63,68,292,293,294,295] [307]
	MRJPs MRJP2 and MRJP2 X1 MRJP1	– exert an inhibitory influence, via different mechanisms, for SARS-CoV-2 non-structural proteins (main and papain proteases, RNA replicase, RNA-dependent RNA polymerase, and methyltransferase). – can bind to most of the oxy- and deoxyhemoglobin-binding sites or cofactor-binding site residues on the viral nsp3, nsp5, nsp9, nsp12, and nsp16 and prevent their hemoglobin attack – promote Th1 immune polarization (IFN-γ activates TCD8+ and TCD4+ lymphocytes against viral infections to eliminate infected cells)	[305,306,307,308,309,310,311] [308] [307]
	RJ	– reduces oxidative stress and levels of IL-6, IL-8, and TNF-α	[292,298,299,327,328,329,330,331]

Note: Aβ, amyloid beta; ACE, angiotensin-converting enzyme; Ach, acetylcholine; Akt, protein kinase B; ALP, alkaline phosphatase; ALT, alanin-aminotransferase; AMPK, AMP-activated protein kinase; anti-OVA, antibodies that detect ovalbumin; AP-1, Activator protein 1; ApoA-I, apolipoprotein A-I; Apo-B, apolipoprotein B; APP, amyloid precursor protein; ARE, antioxidant response element; AST, aspartate aminotransferase; ATF4, activating transcription factor-4; ATM, ataxia telangiectasia-mutated; ATR, ataxia telangiectasia-mutated and Rad3-related; BACE1, beta-site APP cleaving enzyme 1; BAT, brown adipose tissue; Bax, Bcl-2-associated X protein; BBB, blood–brain barrier; Bcl-2, B-cell lymphoma-2; BDNF, brain-derived neurotrophic factor; BMD, bone mineral density; CAT, catalase; CDKs, cyclin-dependent kinases; C/EBP, CCAAT/enhancer-binding protein; Chk1, checkpoint kinase 1; CHOP, C/EBP homologous protein; CNS, central nervous system; COX-2, Cyclooxygenase-2; CTNNB1, Catenin beta-1; CTSK, cathepsin K; CTX, C-terminal telopeptide; CVDs, cardiovascular diseases; DAF-16/FoxO, DAF-16, forkhead box o; DSS, dextran sulfate sodium; E2, estradiol; ECM, extracellular matrix; EGFR, epidermal growth factor receptor; eIF2α, eukaryotic initiation factor 2α; ERα, estrogen receptors alpha; ERβ, estrogen receptor beta; ERK, extracellular signal-regulated kinase; FBS, fetal bovine serum; FFAR4, free fatty acid receptor 4; FGF, fibroblast growth factor; FLS, fibroblast-like synoviocytes; FSH, follicle-stimulating hormone; GLUT-4, glucose transporter 4; GPx, glutathione peroxidase; GR, glutathione reductase; GSH, glutathione; HGF, human growth factor; HbA1c, glycated hemoglobin; HCV, hepatitis C virus; HDACI, histone deacetylase inhibitor; HDL-C, high-density lipoprotein cholesterol; HIV, human immunodeficiency virus; HO-1, heme oxygenase 1; HOMA-IR, Homeostatic Model Assessment for Insulin Resistance; IDE, insulin-degrading enzyme; IgE, immunoglobulin E; IgG1, immunoglobulin G1; IGH, insulin-like growth factor; IκB-ζ, induced inhibitor of kappa-B-zeta; IL-1β interleukin 1 beta; ILC1, innate lymphoid cells 1; ILC3, innate lymphoid cells 3; iNOS, Inducible nitric oxide synthase; IRS, insulin receptor substrate; JNK, c-Jun N-terminal kinase; LDL-C, low-density lipoprotein cholesterol; LH, luteinizing hormone; LPS, lipopolysaccharide; LRP-1, low-density lipoprotein receptor-related protein 1; MAPK, mitogen-activated protein kinase; MCP-1, monocyte chemoattractant protein-1; MCP-2, monocyte chemoattractant protein-2; MCP-4, monocyte chemoattractant protein-4; MDA, malondialdehyde; mESCs, mouse embryonic stem cells; MMP-1, matrix metalloproteinase 1; MMP-3, matrix metalloproteinase 3; MMP-9, matrix metalloproteinase 9; MMPs, matrix metalloproteinases; MPO, myeloperoxidase; mTOR, mammalian target of rapamycin; MUC2, mucin; NaMN, nicotinic acid mononucleotide; NEP, neprilysin; NFAT c1, nuclear factor of activated T cell; NF-κB, nuclear factor kappa-light-chain-enhancer of activated B cells; NGF, nerve growth factor; NLRP1, NOD-like receptor (NLR) family pyrin domain-containing 1; NO, nitric oxide; Nrf2, nuclear factor E2-related factor 2; OPG, osteoprotegerin; Osx, Osterix; OVX, ovariectomy; PGC-1α, peroxisome proliferator-activated receptor-γ coactivator-1 alpha; PI3K, phosphatidylinositol 3-kinase; PPAR-γ, peroxisome proliferator-activated receptor gamma; PTEN, phosphatase and tensin homolog; RAGE, receptor for advanced glycation end products; RANK, receptor activator of nuclear factor-κB; RANKL, receptor activator of NF-κB ligand; *RB*, retinoblastoma gene; RCC, renal cell carcinoma; ROS, reactive oxygen species; RUNX2, runt-related transcription factor 2; S6K1, S6 kinase 1; SASP, senescence-associated secretory phenotype; SCFAs, short-chain fatty acids; SOD, superoxide dismutase; T, Testosterone; T3, triiodothyronine; T4, thyroxine; TC, total cholesterol; TGF-β, transforming growth factor-beta; TNF-α, Tumor Necrosis Factor alpha; TNBS, 2, 4,6-trinitrobenzene sulfonic acid; TRP-1, tyrosinase-related protein 1; TRP-2, tyrosinase-related protein 2; TSH, thyroid stimulating hormone; UCP1, uncoupling protein 1; VLDL-C, very-low-density lipoprotein cholesterol; VSMCs, vascular smooth muscle cells.

**Table 6:** Dosage of Royal Jelly for human use—clinical trials.

Application	Dose/Period of Treatment	Conditions/Diseases	References
**Infants:**	0.5 g/day for 2–12 months	– growth and development – strengthen immunity and nervous system	[6]
For premature babies	50 mg to 1 g per day	– growth and development – strengthen immunity and nervous system	[6]
**Children:** Children aged 1–5 yrs old Children aged 5–12 yrs old	0.5 g/day 0.5–1 g/day different period, condition-depedent	– low immune system – nervous system impairment (foetal suffering, delivery complication when born) – weakness, loss of appetite, anorexia, – anemia, etc.	[6]
Children aged 1–5 yrs old Children aged 5–12 yrs old	2.5 g/day, 1–3 days 5 g/day, 1–3 days	– acute infection and colds	[6]
Children	2 g, 3 months	– reduces the consequences of SLE	[336]
**Adults:**	1 to 2 g/day	– immunity – insomnia – skin disorders, rejuvenation – for maintenance of skin, hair, nails, bones, joints – anemia – low libido, sexual vitality – hormonal imbalance, wounds – premenstrual syndrome, menopause, osteoporosis, etc. – mental condition, memory, depression	[6,29,36]
	3–5 g/day	– diabetes – depression, Hashimoto’s disease, – arthritis	[6]
	3000 mg, 6 months	– Osteoporosis, improved BMD, and strength in postmenopausal women	[223]
	Up to 10 g/day for 1–3 days	– in the beginning of colds	[6]
	10 g/day for 1–3 days 5–10 g/day up to 3–5 days	– in acute infections – to accelerate post-operative healing	[6]
	1000 mg, 8 weeks	– Menopausal symptoms	[91,211,224,225]
	5000, 8 weeks	– Sub-fertility	[6,364]
	1000 mg, 8 weeks	– Type 2 diabetes mellitus – high blood pressure	[365]
	3600 mg, 24 months	– Hemodialysis	[366]
	500 mg, 3 weeks	– Multiple sclerosis	[90]
	10–15 g/day, long-term	– Neurodegenerative diseases, multiple sclerosis, Parkins, anti-depressive, anti-anxiety	[6,36]
	800 mg, 12 weeks	– Dry mouth sensations	[367]
	3000 mg, 2 weeks	– Traumatic brain injury	[368]
	100 mg, 8 weeks	– Infertility, impotence, hormonal regulator	[369]
	1000 mg, 8 weeks	– Genitourinary syndrome	[91,92]
	900 mg, 13 weeks	– Metastatic renal cell carcinoma	[252]
	3000 mg, 6–8 weeks	– Side effects of chemotherapy and radiotherapy such as paresthesia, pain or burning, sensation of fingers, imbalance during walking, the sensation of weakness in the legs – chronic fatigue – recovery from disease – immunological system stimulation	[252,264,281]
